# Potential interactions between Mesolithic hunter-gatherers and Neolithic farmers in the Western Mediterranean: The geochronological data revisited

**DOI:** 10.1371/journal.pone.0246964

**Published:** 2021-03-03

**Authors:** Thomas Perrin, Claire Manen

**Affiliations:** UMR5608 TRACES, CNRS, Toulouse Jean-Jaurès University, Toulouse, France; University at Buffalo - The State University of New York, UNITED STATES

## Abstract

In the Western Mediterranean, the Neolithic mainly developed and expanded during the sixth millennium BCE. In these early phases, it generally spread through the displacement of human groups, sometimes over long distances, as shown, for example, by the Impressa sites documented on the northern shores. These groups then settled new territories which they gradually appropriated and exploited. The question of their potential interaction with groups of Late Mesolithic hunter-gatherers living in the area prior to their arrival is therefore crucial. Were their encounters based on conflict and resistance or, on the contrary, on exchange and reciprocity? Many hypotheses have been put forward on this matter and many papers written. Before we can consider these potential interactions however, we must first ascertain that these different human groups really did meet—an implicit assumption in all these studies, which is, in reality, much less certain than one might think. The population density of the Late Mesolithic groups varied greatly throughout the Mediterranean, and it is possible that some areas were relatively devoid of human presence. Before any Neolithization scenarios can be considered, we must therefore first determine exactly which human groups were present in a given territory at a given time. The precise mapping of sites and the chronological modeling of their occupation enriches our understanding of the Neolithization process by allowing high-resolution regional models to be developed, which alone can determine the timing of potential interactions between Mesolithic and Neolithic groups. Various international research programs have recently produced several hundred new radiocarbon dates, based on selected samples from controlled contexts. The geochronological modelling of these data at the scale of the Western Mediterranean shows contrasting situations, probably related to different social and environmental processes. These results suggest that we should consider a varied range of Neolithization mechanisms, rather than uniform or even binary models.

## Introduction

The emergence and development of agro-pastoral societies is one of the major changes in the history of mankind. After several millennia during which populations survived mainly thanks to the sustainable use of the resources available in their surrounding environment, they suddenly (on the scale of the history of humanity) become the producers of the bulk of their subsistence—for better or for worse. While food resources were at the very heart of this Neolithic revolution, its repercussions were numerous and overthrew the entire social organization of these populations, their way of thinking beyond their “simple” way of life, their symbolic worlds, and so forth (for recent global approaches, cf. for example [1–3]). One of the salient features of these changes is not only its inevitability, as history has shown, and often even its irreversibility, but also the many different hotbeds of invention and innovation that sprung up across the globe. Even if their asynchronous aspect could suggest spreading from a single point of origin, this was not the case—there is simply no evidence of transmission between these different zones, even those that were closest geographically. The evolution of societies towards the productivist world thus seems to constitute a major trend, one which was almost unstoppable and transcultural. Some authors argue that a strong environmental influence was necessary for any Neolithic revolution (such as the Oasis Theory developed by Pumpelly at the start of the 20th century [4], which was then popularized by Childe [5]). This climatic and environmental impetus may, of course, offer an explanation, but is not sufficient alone, otherwise all the birthplaces of Neolithization—or at least most of them—would have been synchronous with the global warming of the late Pleistocene, which is not the case. Other authors have argued that only a certain social and religious maturation of society could lead to the emergence of agricultural production, domestication, sedentism, and so forth [6]. Here again, the critical analysis of the data regarding this hypothesis shows that while this social and religious evolution does seem to constitute an essential prelude [7], it cannot be considered sufficient [8–10]. As often, the truth is elsewhere, undoubtedly very subtly combining natural and cultural determinism.

Whatever the case, it was in the Near East that this “Neolithic world” gradually developed for the first time in around the tenth millennium BCE before spreading rapidly, especially toward the west, mainly during the seventh millennium [11]. Over the last half a century, our knowledge has greatly evolved and scenarios have become more complex as our chronological understanding of events has improved. The initial global model—that of it spreading from east to west [12]—remains valid, but it has been enriched by diverse variants determined by a range of different parameters, at once geographical (difficulty crossing a stretch of sea for example), environmental (conditions unfavorable to the development of agriculture) and human (resistance of pre-existing populations).

With numerous sites documented both for the Neolithic and the Mesolithic of very diverse natures and in a variety of ecosystems, the Western Mediterranean constitutes an ideal test bed, particularly favorable to addressing the question of transitions (or ruptures) between the last Mesolithic hunter-gatherers and the first Neolithic farmers. Indeed, in this geographical region, which has benefitted from a long tradition of research [13], the establishment and development of the first agro-pastoral economies took place according to processes that are now relatively well understood, mainly involving colonization and the physical displacement of populations of peasant farmers (e.g. [14–17]), even if there are still under-documented areas (like the Balkans, the Aegean and the Italian Peninsula, for instance). The former hypotheses of the possible local domestication of cereals or caprines have now been definitively abandoned after it was proven that no local wild ancestors existed [18]. Although rapid on a continental scale, the westward dissemination of the technical, economic and symbolic innovations originating in the Near East nonetheless took more than three millennia, and the cultural expressions vary greatly over both time and space, incorporating adaptations, recompositions and local innovations [19]. Beyond the potential environmental constraints, one of the possible explanations for this diversity of cultural expressions in the early Neolithic in the Western Mediterranean is that of possible interactions with groups of indigenous Mesolithic hunter-gatherers. The expansion of the Neolithic clearly did not take place in a no-man’s land, but on the contrary in territories which were more or less densely occupied by indigenous groups, making the likelihood of contact between the two cultures an implicit assumption.

In recent years, the increase in absolute chronological data, in particular with radiocarbon dating [20–26], and the use of Bayesian modelling tools [18, 24, 27] have made it possible to greatly refine the chronometric framework for the Neolithic transition and to test this assumption based on objective, high-definition data. The objective of this work is thus to present the results of such an analysis carried out at the scale of the Western Mediterranean to identify the geographical areas where Neolithic farmers and Mesolithic hunter-gatherers may really have encountered each other, based on reliable chronological data.

## Latest research

The question of Mesolithic–Neolithic interactions thus constitutes a recurring aspect of studies and models of Mediterranean Neolithization. The expansion of the Neolithic from east to west is no longer seen today as a constant and continuous “wave of advance” [12] but on the contrary as an “arrhythmic” process [11]. It is no longer understood simply as the expansion of invading Neolithic groups to the detriment of indigenous populations, but more as a demic and cultural dissemination involving multiple forms of interaction between the groups. The Neolithic thus sometimes developed extremely rapid between very distant places (as illustrated by the “Leapfrog colonization” model [28–30]) while in other areas, it sometimes paused for a few centuries, before resuming a more rapid pace. These periods of latency are often put down to the Mesolithic populations being denser in these regions, thus slowing down the expansion process. This is, for example, one of the explanations proposed by Rasse [31] for the existence of the great transcontinental barrier that he identifies from Portugal to the Black Sea. He suggests that this barrier corresponds to a halt in Neolithic expansion in around 5,500 BCE. As attractive as it is however, this model fails to appraise the value of the radiocarbon datings used and lacks reflection on the methodological biases themselves, notably the artefacts induced by the calibration curve, which shows a sharp break in gradient at around this date [32].

The more widely adopted “Leapfrog colonization” model is also based on variations in the densities of Mesolithic populations. Inspired by biological invasion models, it is characterized by the selective colonization of territories that were only marginally exploited by hunter-gatherers, and the resulting genesis of Neolithic enclaves “from which further dispersal of farming proceeds by contact with and the acculturation of the local foragers” [33]. In the Western Mediterranean, the establishment of a few pioneer groups of the *Ceramica Impressa* at a few points along the coast could illustrate this type of leapfrog colonization [34]. Phenomena of cultural transfer, or even acculturation, are more difficult to demonstrate, even if some authors do not hesitate to widely generalize them. This is, for example, the case of the work of Zeder [35] on the origin and spreading of agriculture and animal husbandry in Western Europe, for whom almost the entire continent—from Gibraltar to the Seine valley and the Atlantic to the Danube—is viewed as one homogeneous area in which “indigenous foragers adopted elements of the Neolithic package”. Beyond the fact that this conclusion is asserted without real evidence, it clearly cannot be uniformly applied at such a large geographical scale.

A detailed critical examination of the archaeological contexts in which acculturation scenarios are based in fact suggests that they should be considered with the greatest caution over the entire Western Mediterranean. In Mediterranean Spain, for example, Phase C (or Cocina III) of Fortea-Pérez’s Mesolithic–Neolithic sequence was originally understood to be the result of the acculturation of indigenous Mesolithic groups [36]. But we now know that it was actually the result of stratigraphic mixing [37]. The dual model that was then developed [38] also emphasized such interactions, but here too, the reality of it is now questioned [39]. Another example is the Gaban group in northern Italy which was initially understood to be the result of a process of acculturation [40], before stratigraphic issues were once again identified [41–44]. In France, it is widely accepted that the Roucadourian, which was presented as an example of indigenous Neolithization in the 1980s [45], is a hypothesis that must be definitively rejected, as it is based only on contexts devoid of stratigraphic reliability [46, 47]. In North Africa, the hypothesis of the rooting of the Neolithic among the hunter-gatherer societies of the Capsian Mesolithic [48] must also be approached with caution in view of the low reliability of the stratigraphic contexts and available chronological data.

A critical examination of the stratigraphic and taphonomic data thus incites us to rule out most of the sites associated with acculturation scenarios, especially for the older excavations. Nonetheless, some elements of the biological and cultural data suggest that contact did indeed take place between the different groups. Thus, the great similarity between the concepts guiding lithic tool production among the Mesolithic groups of the Castelnovian and the first Neolithic groups of the *Ceramica impressa* could be an indirect testimony to contact and exchange between the two groups, and even acculturation [49]. The preservation of hunter-gatherers’ personal ornamentation styles (and likely also meanings) within emerging farming communities has also been observed in several parts of the Western Mediterranean [19]. In line with this, recent genetic studies have revealed a complex mix of Neolithic migrants originating from the Near East [50, 51] and a contribution of local Mesolithic hunter-gatherers among agriculturalist societies [52]. But, for the moment, data from archaeological contexts in the Western Mediterranean are still too scarce for us to establish global scenarios based on paleogenetics alone.

**Fig 1:**
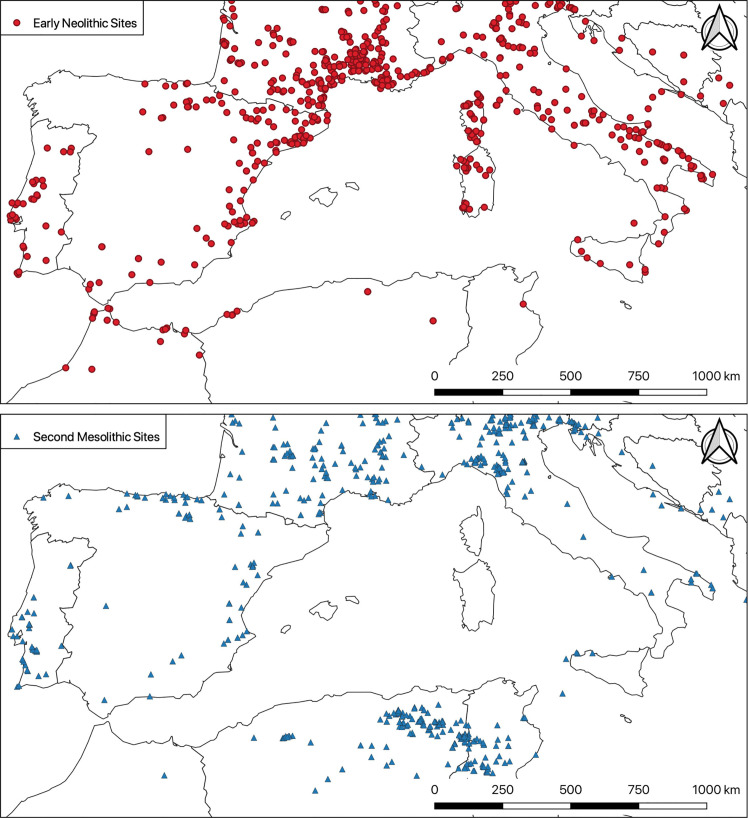
Main second Mesolithic and early Neolithic sites. Distribution maps of the main second Mesolithic (after ~ 6,500 BCE) and early Neolithic sites in the Western Mediterranean (according to the BDA database https://bda.huma-num.fr/ [56]). Countries boundaries are from Natural Earth (free vector and raster map data @ naturalearthdata.com).

Whatever the pertinence of each of these hypotheses, what they all have in common is an attempt to integrate indigenous Mesolithic groups into Neolithization scenarios. However, these groups are generally only present as a sort of backdrop, in a static and ill-defined background. Their presence is taken for granted, at best as a tacit assumption, but is hardly ever demonstrated. However, this “evident” presupposition does not always stand up to a detailed analysis of the facts, as has been shown for the Rhône valley for example [53], and even for the south of France more generally [54]. In fact, an examination of the real geographical dispersion of Mesolithic and Neolithic archaeological sites shows great irregularities. The density of sites pertaining to the Second Mesolithic is relatively high, although regionally very contrasted, notably with some relatively empty areas, such as the Tyrrhenian Islands, central Spain and Catalonia [55] for example (Fig 1). These simple maps show that while it is highly likely that contact took place between Mesolithic and Neolithic groups in various parts of the Western Mediterranean, it is not possible to generalize such a scenario at a large scale. On the contrary, we must be able to demonstrate it region by region, and the question then arises as to how to characterize such potential contact. The question of potential interactions between the last hunter-gatherers and first farmers—and more broadly that of cultural interactions between prehistoric human groups—cannot therefore be met with simple presuppositions, but on the contrary requires concrete and objective data.

## Materials and methods

**Fig 2:**
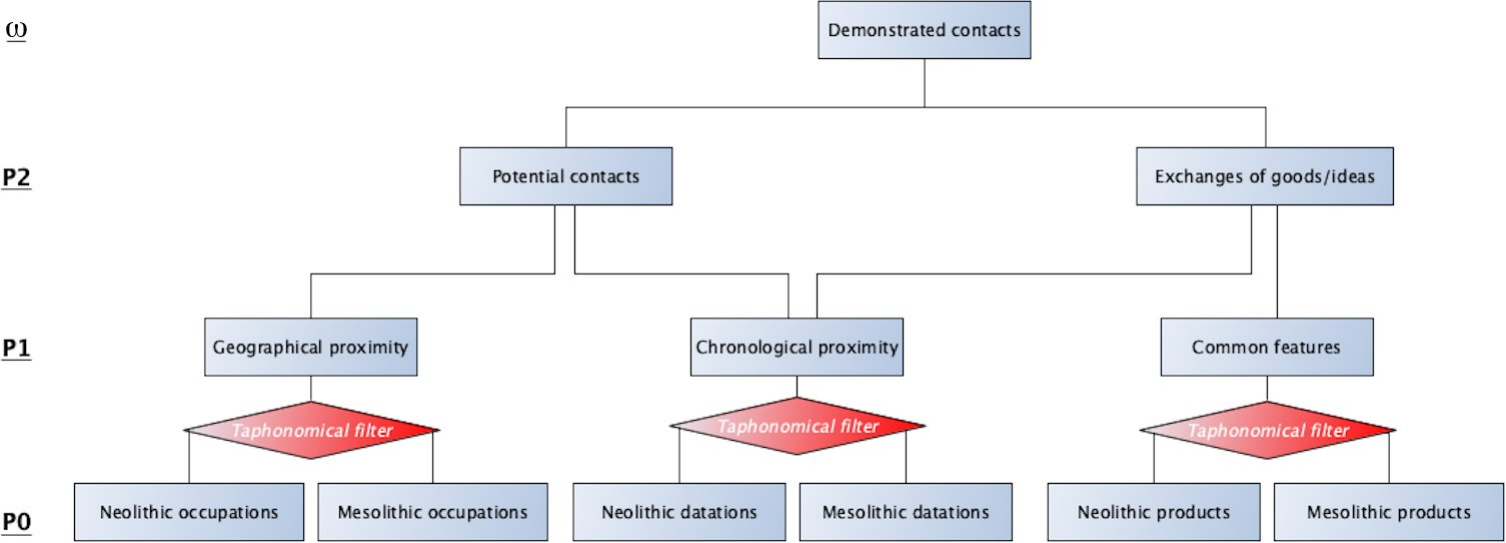
Logicistical diagram for establishing a hypothesis of contact between Mesolithic and Neolithic groups. Simplified logicistical diagram representing the necessary conditions for establishing a hypothesis (ω) of contact between prehistoric groups, in this case Mesolithic hunter-gatherers and Neolithic agro-pastoralists, on the basis of archaeological contexts and material productions. This first involves low-level propositions (P0), generally in the form of observations: Their geographical proximity must thus be demonstrated, as well as their contemporaneity (within the limits of accuracy of the absolute data) and the elements for comparison, which are generally material productions, must be analyzed in a coherent manner. Validating these first two conditions makes the hypothesis of contact possible. But it is also necessary that the remains accessible to the archaeologist, which are generally material goods, can attest that such possible contact actually took place. Finally, it is crucial to note that it is only possible to demonstrate that the groups were contemporaneous and exchanged goods or ideas once the independence of the compared samples has been demonstrated and the taphonomic processes controlled.

From a theoretical point of view, for sufficiently repeated contact to have allowed a process of integration or acculturation to take place between Mesolithic and Neolithic groups, and more broadly between two different social groups, it is necessary to demonstrate at least two unavoidable preconditions (Fig 2). It is first of all essential that there was a geographical overlap or at least a geographical proximity, so that the groups could physically meet and interact. Furthermore, in geographical areas where both Mesolithic and Neolithic sites exist, this geographical proximity only makes sense within a given chronological range. The second fundamental condition is thus to demonstrate the contemporaneity of the different groups. Only a combination of spatial and temporal proximity could make potential contact possible. Finally, contact can only really be ascertained by demonstrating an exchange of goods or ideas, of mutual influences, or sometimes gene flow.

The fundamental starting point for ascertaining possible contact is thus demonstrating geographical and temporal proximity between Mesolithic and Neolithic groups.

To achieve this, two main tools were used. The first is a free, collaborative, open-access database, which has been developed over the past thirty years (https://bda.huma-num.fr/ [56]). It records around 5,000 georeferenced prehistoric sites, including all Mesolithic and Neolithic occupations associated with the Neolithization process. For this period of the early Holocene in the Western Mediterranean, the absolute chronology data almost exclusively involves radiocarbon dating. Thus, this database also compiles all the radiocarbon dates (around 6,800 dates) for the sites considered with a critical assessment of their reliability. This assessment is based on the physical and chemical reliability of the measurements as transmitted by the laboratory, but above all on the nature of the dated samples and the link between the samples and the event they relate to. The value of the samples is based on the scale proposed by Chapman and Müller [57] which makes it possible to highlight short-life materials in particular (seeds, twigs, bones, etc.) taken from a controlled stratigraphic context. The link between the measured samples and the archaeological events to be dated is more difficult to estimate. It is based in part on the internal consistency of measurements at sites for which several dates are available, as well as their consistency with measurements documented elsewhere for the same cultural contexts. Ultimately, the individual quality of the dates gives rise to four possible values (to be rejected, doubtful, possible, reliable; Table 1), an evaluation which is of course in essence relatively subjective.

**Table 1:** Criteria for the reliability evaluation of radiocarbon dates.

			Radiocarbon event		
			Inbuilt age of the sample		
			Negligible	Medium (20–50 years)	High > 50 years
			Seeds; Animal and human unburnt bones with fully terrestrial diet	Determined wood charcoal from shrub or from a small tree branch; Short-life samples but with a large standard errors; Human bones without determined diet	Undetermined wood charcoal; Shells; Burnt animal bones; Undetermined organic matter; outliers δ13C values; Standard errors>100; Composite sample
**Link with the human event**	Good	Coherent stratigraphic sequence in a site with a well-know taphonomic history; Sample coming from an anthropogenic structure; Sample with a direct link with the occupation to be dated; Bone tool. . .	1	2	3
	Medium	Average stratigraphic coherence; Arbitrary level excavation; Low detail publication; Ancient dating series	2	3	4
	Poor	No stratigraphic context; Stratigraphic context with a poor reliability; Strong post-depositional processes; Inconsistent dataset; Major inconsistency with the cultural context	3	4	4

Summary table illustrating the different reliability criteria used to evaluate the corpus of dates. Each measurement is attributed a reliability value (from 4 = zero reliability to 1 = excellent reliability). The different sorting criteria are independent. For the “Inbuilt age of the sample”, see [58, 59].

In contrast to certain studies where the dates are taken as outright proxies without any evaluation of their archaeological context [60], we here consider that it is not the dates themselves that are significant [61] but, on the contrary, the human occupations that they relate to. An isolated dating does not mean anything in itself, it is nothing other than the measurement of a 14C event—the death of a tree or an animal, for example. What interests us is the human event to which it can be related [62]. This human event can be of variable nature and duration: an artefact, a hearth, a burial, etc. The likelihood that the 14C event actually relates to the human event that the measurement is supposed to date is by nature impossible to estimate *a priori*. It is thus precisely the work of the archaeologist to proceed with this analysis: “the association between the 14C event and the human event of interest must be examined with great care, as there must be a known link of specified magnitude connecting the two events” [ibid., p. 447]. Using a raw date as a supposedly reliable estimate of anything other than a simple 14C event is therefore a methodological error both from the point of view of the dating methodology and the archaeological discipline itself.

Furthermore, dated human events can sometimes be related to each other, for example within the same occupation of a site, that is to say at a chronologically consistent moment of “habitation”, without making any assumptions about its duration, which could range from a few days to a few years. The link between the single dated human event and one of the human occupations of a site can be objectively demonstrated from an archaeological perspective and the coherence of the occupation can also be estimated on the basis of explicit and demonstrative criteria: for example, the pertinence of the excavation method used, the consistency of the stratigraphy and the archaeological material identified or that of the absolute dates available, the spatial distribution of the remains, the results of sedimentological and taphonomic analyzes, etc. And it is of course these occupations that make it possible to draw links between material productions, biological remains, human activities… in short, to reconstruct a moment in prehistoric life.

These occupations can be situated in time by one or more absolute measurements, their stratigraphic position, the artefacts they contain, and so forth. It is only by considering all these data together that we can obtain a relatively precise and reliable chronological range for each of the occupations of a site, this work being carried out in particular by means of Bayesian modelling. The increase in the number of dates in recent decades has made the generalization of Bayesian modelling all the more relevant in refining chronological frameworks. This is particularly the case when they can be produced for a single site, as it makes it possible to overcome the relatively subjective estimate of the intrinsic value of each of the dates. All these dates are then integrated within a model which is based on the general conformity of the whole set of data, according to *a priori* constraints reflecting the stratigraphic observations of the site. These models are thus an effective way of objectifying chronological data in relation to archaeological observations.

**Fig 3:**
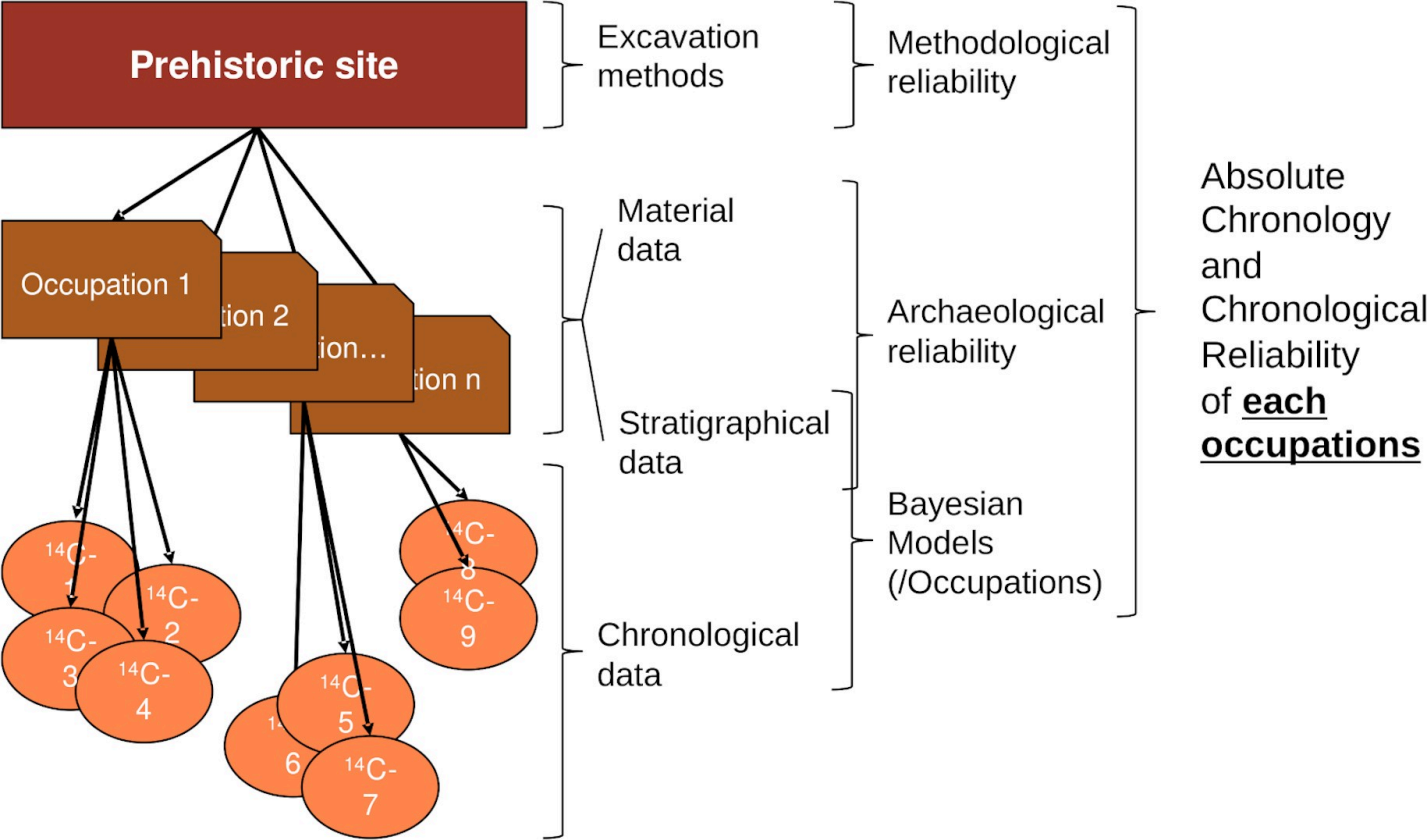
Method to situate in time the occupations of a site. Methodological principles allowing us to situate the occupation(s) of a site over time and to estimate the reliability of the data.

In the BDA database, in which all dates have been calibrated with the latest available curve [32, 63, 64], the chronological evaluations of the occupations are thus based whenever possible on the results of Bayesian models or on an estimation of the reliability of the available measurements when they are isolated. This relatively precise and reliable method for dating occupations is the second tool on which this work is based (Fig 3).

The chronological uncertainty of each of these occupations is assessed using a three-value scale (Table 2).

**Table 2:** Criteria for assessing the reliability of prehistoric occupations.

General Reliability	Stratigraphy	Excavation methods	Absolute datings	Matériel productions	Regional consistency	Publication
**1 (good)**	++	++	Several dates consistent with each other or one into a reliable model	Internal consistency of the material data	++	Detailed monograph
**2 (moderate)**	+	+	One single date or several possible ones	Not enough information or dubious ones	+	Partial papers
**3 (low)**	- or?	- or?	None or no accurate ones	Unknown or mixed material data	- or?	No detailed publication

The occupations with a reliability value of 1 are thus situated over time by several consistent measurements, which are sometimes integrated into a Bayesian model and which come from well-excavated sites with a controlled stratigraphy. These models are used to define the highest probable *a posteriori* start and end dates for each occupation. Data is also available on the archaeological remains related to the date, especially in the form of detailed publications which make it possible to estimate the conformity of the data as a whole within the site itself but also at a regional scale.

Occupations with a reliability value of 2 are more uncertain, either due to a lack of precision in the chronological sequence, a less well-controlled stratigraphy, or a lack of detail in the published material. The time range for these occupations are those defined by the radiocarbon dates themselves, calibrated at 95% probability.

Finally, occupations with a reliability value of 3 are generally undated, or poorly dated, and cannot be precisely and objectively located over time. Their chronological situation thus remains very vague, that generally accepted for the cultural facies they belong to. These occupations, which are the most numerous, cannot be used to demonstrate or disprove the existence of possible contact between Mesolithic and Neolithic groups.

This method allows us to obtain high-resolution chronological mapping for Mesolithic and Neolithic occupations.

## Results

**Fig 4:**
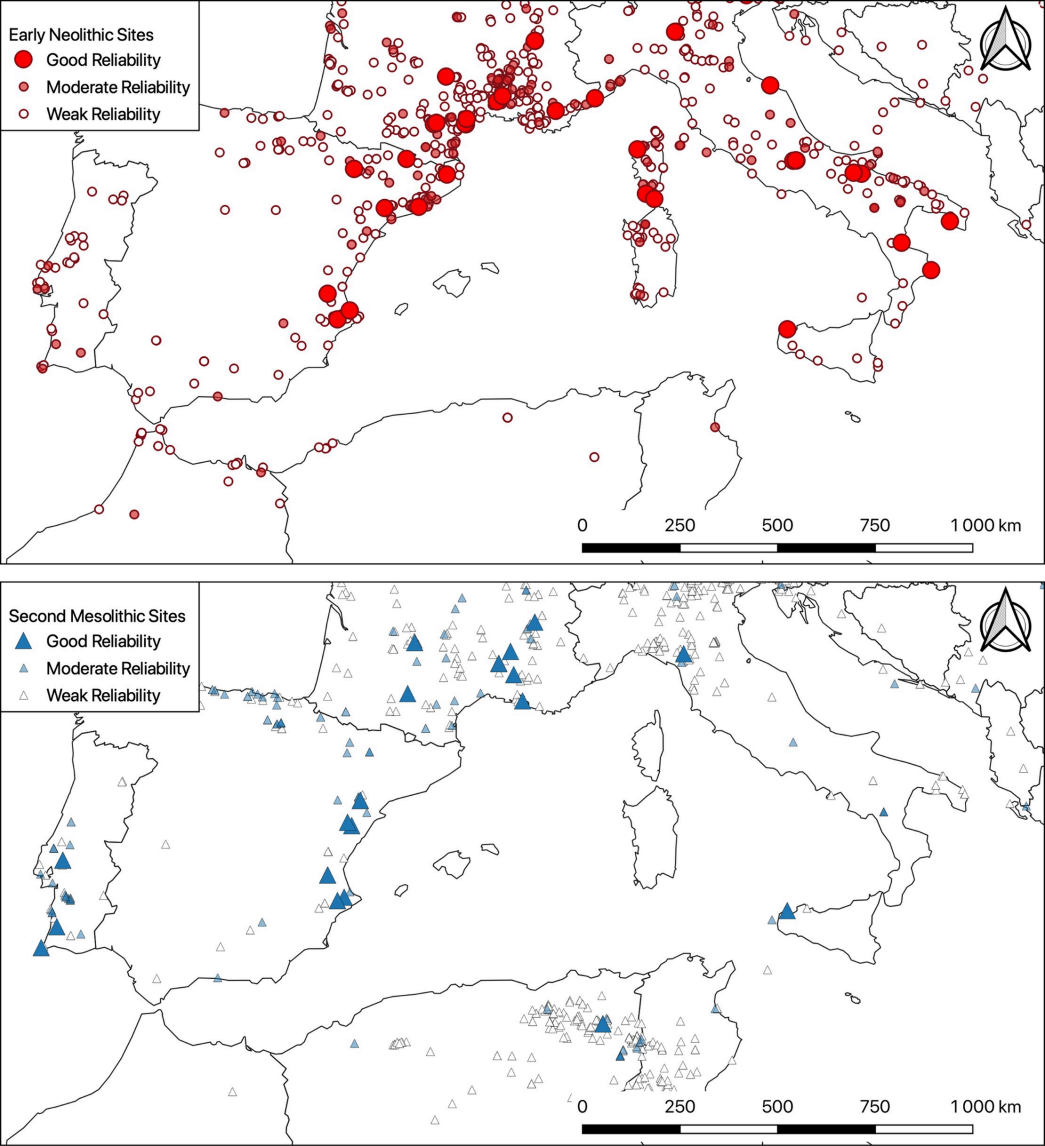
Distribution maps of the main second Mesolithic (after ~6,500 BCE) and early Neolithic occupations in the Western Mediterranean classified according to their reliability. Countries boundaries are from Natural Earth (free vector and raster map data @ naturalearthdata.com).

Mapping occupations according to this scale of values strongly alters our perception of the geographical dispersion of the available data (Fig 4). For the Second Mesolithic in particular, several wide geographical areas are devoid of reliable data. In general, well-dated and well-documented occupations are rare. The situation is a little better for Early Neolithic sites, but this is mainly due to the fact that there are more of them. However, these maps are not satisfactory either, because they aggregate sites that are sometimes centuries apart, and it is therefore imperative that we refine our chronological scale.

There are several possible methods to achieve this. We could plot the respective distribution of the sites in the form of isochrone curves. This is the option that was used in previous studies already cited [31, 54]. However, this mode of representation has the drawback of creating artificial boundaries that are sometimes too clear-cut compared to reality: isolated sites particularly (statistical outliers, which can nonetheless be very significant from an archaeological point of view) tend to be lost with this method. Likewise, representations in the form of areas of dispersion simplify the situation even more and only really make sense in very global approaches [11]. However, in terms of identifying zones of interaction between Mesolithic and Neolithic groups, all these types of cartography are problematic. Indeed, the Neolithization process always seems to be presented as a kind of “wave of advance”, even if it no longer appears at all regular and continuous, approached exclusively through the filter of the Neolithic groups themselves, who overwhelm a world of Mesolithic hunters—a world that is conspicuously absent on all these maps. It seemed to us that the most relevant approach here was to try and stay as close as possible to the raw data in the form of two-point clouds grouped together in 100-year intervals. All the Mesolithic and Neolithic occupations can thus be represented in their exact geographical position and according to a relatively precise chronology. The choice of a defined time range, in this case 100 years, of course induces the potential effects of artificial boundaries. However, it would be somewhat illusory to try to achieve a finer chronological resolution; Bayesian calibration and modelling software certainly provide results down to the year, but on the basis of initial measurements with broad standard deviations of several decades. A 100-year interval thus seems most pertinent for now.

The maps thus produced were concatenated in the form of videos (S1 and S2 Videos) and a series of maps (S1 and S2 Files). The period considered extended from 6800 ± 50 BCE to around 4800 ± 50 BCE, thus making it possible to cover the entire duration of the Neolithization process in the Western Mediterranean. Two versions of these data have been produced, one of which includes the least reliable, value 3 occupations. The visuals that include these value 3 occupations are interesting but more complex to read because many occupations, which have been poorly or even very poorly dated, remain visible for very long periods, as they could only be very roughly situated in time. Their inclusion thus leads to a blurring that affects the readability of the most reliable data. By restricting these visuals to value 1 and 2 occupations, the image changes significantly. In particular, entire areas appear to have been totally uninhabited before the arrival of Neolithic groups, such as the main part of the Italian peninsula, the whole of the Spanish hinterland (“Meseta”) and nearly all the main Mediterranean islands. In other areas, Mesolithic occupations persisted for longer, such as in northern Italy, northern Spain, southern Portugal, north-eastern Algeria, south-western Tunisia, and so forth. We cannot of course interpret these population densities as perfect reflections of real prehistoric occupation. A range of biases could have generated all or part of these variations, be it the research history, taphonomic filters, or other factors. Nonetheless, this is the real and objective data that we dispose of, and it is therefore on this basis that we must work, considering that it nevertheless reflects at least a part of the prehistoric reality. These highly-variably occupation densities at a regional level imply the existence of highly variable dynamics in terms of interactions between Mesolithic and Neolithic groups. We can identify four examples here illustrating four different possible scenarios.

**Fig 5:**
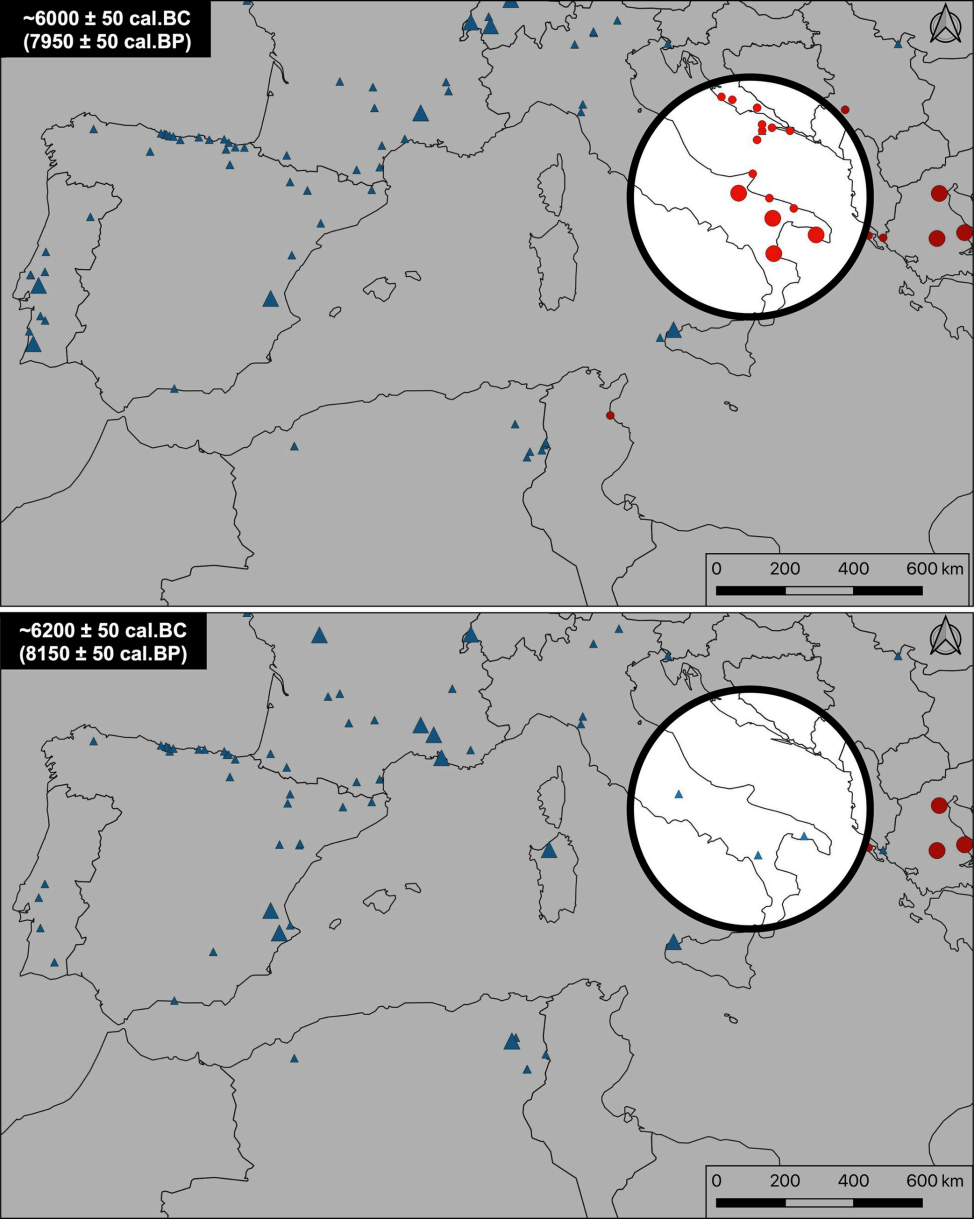
Example of a “no man’s land” scenario. Example of a “no man’s land” scenario: The last Mesolithic occupations disappeared nearly 200 years before the first Neolithic settlements, precluding any contact between the two groups. Red dots: Neolithic occupations; blue triangles: Mesolithic occupations. Countries boundaries are from Natural Earth (free vector and raster map data @ naturalearthdata.com).

In southern Italy and the Adriatic coast, for example, there is a gap of more than 200 years between the last occupations of the Mesolithic and the first Neolithic manifestations (Fig 5). The last dated Mesolithic occupations seem to be situated around 6,200 BCE (Continenza c.24–23, Terragne or Latronico 3, knowing that the dates of all these sites remain unreliable), following which they all disappear relatively abruptly. The Early Neolithic period in turn appears quite suddenly around 6,000–5,800 BCE (Trasano, Torre Sabea, Favella, Coppa Nevigata) and seems to have spread very rapidly. This significant chronological gap of two centuries suggests that Neolithic groups may have been able to establish themselves in territories that were almost deserted by the last hunter-gatherers in a kind of “no man’s land” scenario.

**Fig 6:**
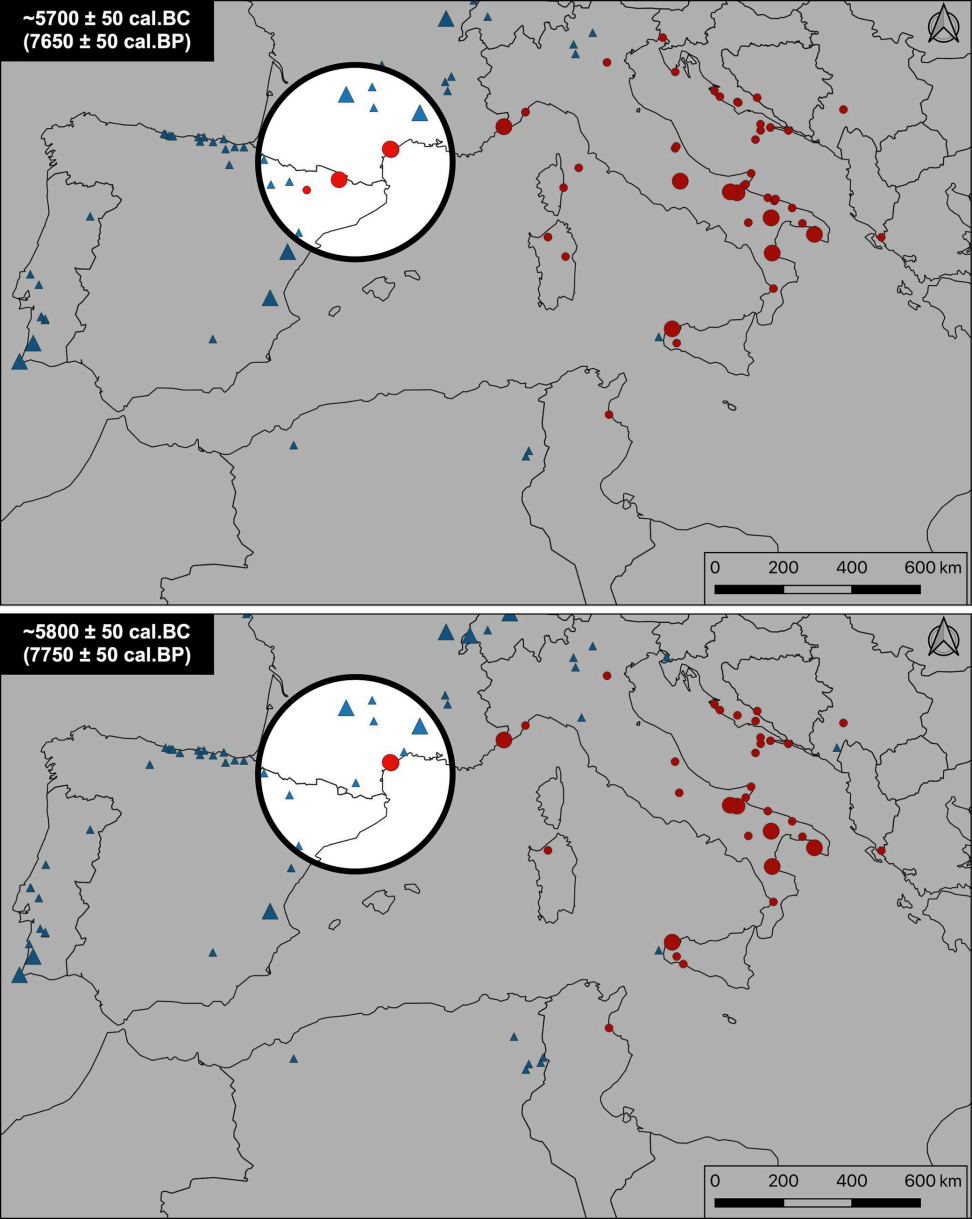
Example of a “musical chairs” scenario. Example of a “musical chairs” scenario: The last Mesolithic occupations disappeared less than 100 years before the first Neolithic settlements, or coexisted more than 50 miles (ca. 80 km) from each other, making the hypothesis of regular contact unlikely and suggesting rapid succession. Red dots: Neolithic occupations; blue triangles: Mesolithic occupations. Countries boundaries are from Natural Earth (free vector and raster map data @ naturalearthdata.com).

In the western Languedoc and northern Catalonia (Fig 6), the situation is different, and we can again generally observe a strict succession between the last Mesolithic groups and the first Neolithic groups: there are no proven cases in the currently available data of the simultaneous presence of the two populations in the same territory [54]. There is, however, also no significant (i.e. >100 years) gap between the two, suggesting a rapid reoccupation of progressively abandoned Mesolithic territories, in a scenario that can be likened to the game of “musical chairs.”

In Liguria, the situation seems to be different again, even if the absence of any dated Mesolithic site does not allow a very fine analysis. The two groups seem to have coexisted in the same territory, but occupying and exploiting different biotopes [65, 66]. There is no evidence of direct, permanent contact between them, which has led our colleagues to describe this model as an “avoidance strategy” [67]. A relatively similar scenario can be identified in Asturias and Cantabria where the two groups seem to have coexisted for a few decades at the start of the fifth millennium BCE [68].

Finally, it is sometimes possible to demonstrate the real coexistence of the two groups and exchanges and interactions between them that were sufficiently long and repeated to have allowed for a real acculturation process. However, these cases remain the exception. One example is the cave of Gardon, in the upper Rhône valley, where a Neolithic occupation was succeeded by a Mesolithic group at the same site [69]. The stratigraphic interweaving suggests that these different groups shared the same territories at the same time. Exchanges and interactions can also be proven by the persistence of Mesolithic traditions in later Neolithic groups, as with the Saint-Uze group at the beginning of the 5th millennium BCE [70]. These late resurgences are not without echo in the recent results of paleogenetic analyses, which also demonstrate the reappearance of Mesolithic haplogroups during the 5th millennium BCE [71].

## Discussion

From these few examples, we can identify at least four different types of relationship between Mesolithic and Neolithic groups, which can be divided into two main categories—colonization without any interaction versus colonization with interactions that may have led to acculturation (Table 3 and [15]). In the first two scenarios (“no man’s land” and “musical chairs”), the two groups did not frequent the same territory, in this case a geographical zone around 50 miles (ca. 80 km) in diameter. This geographical distance between the two groups implies the impossibility of repeated contact between them. If we further note the existence of a real chronological hiatus between them (estimated here at more than 100 years), this contact can be considered impossible, even on an occasional basis. When the most recent group arrived—in this case farmers—the territory had long been abandoned by the earlier Mesolithic group (“no man’s land” scenario). It thus involved a strict colonization process. If, on the other hand, the chronological interval between the two was low (i.e. <100 years), farmers would have settled in areas only recently abandoned by Mesolithic hunters in a kind of “musical chairs” scenario. In such cases, the Mesolithic hunters appear to have been more or less driven away by Neolithic expansion, reflecting a colonization process linked to demographic expansion.

**Table 3:** Main characteristics of the different models of potential contact.

Scenario	Territorial coexistence	Chronological gap	Repeated contacts	Anthropological process
	(i.e.<50 miles [ca. 80 km])			
		(i.e.≥100 y)		
**“No man’s land”**	_	X	_	Colonization
**“Musical chairs”**	_	_	_	Demographic growth
**“Avoidance strategy”**	X	_	_	Imposed acculturation
**“Cultural mixing”**	X	_	X	Asked acculturation

The former two correspond to processes of colonization without interaction (“no man’s land” and “musical chairs” scenarios), the latter two to processes of interaction and potential acculturation (“avoidance strategy” and “cultural mixing” scenarios). The anthropological process indicated refers to [15].

The two social groups may also have coexisted within the same territory, that is to say that Mesolithic and Neolithic occupations attested within 50 miles (ca. 80 km) of each other within a 100-year interval. Contact between them can thus be considered possible. Contact did not necessarily take place repeatedly however, if at all. The two groups may appear to have avoided each other (“avoidance strategy” scenario). These situations can also be related to the hypothesis of the self-exclusion of the last hunter-gatherers in the face of the arrival of Neolithic pioneers, driving them to leave their territory and to their gradual disappearance [72]. Conversely, they may have come into regular contact (“cultural mixing” scenario). In the context of Neolithization, these scenarios would have led to processes of acculturation, which were imposed upon them in the former case and were embraced by them in the latter [15].

**Fig 7:**
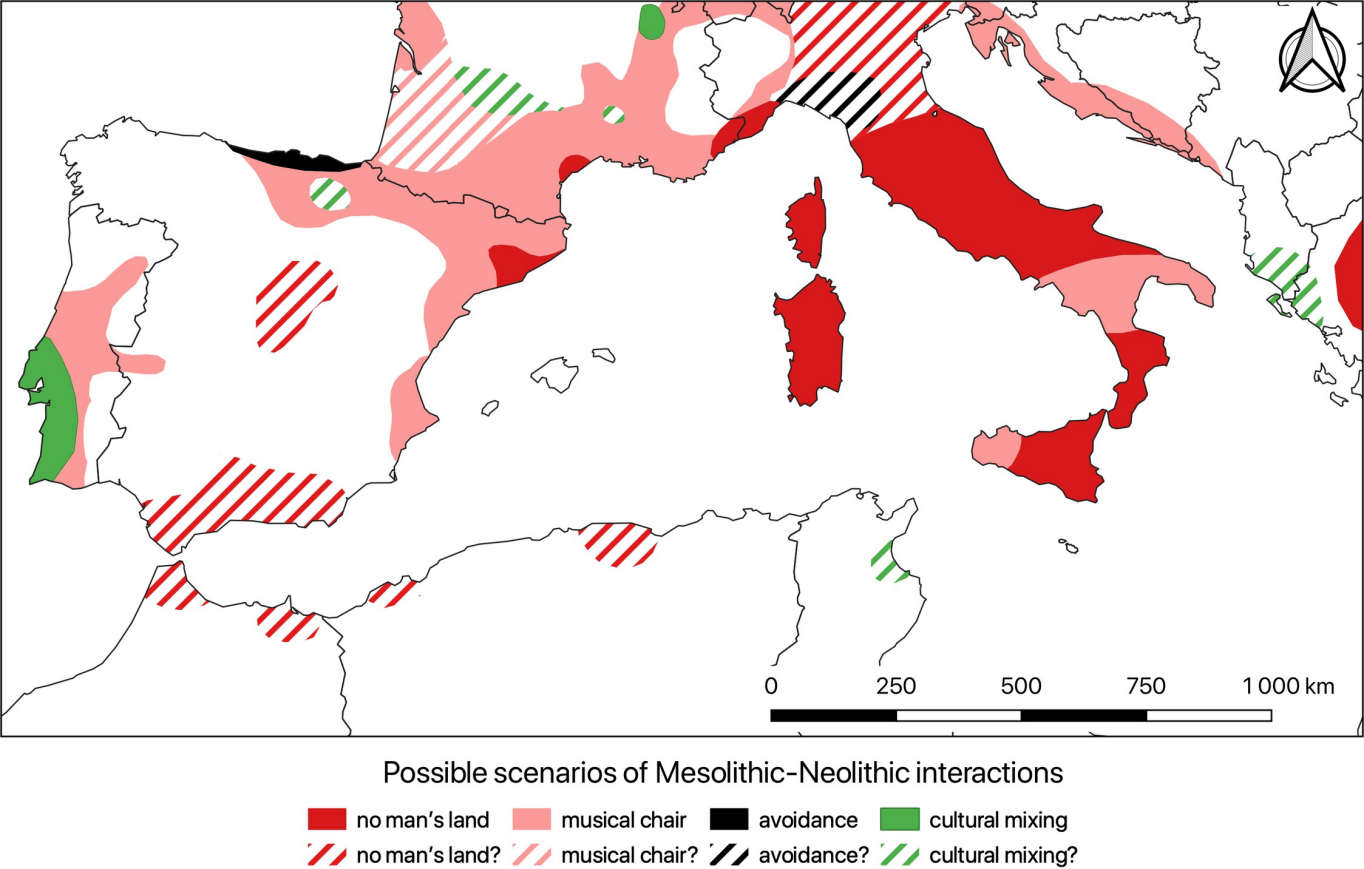
Different possible interaction scenarios between Mesolithic and Neolithic. Schematic map of the different possible interaction scenarios between Mesolithic and Neolithic groups during the Neolithization process in the Western Mediterranean, according to the currently available data. Countries boundaries are from Natural Earth (free vector and raster map data @ naturalearthdata.com).

If we try to map these different processes on the scale of the Western Mediterranean (Fig 7), we can see that the two main scenarios are those related to the colonization processes without any interaction. Thus, most of the regions involve scenarios of succession without contact, as in the “no man’s land” and “musical chair” scenarios. Possible or proven cases of coexistence are rare and limited to small geographical areas. Sometimes, as in the Maghreb [26], dense populations of hunter-gatherers somehow seem to resist the waves of Neolithization for a few decades or centuries. Elsewhere, Mesolithic and Neolithic groups seem to avoid each other, like perhaps in Asturias. In Portugal, the Upper Rhone Valley and most likely Albania, the coexistence of different groups is conceivable and cultural mixing possible.

Of course, these scenarios and interpretations are heavily dependent on the available data. In some regions, the scarcity or even absence of sites must be a concern. These voids may be linked to taphonomic bias (rising sea level, burial under thick alluvial deposits, modern agricultural practices or land management, etc.), to more or less intense regional research dynamics, or to real hiatuses of occupation. Likewise, most of the sites have not been dated in absolute terms, which again greatly limits the finesse of our analyses. All these biases are inherent to all large-scale work (for example [73, 74]), which remains highly dependent on the partiality of the data. Our model is based on the most reliable data available and will be refined and specified in the coming years.

Even if this map is mainly indicative, it highlights the complexity of the Neolithization process, showing that it would be futile to try to summarize it with a single scenario. The process appears to have been fundamentally non-linear and anything but binary. Only a regional scale can be relevant in understanding this process in its full complexity and above all its historical reality.

This map also highlights the severe lack of reliable chronological data in too many regions, creating great uncertainty. Obtaining reliable chronological and stratigraphic data as well as publishing detailed monographs is crucial for us to be able to go any further.

## Conclusion

This study demonstrates the full complexity of the Neolithization process in the Western Mediterranean and our great dependence on the quality of the available data. Precisely locating the prehistoric occupations in time and space is the most reliable way of beginning to achieve this. Despite the refining of radiocarbon measurements and the increasingly systematic use of Bayesian models however, the chronological resolution that we are able to achieve remains relatively imprecise, rarely involving less than a century. It is thus difficult to attest the real contemporaneity of two occupations which were close in time and in space. As a result, the identification of acculturation processes can only really take place *a posteriori*, once completed, when they have given rise to entities specific enough for us to be able to identify them. However, their existence tells us that these processes did well take place and that it is, therefore, possible to try to identify them. It is in these particular geographical areas that we must focus in order to document these complex processes in detail.

## Supporting information

S1 Video Animated video of the position of Mesolithic (blue triangles) and Neolithic (red circles) sites in the Western Mediterranean by 100-year time interval between 6800 ± 50 BCE and 4800 ± 50 BCE. The white symbols relate to occupations with a reliability value of 3. The small full-colored symbols are reliability 2, and the large ones are reliability 1. Countries boundaries are from Natural Earth (free vector and raster map data @ naturalearthdata.com).
(MP4)

S2 Video Animated video of the position of the most reliable (value 1 and 2) Mesolithic (blue triangles) and Neolithic (red circles) occupations in the Western Mediterranean by 100-year interval between 6800 ± 50 BCE and 4800 ± 50 BCE. The small full-colored symbols relate to occupations with a reliability value of 2, the large ones are reliability 1. Countries boundaries are from Natural Earth (free vector and raster map data @ naturalearthdata.com).
(MP4)

S1 File Maps of the position of Mesolithic (blue triangles) and Neolithic (red circle) sites in the Western Mediterranean by 100-year interval between 6800 ± 50 BCE and 4800 ± 50 BCE. The white symbols relate to occupations with a reliability value of 3. The small full-colored symbols are reliability 2, and the large ones are reliability 1. Countries boundaries are from Natural Earth (free vector and raster map data @ naturalearthdata.com).
(ZIP)

S2 File Maps of the positions of the most reliable (value 1 and 2) Mesolithic (blue triangles) and Neolithic (red circles) occupations in the western Mediterranean by 100-year interval between 6800 ± 50 BCE and 4800 ± 50 BCE. The small full colored symbols relate to occupations with a reliability value of 2, the large ones are reliability 1. Countries boundaries are from Natural Earth (free vector and raster map data @ naturalearthdata.com).
(ZIP)

S1 Appendix List of the sites and occupations used with their geographical coordinates (WGS84), their cultural attribution and the chronological range used (.csv format, separator: Tabulation). This list is extracted from the free and online database BDA (https://bda.huma-num.fr/ and DOI:10.34847/nkl.dde9fnm8) on which more detailed information is available.
(CSV)
