# Supplementary figures and images for: Potential interactions between Mesolithic hunter-gatherers and Neolithic farmers in the Western Mediterranean: The geochronological data revisited
Source: PLoS One. 2021 Mar 3;16(3):e0246964. doi: 10.1371/journal.pone.0246964 (PMC7928471; doi:10.1371/journal.pone.0246964)

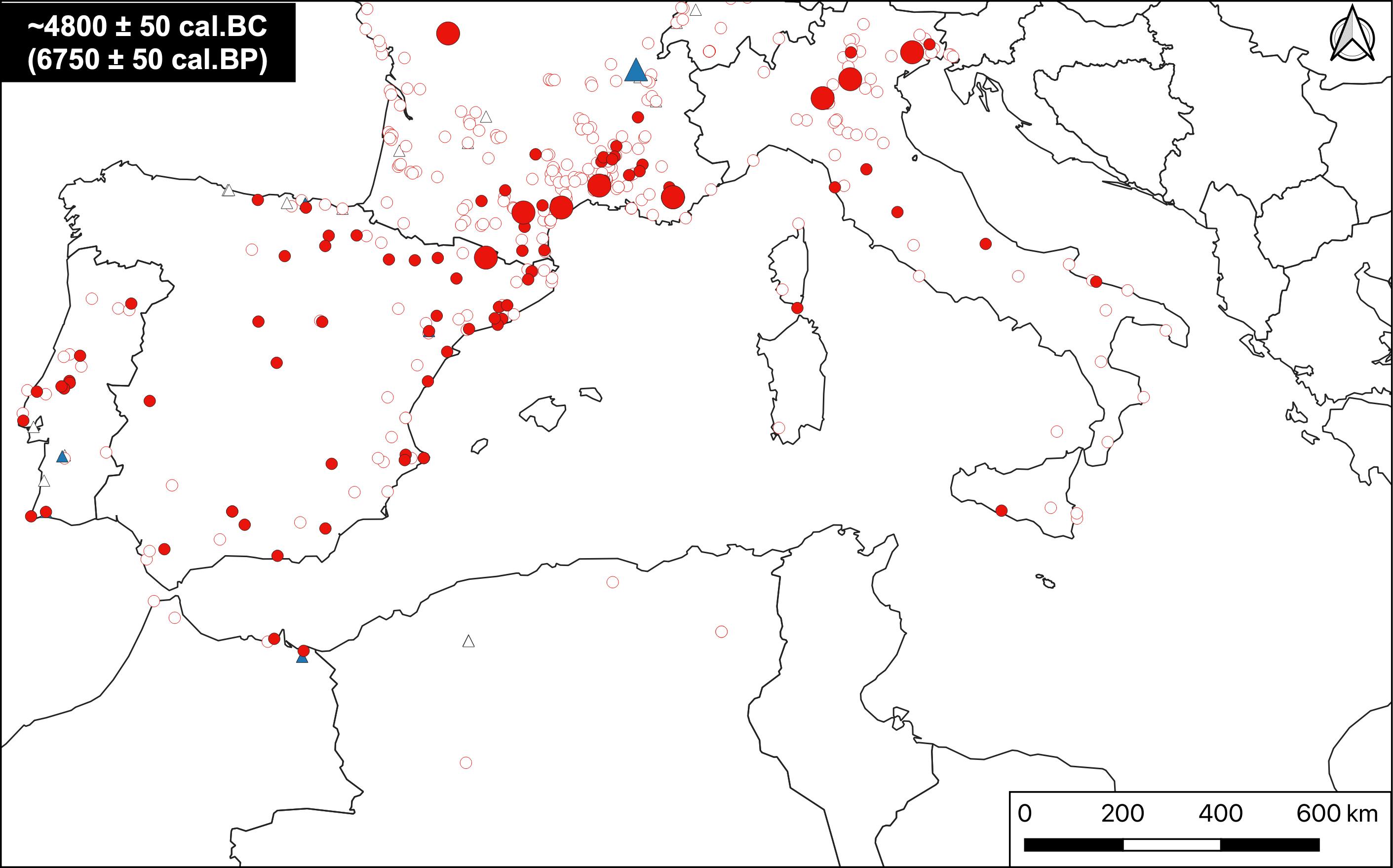

Supplement: S1 File — The white symbols relate to occupations with a reliability value of 3. The small full-colored symbols are reliability 2, and the large ones are reliability 1. Countries boundaries are from Natural Earth (free vector and raster map data @ naturalearthdata.com). (ZIP) [file pone.0246964.s003.zip › 4800-rel1_2_3.jpg]

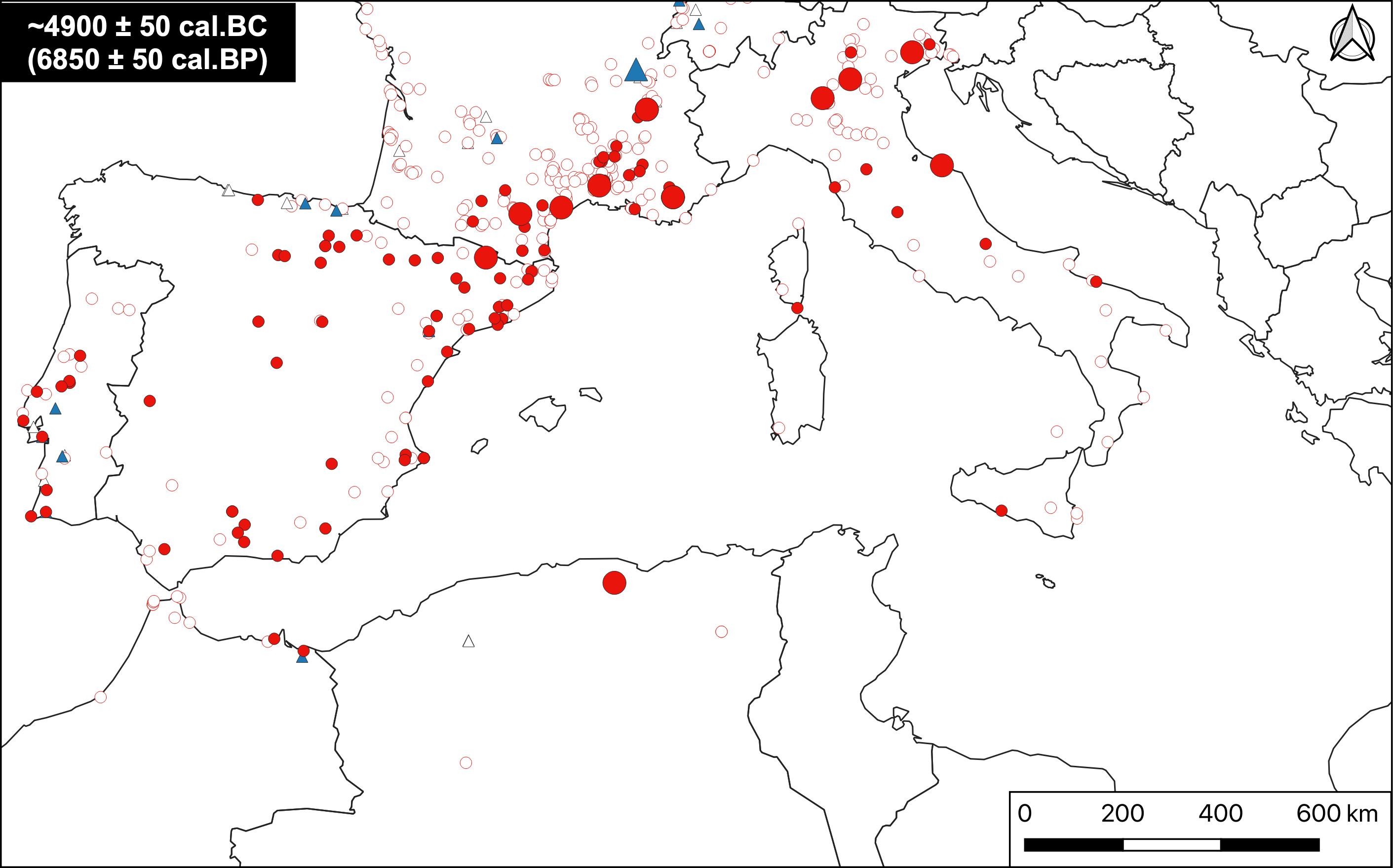

Supplement: S1 File — The white symbols relate to occupations with a reliability value of 3. The small full-colored symbols are reliability 2, and the large ones are reliability 1. Countries boundaries are from Natural Earth (free vector and raster map data @ naturalearthdata.com). (ZIP) [file pone.0246964.s003.zip › 4900-rel1_2_3.jpg]

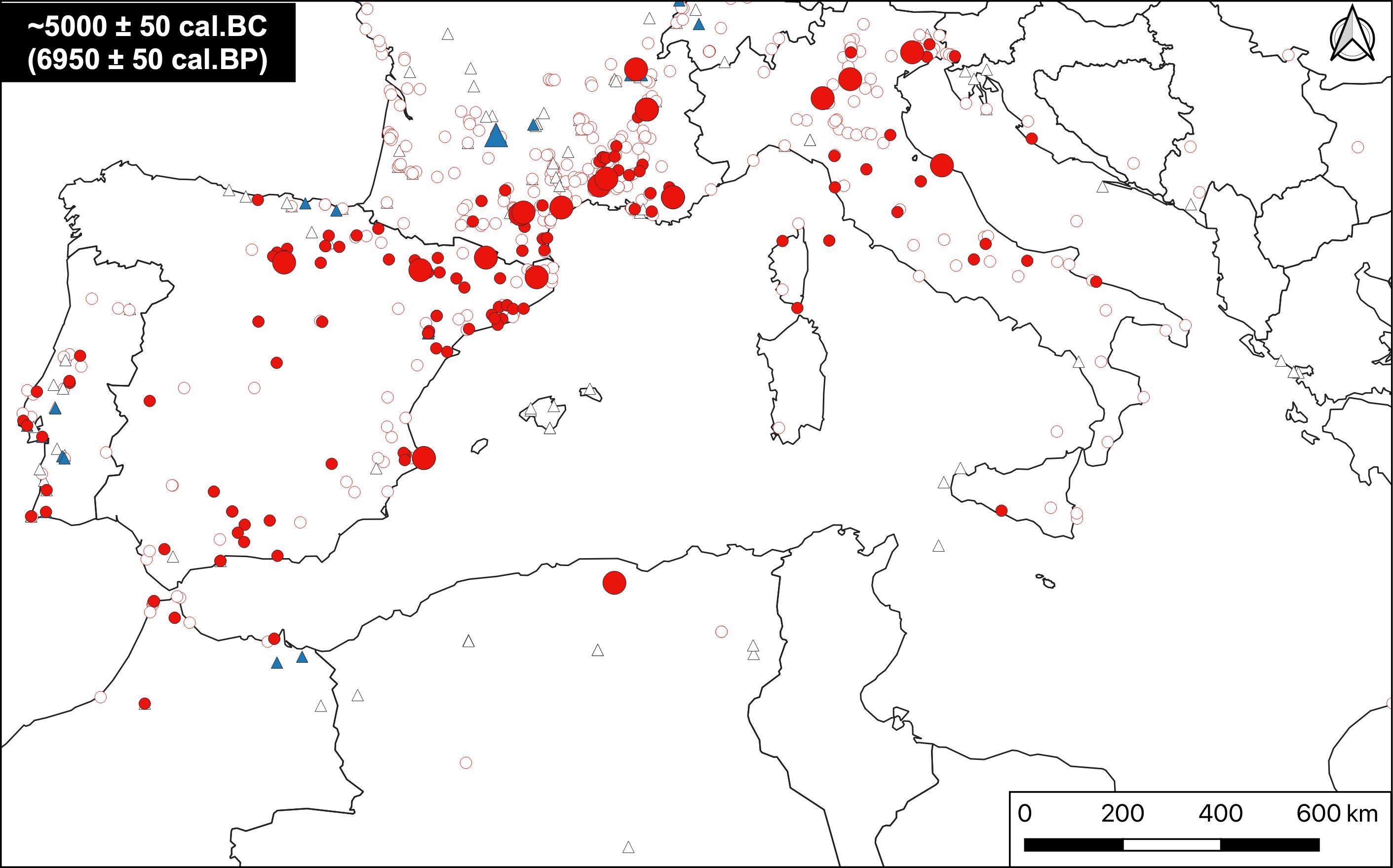

Supplement: S1 File — The white symbols relate to occupations with a reliability value of 3. The small full-colored symbols are reliability 2, and the large ones are reliability 1. Countries boundaries are from Natural Earth (free vector and raster map data @ naturalearthdata.com). (ZIP) [file pone.0246964.s003.zip › 5000-rel1_2_3.jpg]

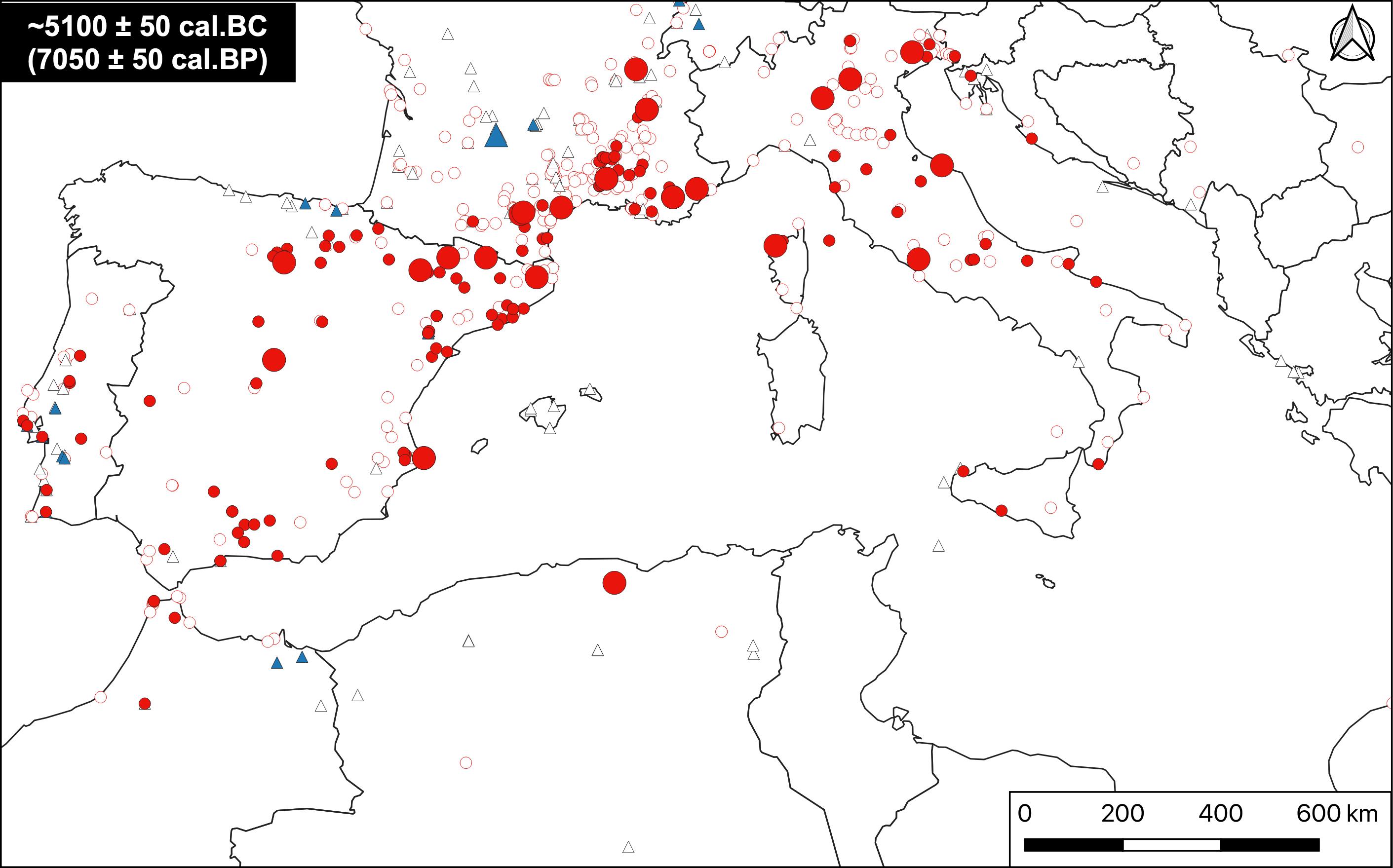

Supplement: S1 File — The white symbols relate to occupations with a reliability value of 3. The small full-colored symbols are reliability 2, and the large ones are reliability 1. Countries boundaries are from Natural Earth (free vector and raster map data @ naturalearthdata.com). (ZIP) [file pone.0246964.s003.zip › 5100-rel1_2_3.jpg]

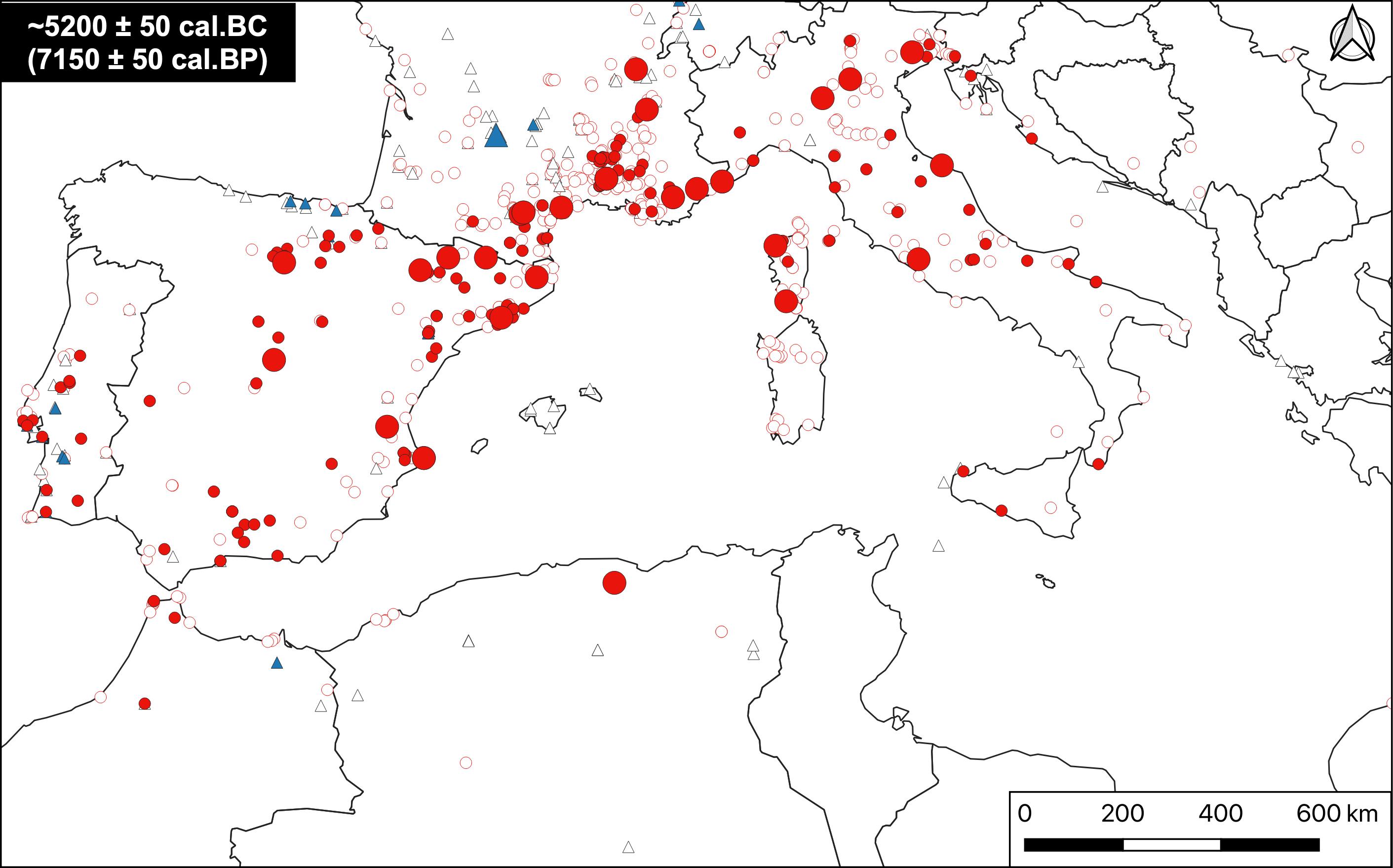

Supplement: S1 File — The white symbols relate to occupations with a reliability value of 3. The small full-colored symbols are reliability 2, and the large ones are reliability 1. Countries boundaries are from Natural Earth (free vector and raster map data @ naturalearthdata.com). (ZIP) [file pone.0246964.s003.zip › 5200-rel1_2_3.jpg]

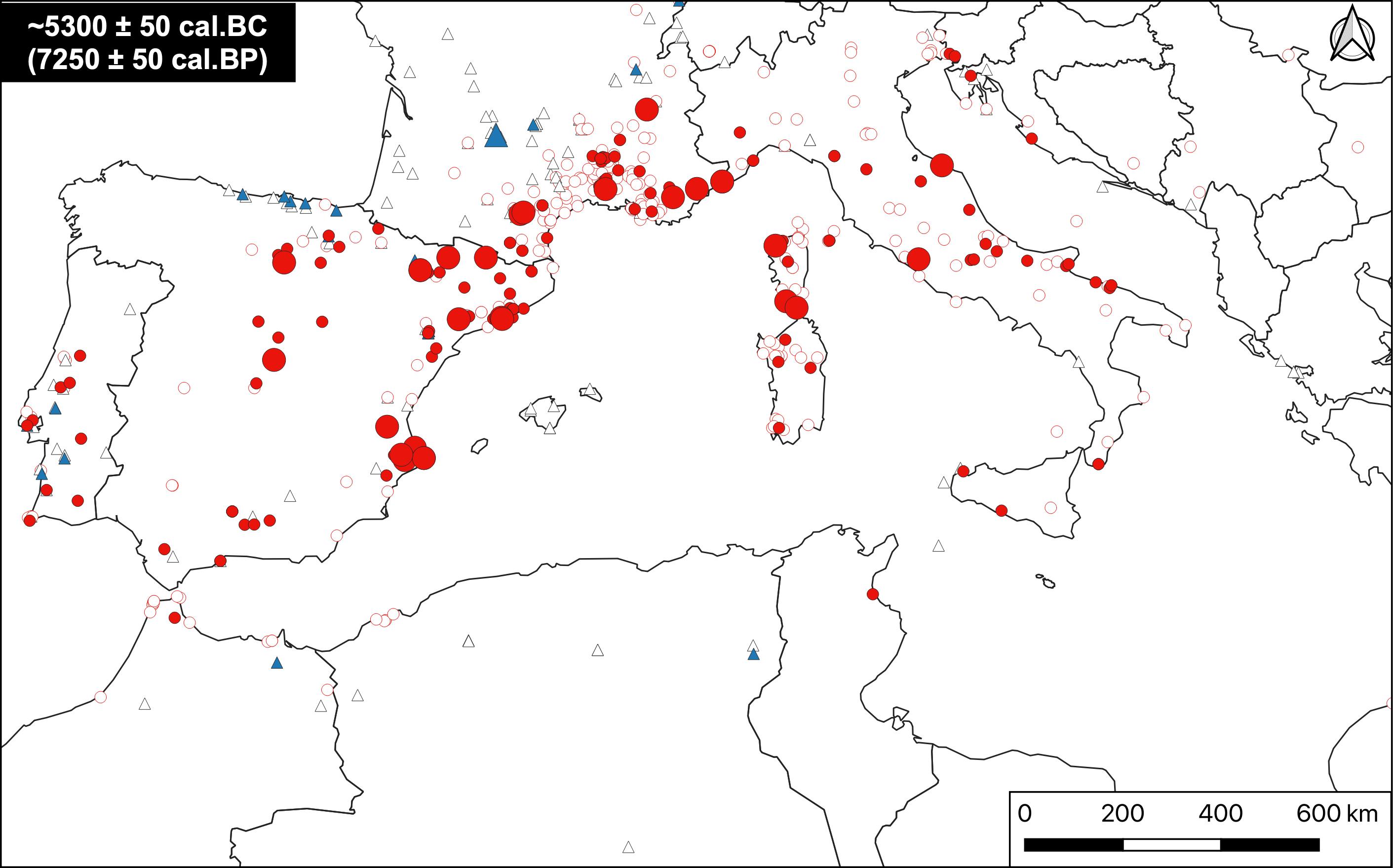

Supplement: S1 File — The white symbols relate to occupations with a reliability value of 3. The small full-colored symbols are reliability 2, and the large ones are reliability 1. Countries boundaries are from Natural Earth (free vector and raster map data @ naturalearthdata.com). (ZIP) [file pone.0246964.s003.zip › 5300-rel1_2_3.jpg]

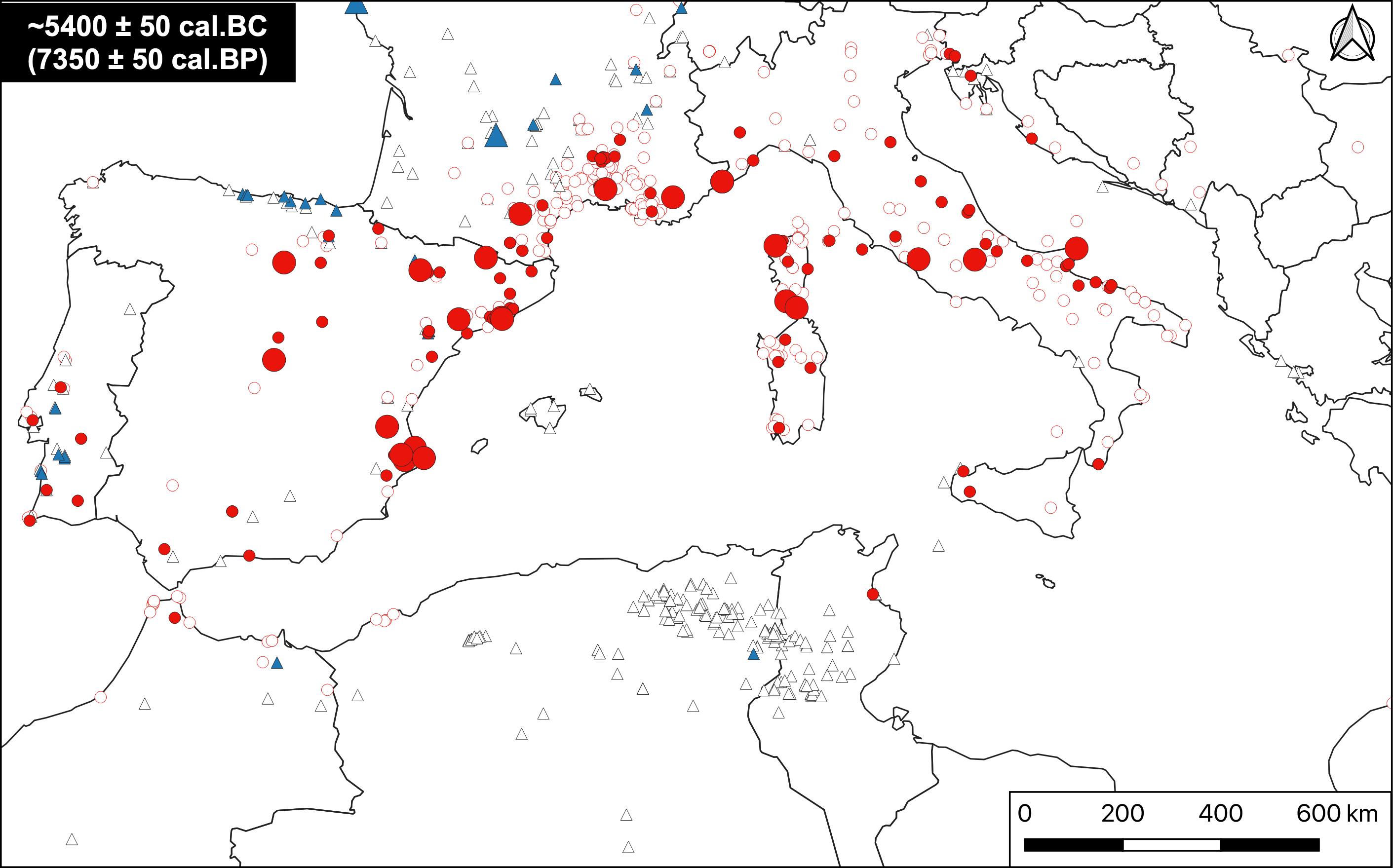

Supplement: S1 File — The white symbols relate to occupations with a reliability value of 3. The small full-colored symbols are reliability 2, and the large ones are reliability 1. Countries boundaries are from Natural Earth (free vector and raster map data @ naturalearthdata.com). (ZIP) [file pone.0246964.s003.zip › 5400-rel1_2_3.jpg]

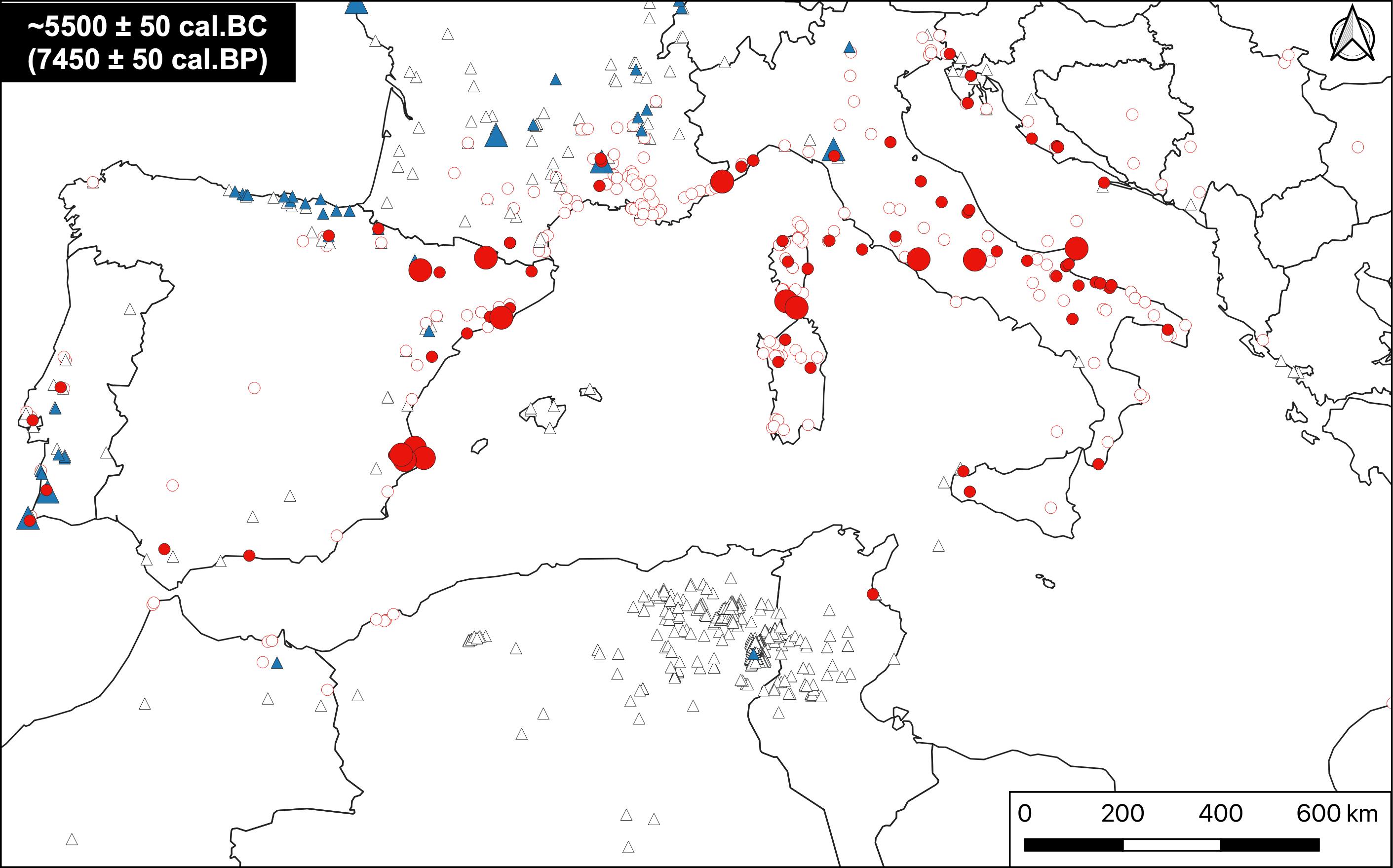

Supplement: S1 File — The white symbols relate to occupations with a reliability value of 3. The small full-colored symbols are reliability 2, and the large ones are reliability 1. Countries boundaries are from Natural Earth (free vector and raster map data @ naturalearthdata.com). (ZIP) [file pone.0246964.s003.zip › 5500-rel1_2_3.jpg]

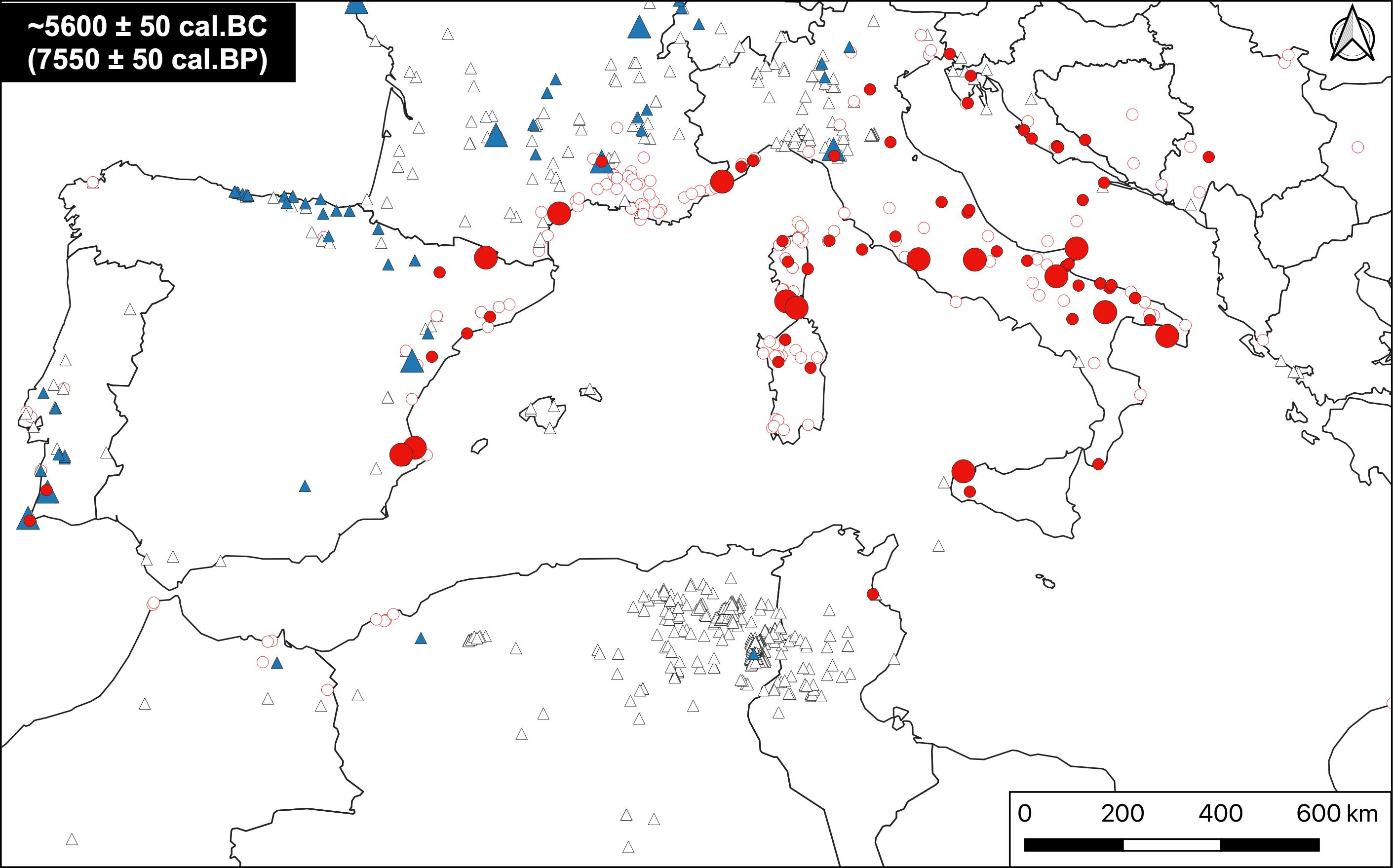

Supplement: S1 File — The white symbols relate to occupations with a reliability value of 3. The small full-colored symbols are reliability 2, and the large ones are reliability 1. Countries boundaries are from Natural Earth (free vector and raster map data @ naturalearthdata.com). (ZIP) [file pone.0246964.s003.zip › 5600-rel1_2_3.jpg]

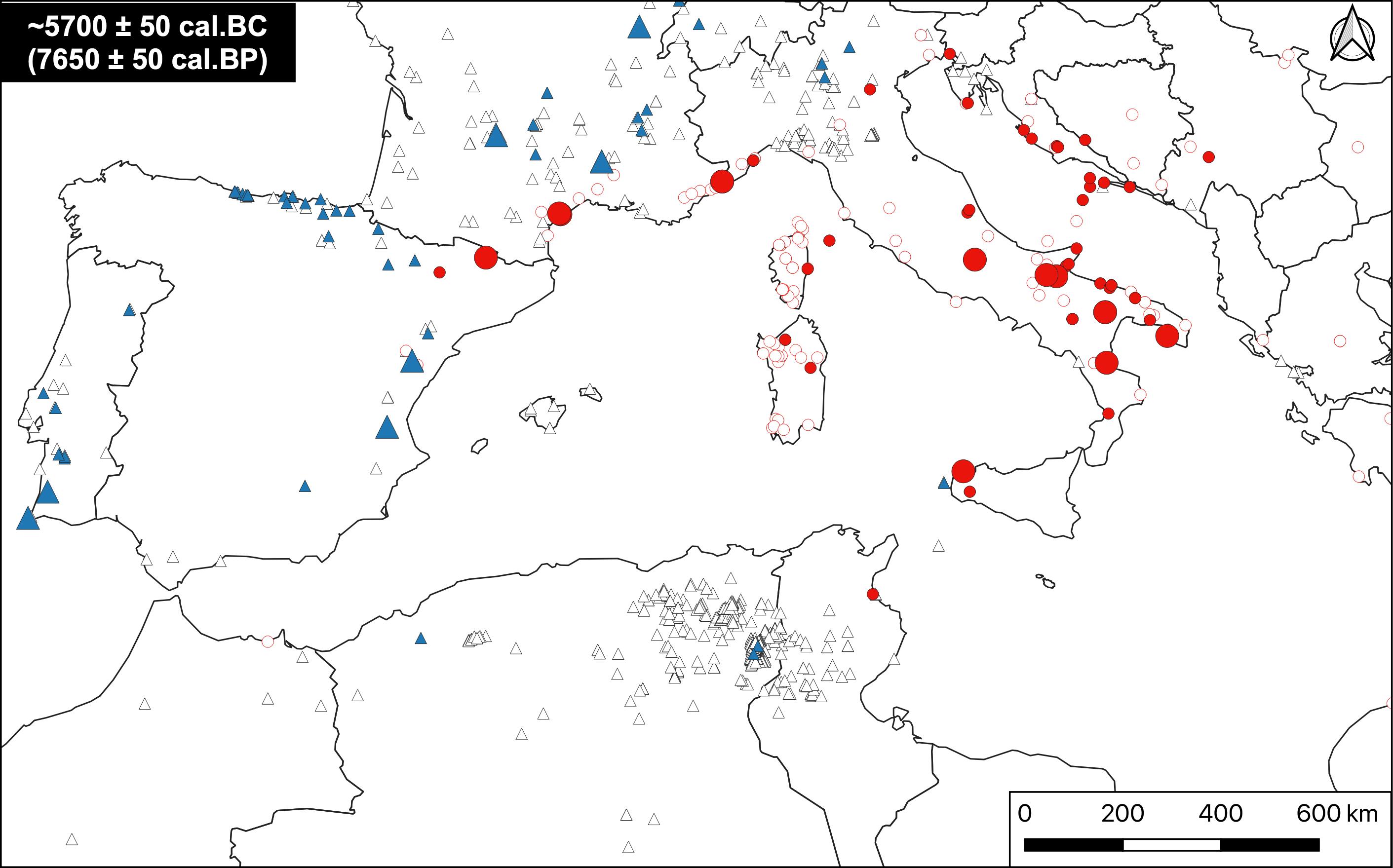

Supplement: S1 File — The white symbols relate to occupations with a reliability value of 3. The small full-colored symbols are reliability 2, and the large ones are reliability 1. Countries boundaries are from Natural Earth (free vector and raster map data @ naturalearthdata.com). (ZIP) [file pone.0246964.s003.zip › 5700-rel1_2_3.jpg]

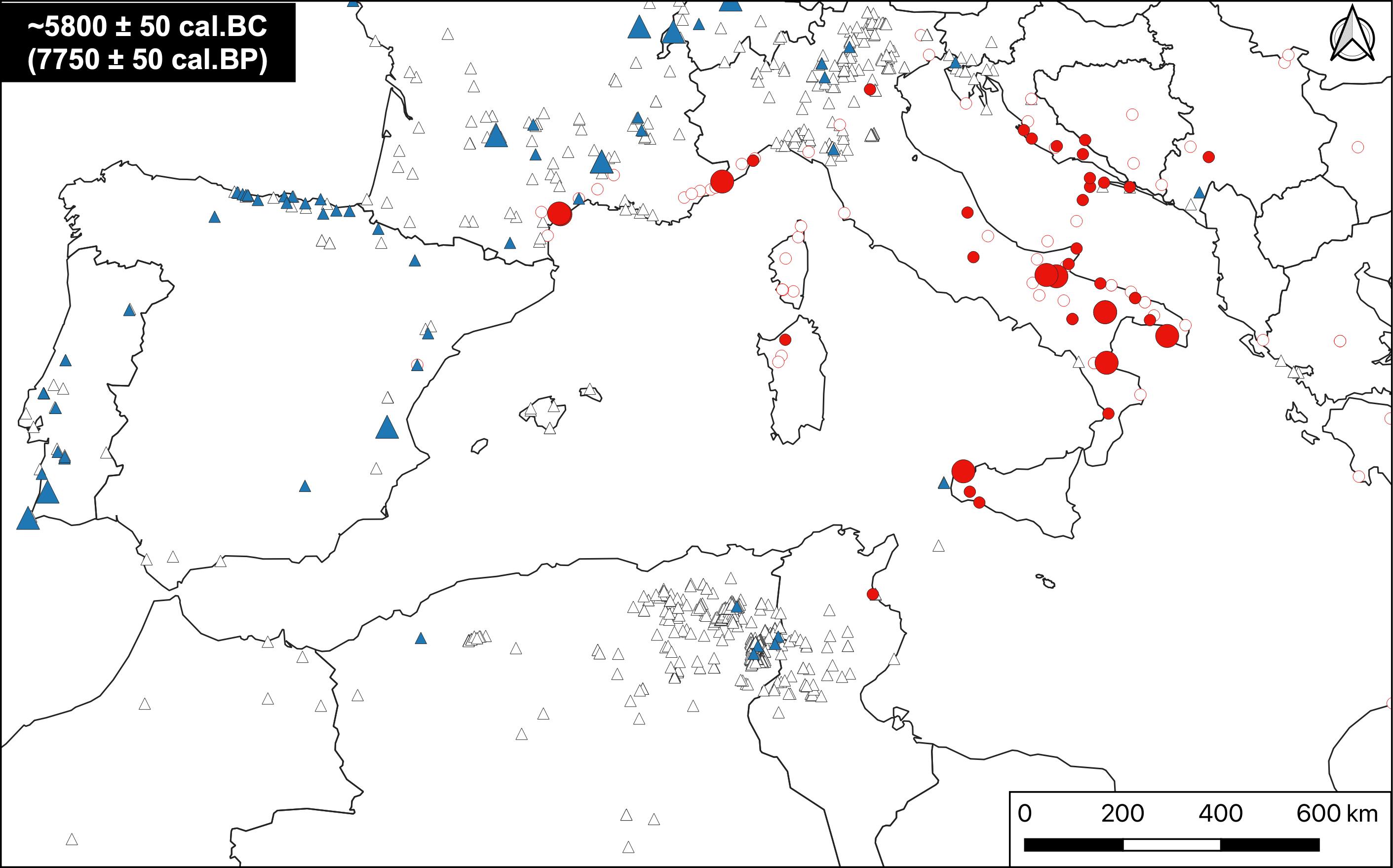

Supplement: S1 File — The white symbols relate to occupations with a reliability value of 3. The small full-colored symbols are reliability 2, and the large ones are reliability 1. Countries boundaries are from Natural Earth (free vector and raster map data @ naturalearthdata.com). (ZIP) [file pone.0246964.s003.zip › 5800-rel1_2_3.jpg]

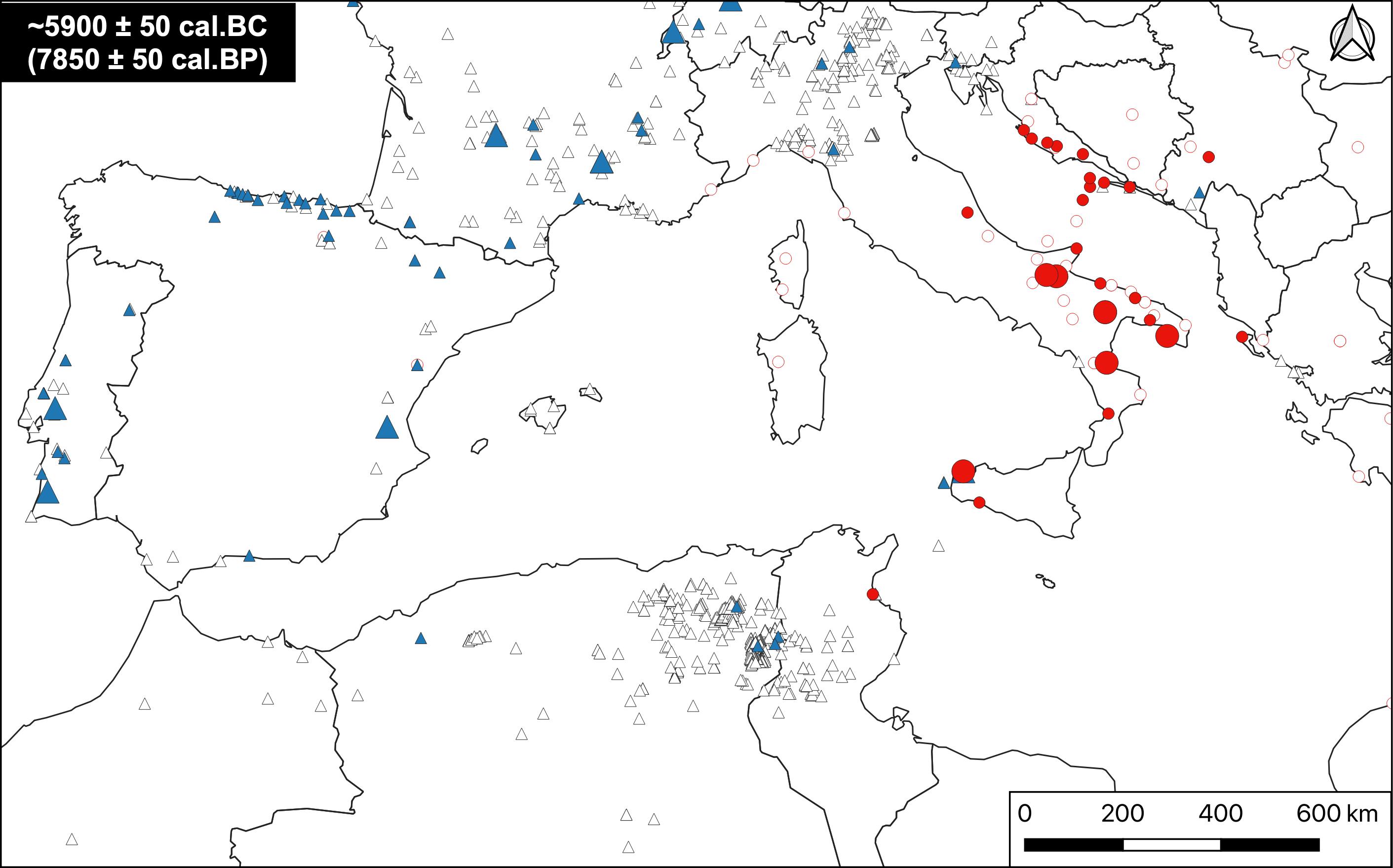

Supplement: S1 File — The white symbols relate to occupations with a reliability value of 3. The small full-colored symbols are reliability 2, and the large ones are reliability 1. Countries boundaries are from Natural Earth (free vector and raster map data @ naturalearthdata.com). (ZIP) [file pone.0246964.s003.zip › 5900-rel1_2_3.jpg]

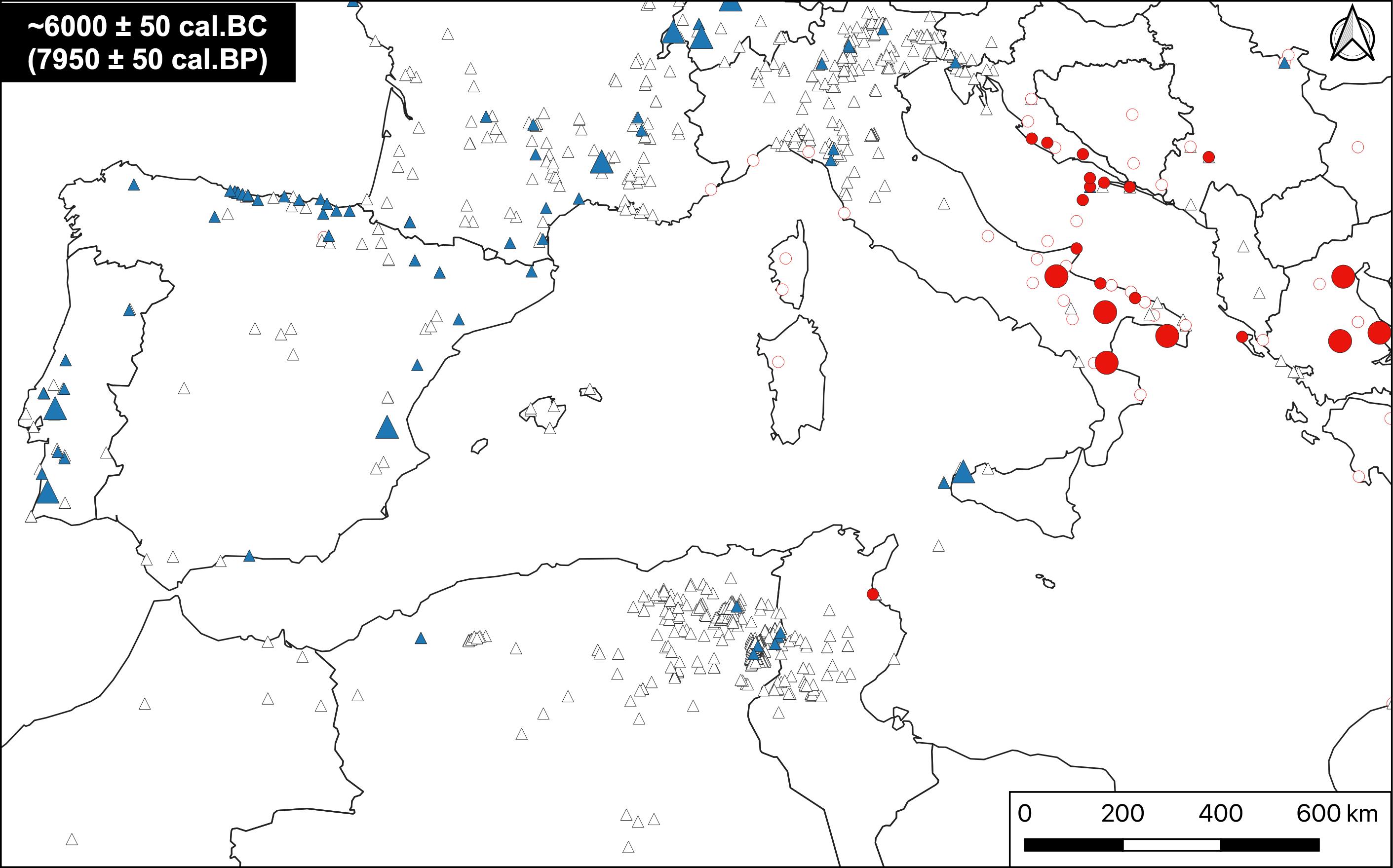

Supplement: S1 File — The white symbols relate to occupations with a reliability value of 3. The small full-colored symbols are reliability 2, and the large ones are reliability 1. Countries boundaries are from Natural Earth (free vector and raster map data @ naturalearthdata.com). (ZIP) [file pone.0246964.s003.zip › 6000-rel1_2_3.jpg]

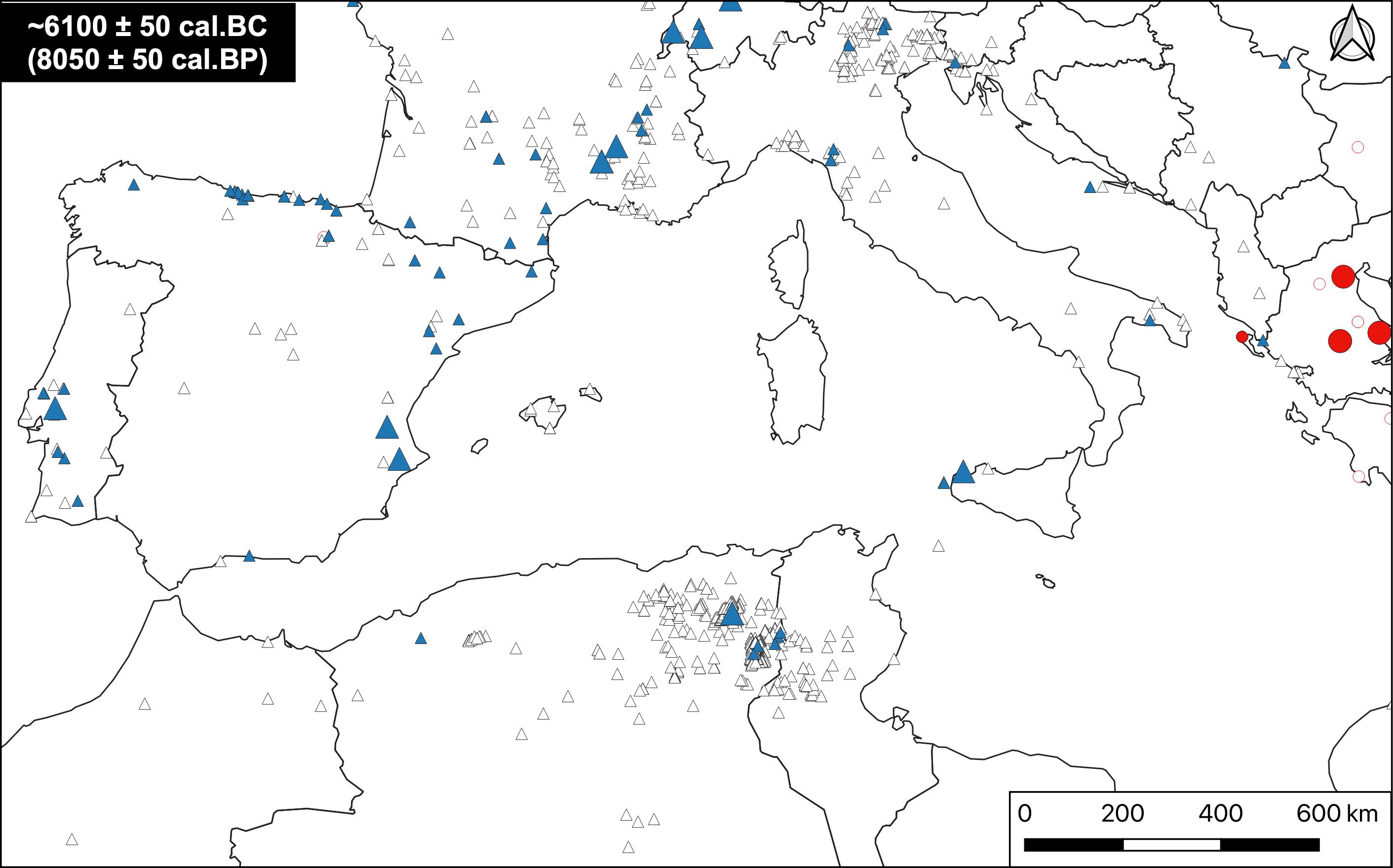

Supplement: S1 File — The white symbols relate to occupations with a reliability value of 3. The small full-colored symbols are reliability 2, and the large ones are reliability 1. Countries boundaries are from Natural Earth (free vector and raster map data @ naturalearthdata.com). (ZIP) [file pone.0246964.s003.zip › 6100-rel1_2_3.jpg]

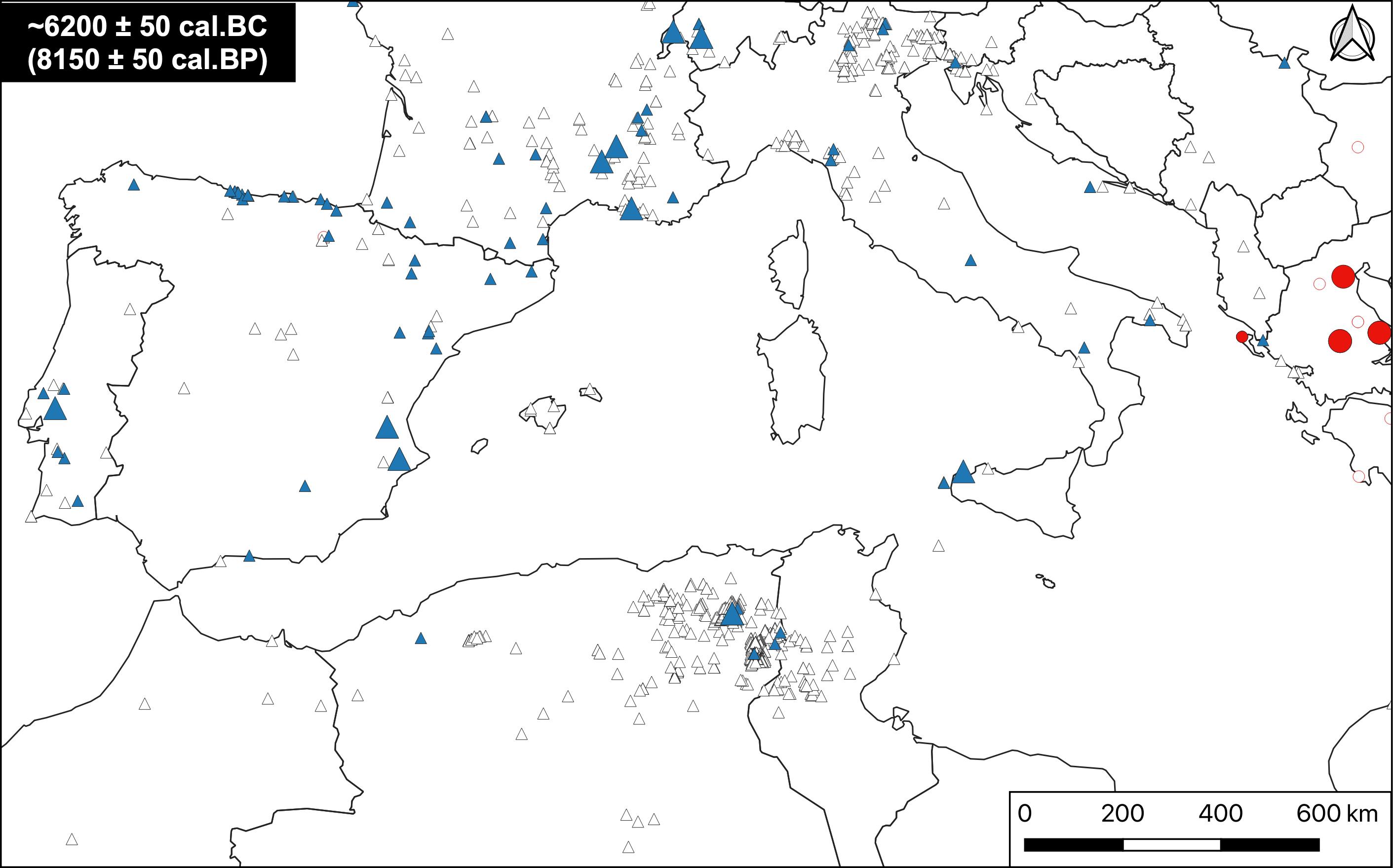

Supplement: S1 File — The white symbols relate to occupations with a reliability value of 3. The small full-colored symbols are reliability 2, and the large ones are reliability 1. Countries boundaries are from Natural Earth (free vector and raster map data @ naturalearthdata.com). (ZIP) [file pone.0246964.s003.zip › 6200-rel1_2_3.jpg]

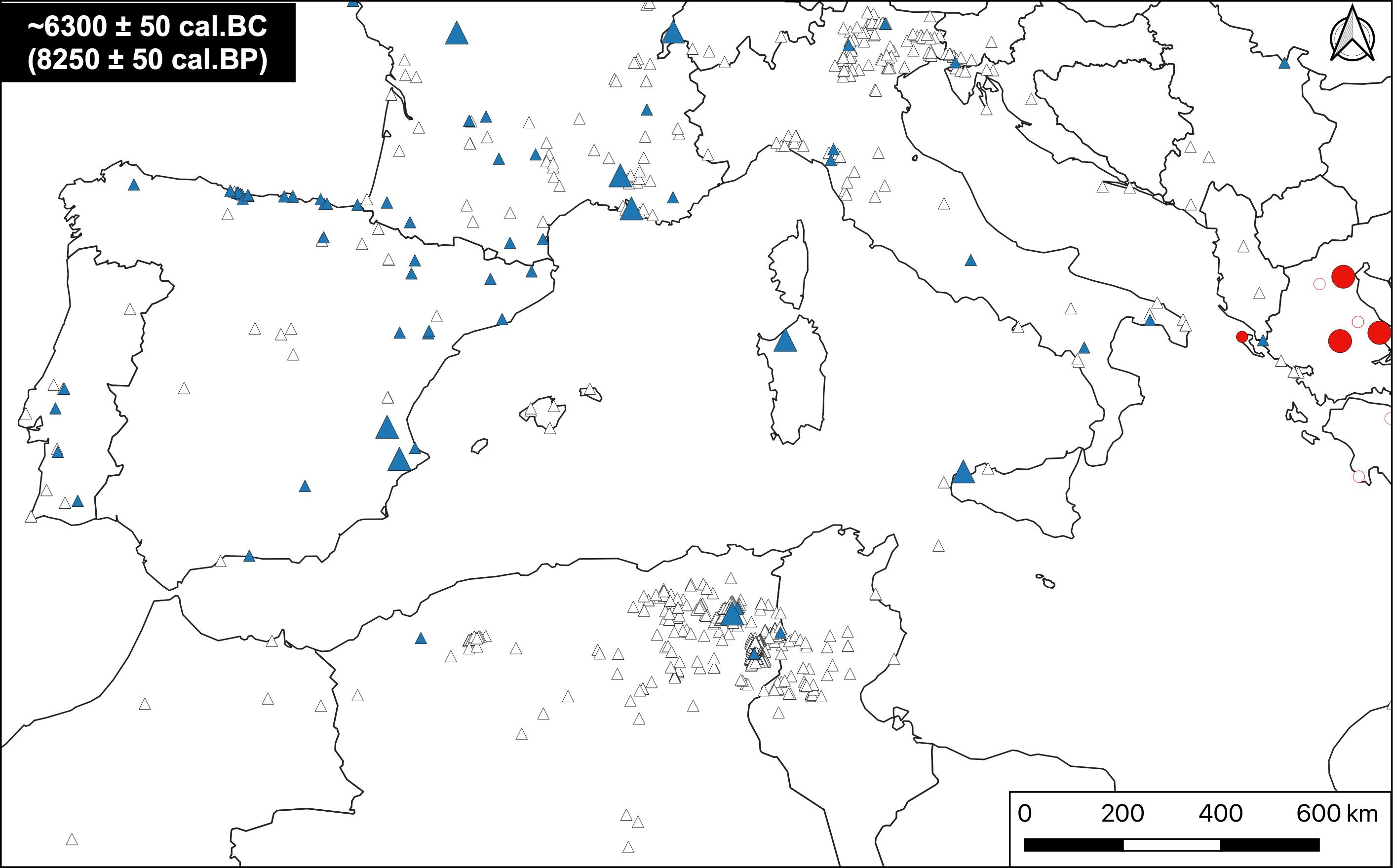

Supplement: S1 File — The white symbols relate to occupations with a reliability value of 3. The small full-colored symbols are reliability 2, and the large ones are reliability 1. Countries boundaries are from Natural Earth (free vector and raster map data @ naturalearthdata.com). (ZIP) [file pone.0246964.s003.zip › 6300-rel1_2_3.jpg]

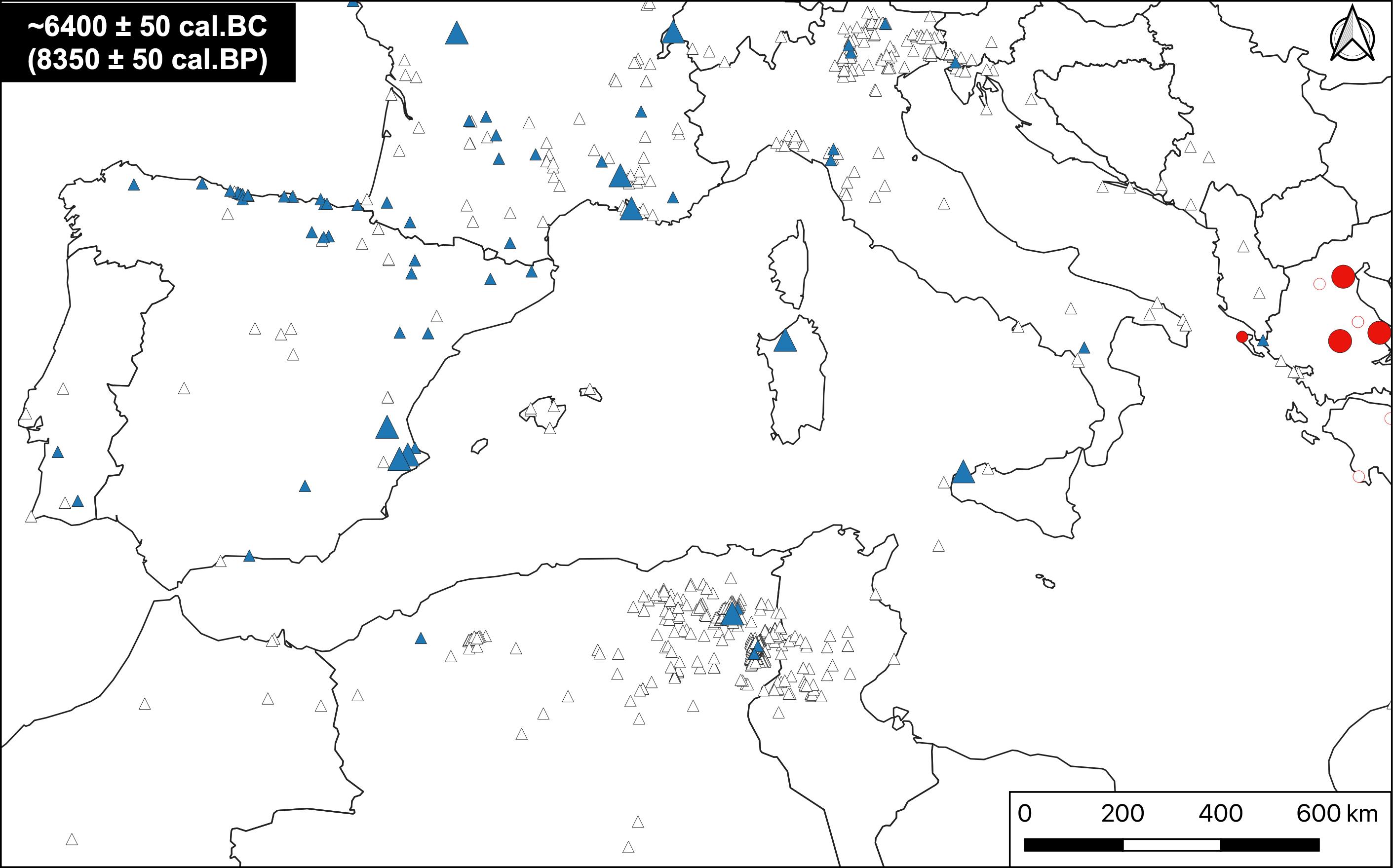

Supplement: S1 File — The white symbols relate to occupations with a reliability value of 3. The small full-colored symbols are reliability 2, and the large ones are reliability 1. Countries boundaries are from Natural Earth (free vector and raster map data @ naturalearthdata.com). (ZIP) [file pone.0246964.s003.zip › 6400-rel1_2_3.jpg]

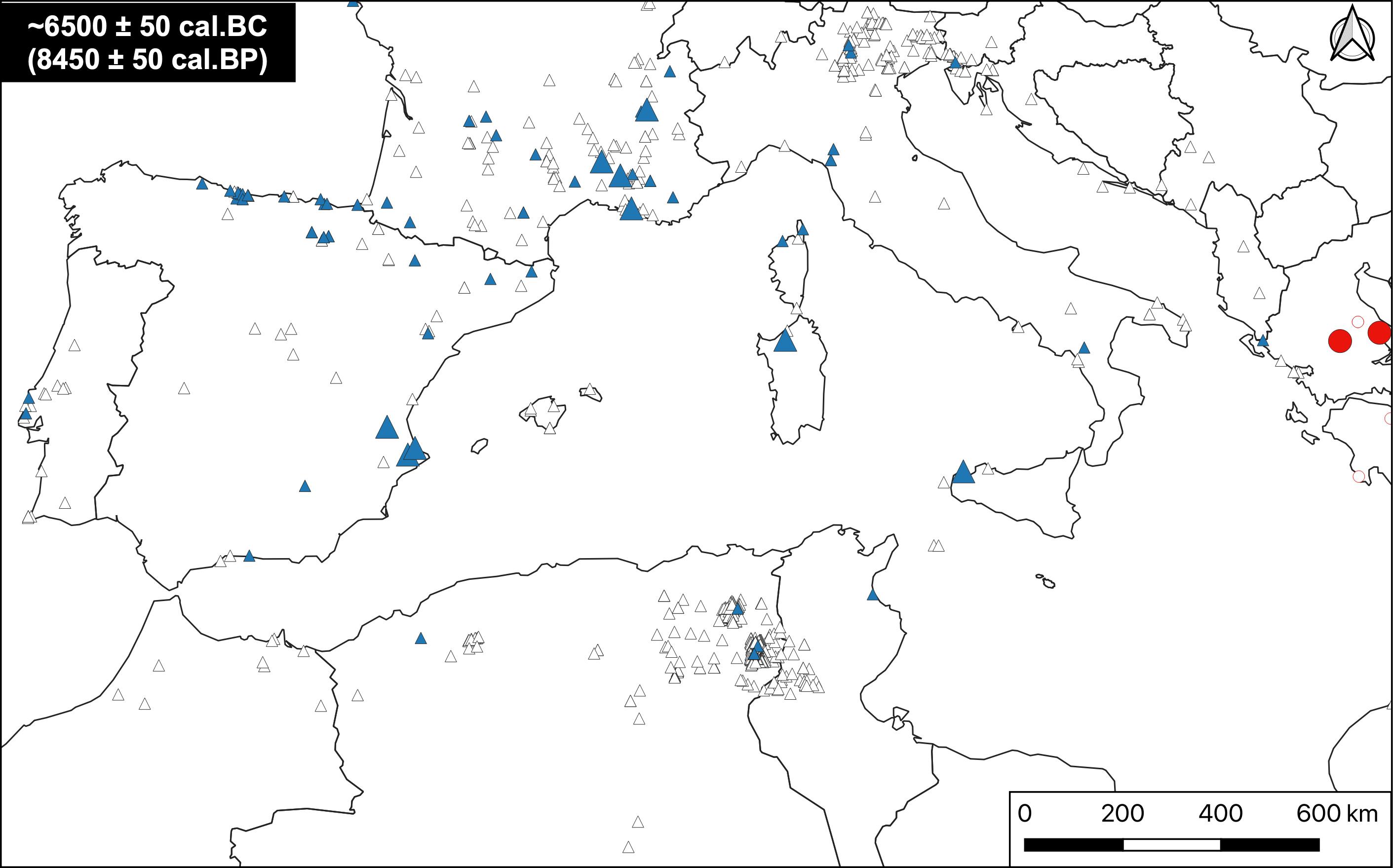

Supplement: S1 File — The white symbols relate to occupations with a reliability value of 3. The small full-colored symbols are reliability 2, and the large ones are reliability 1. Countries boundaries are from Natural Earth (free vector and raster map data @ naturalearthdata.com). (ZIP) [file pone.0246964.s003.zip › 6500-rel1_2_3.jpg]

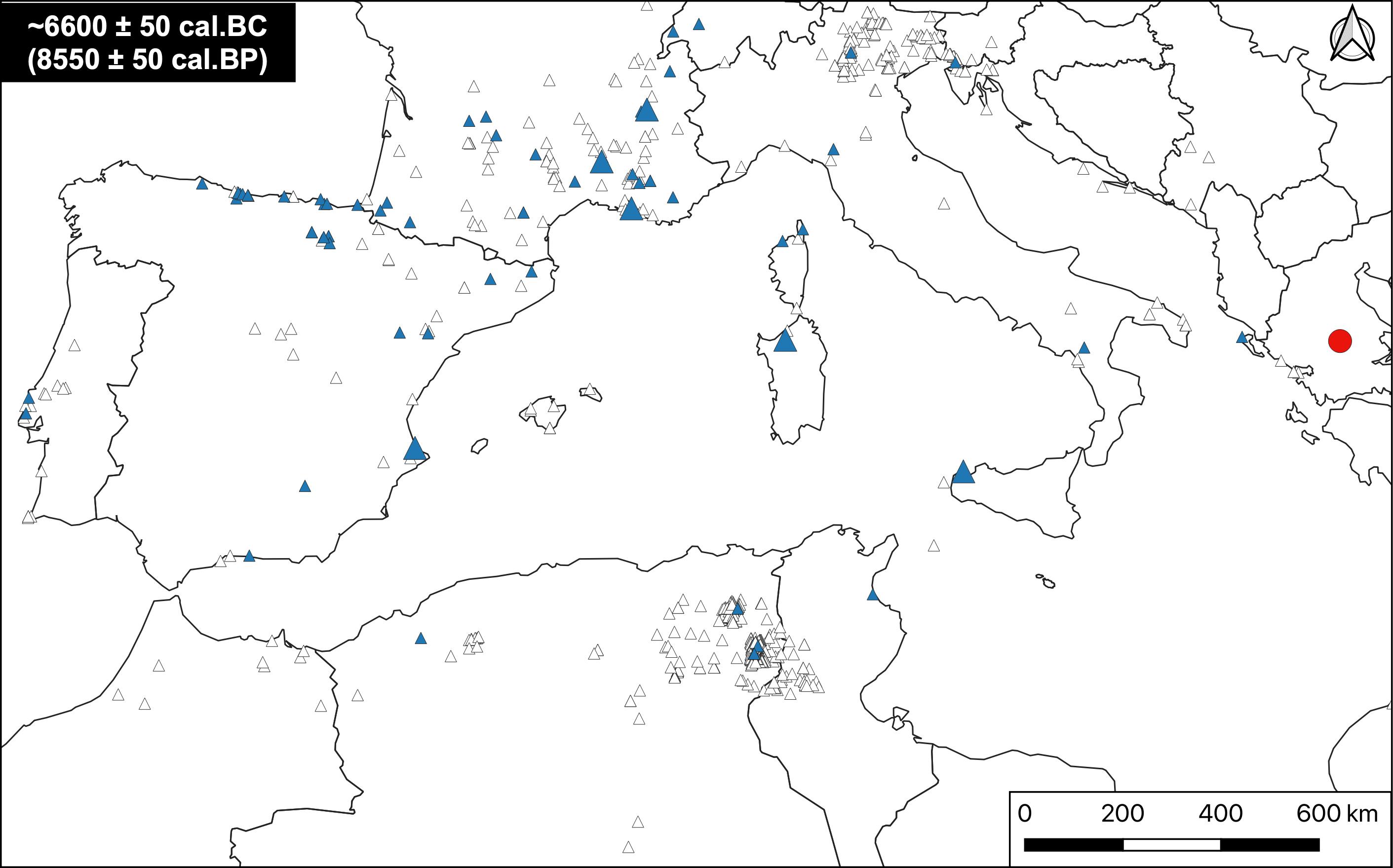

Supplement: S1 File — The white symbols relate to occupations with a reliability value of 3. The small full-colored symbols are reliability 2, and the large ones are reliability 1. Countries boundaries are from Natural Earth (free vector and raster map data @ naturalearthdata.com). (ZIP) [file pone.0246964.s003.zip › 6600-rel1_2_3.jpg]

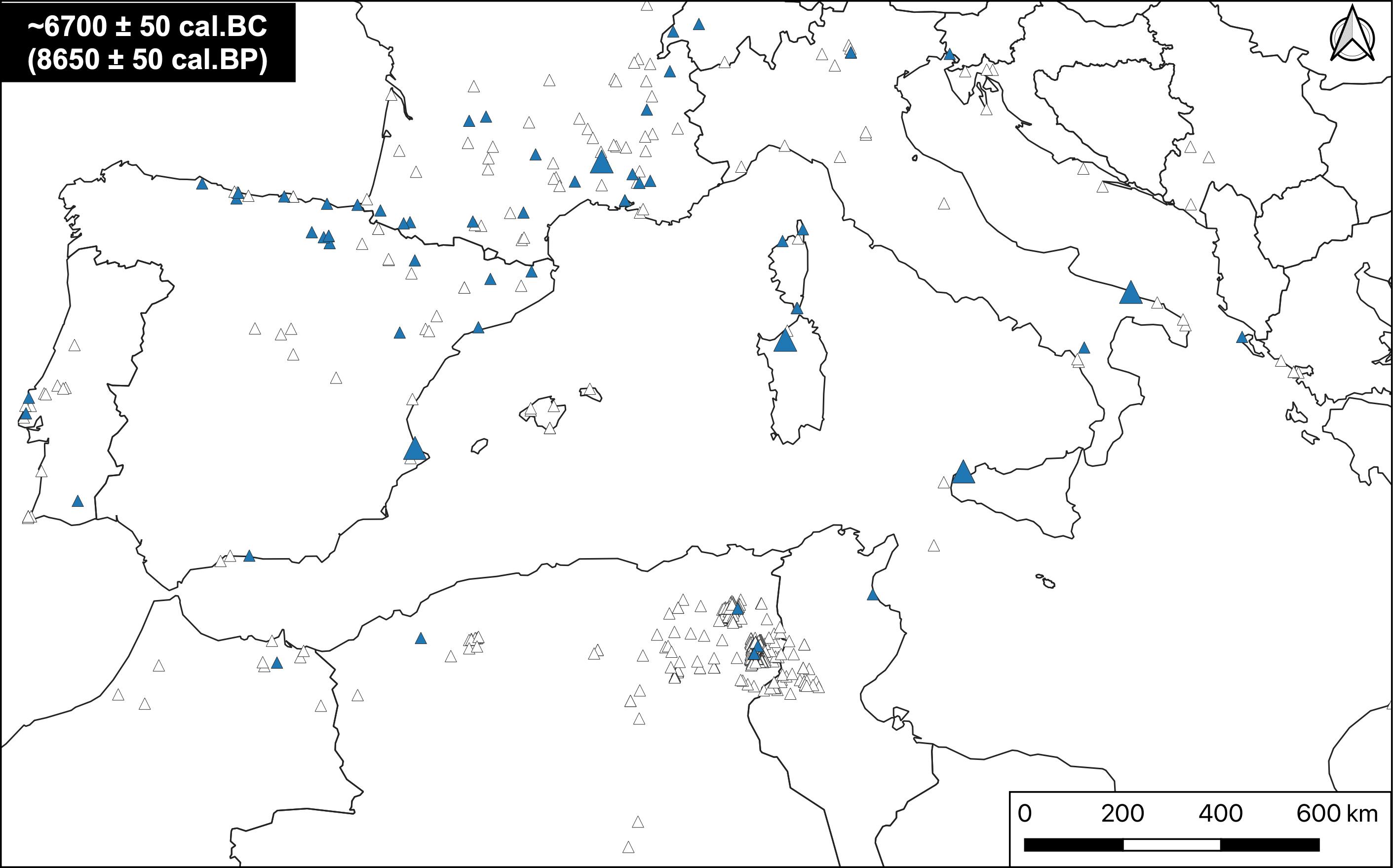

Supplement: S1 File — The white symbols relate to occupations with a reliability value of 3. The small full-colored symbols are reliability 2, and the large ones are reliability 1. Countries boundaries are from Natural Earth (free vector and raster map data @ naturalearthdata.com). (ZIP) [file pone.0246964.s003.zip › 6700-rel1_2_3.jpg]

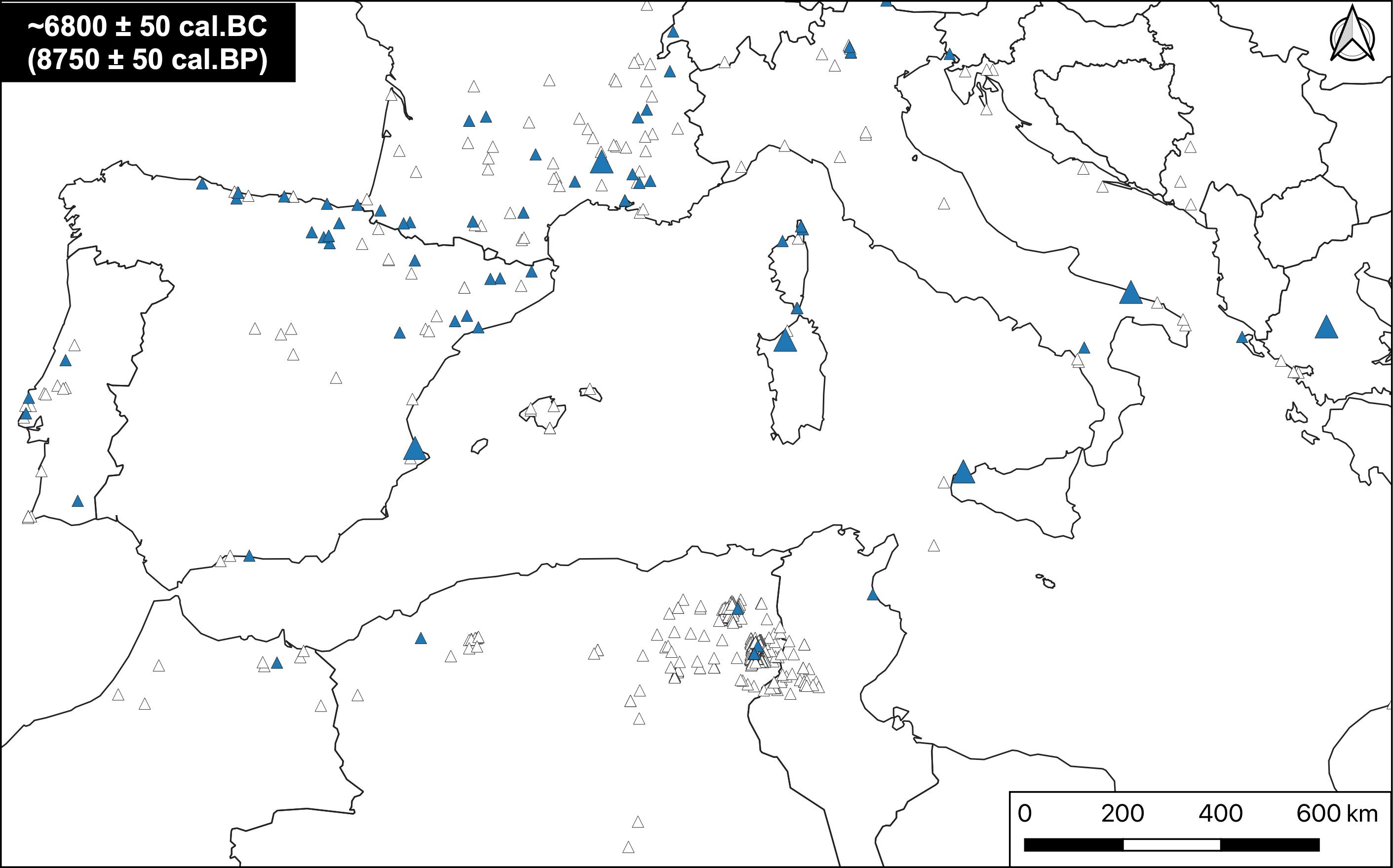

Supplement: S1 File — The white symbols relate to occupations with a reliability value of 3. The small full-colored symbols are reliability 2, and the large ones are reliability 1. Countries boundaries are from Natural Earth (free vector and raster map data @ naturalearthdata.com). (ZIP) [file pone.0246964.s003.zip › 6800-rel1_2_3.jpg]

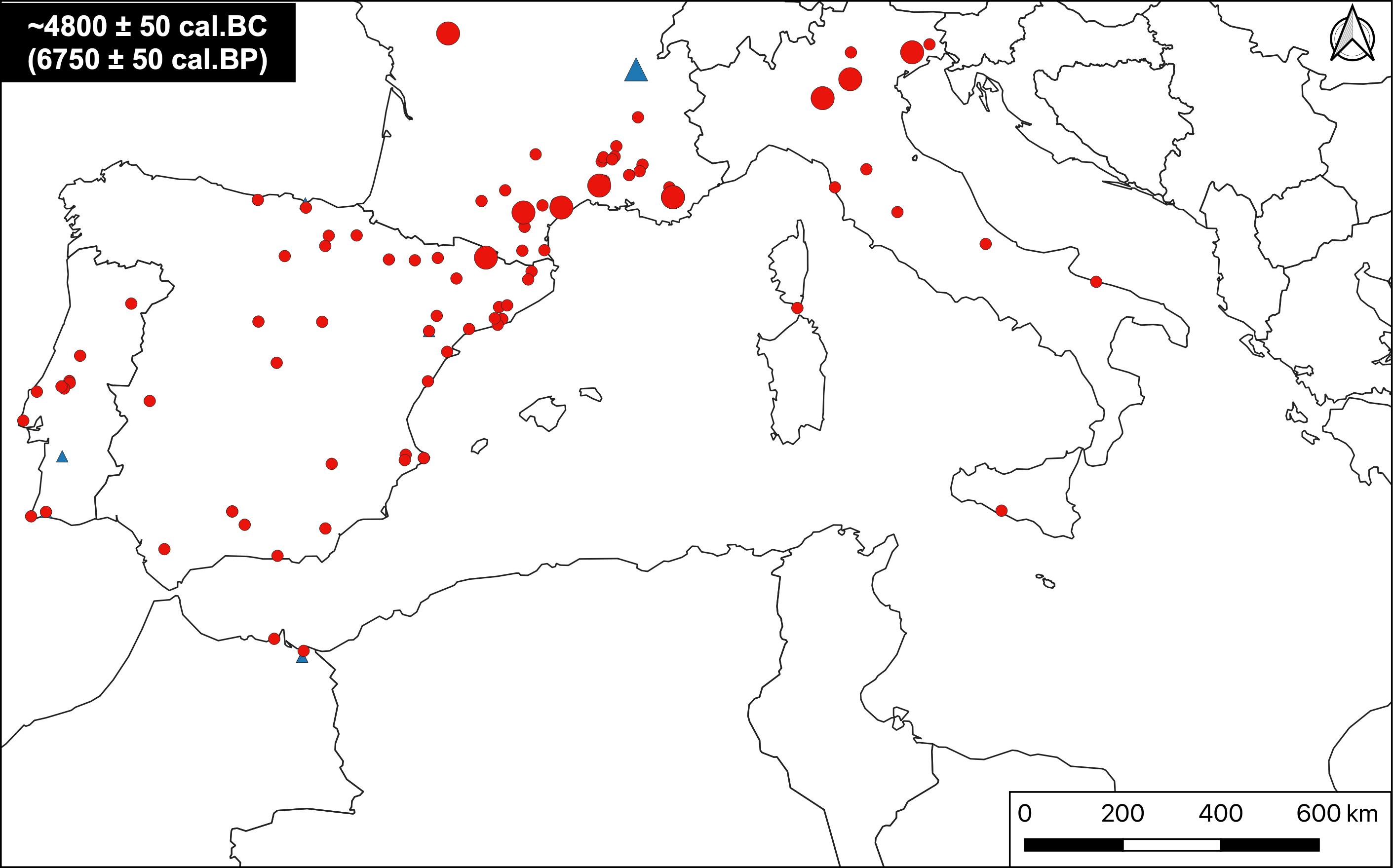

Supplement: S2 File — The small full colored symbols relate to occupations with a reliability value of 2, the large ones are reliability 1. Countries boundaries are from Natural Earth (free vector and raster map data @ naturalearthdata.com). (ZIP) [file pone.0246964.s004.zip › 4800-rel1_2.jpg]

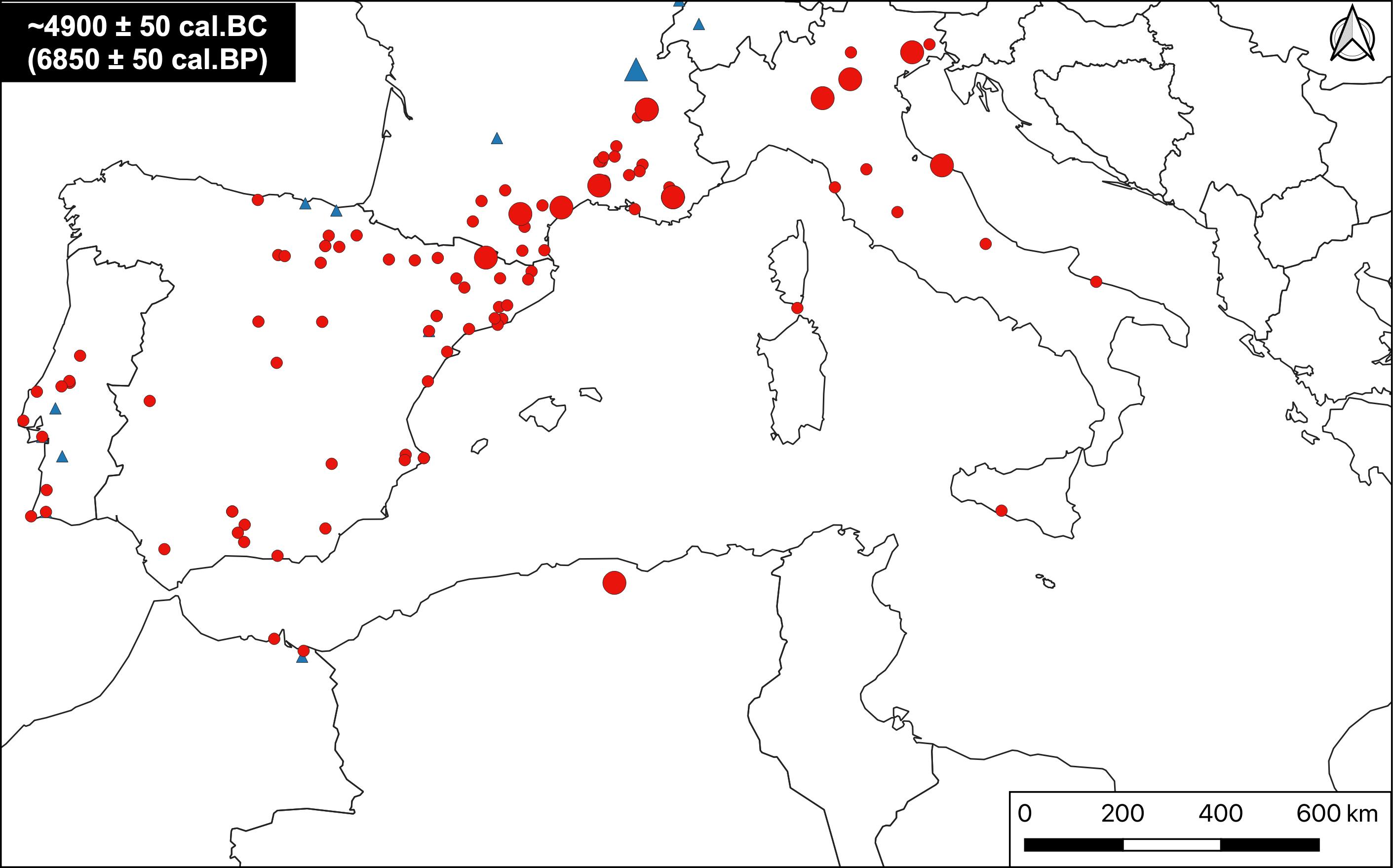

Supplement: S2 File — The small full colored symbols relate to occupations with a reliability value of 2, the large ones are reliability 1. Countries boundaries are from Natural Earth (free vector and raster map data @ naturalearthdata.com). (ZIP) [file pone.0246964.s004.zip › 4900-rel1_2.jpg]

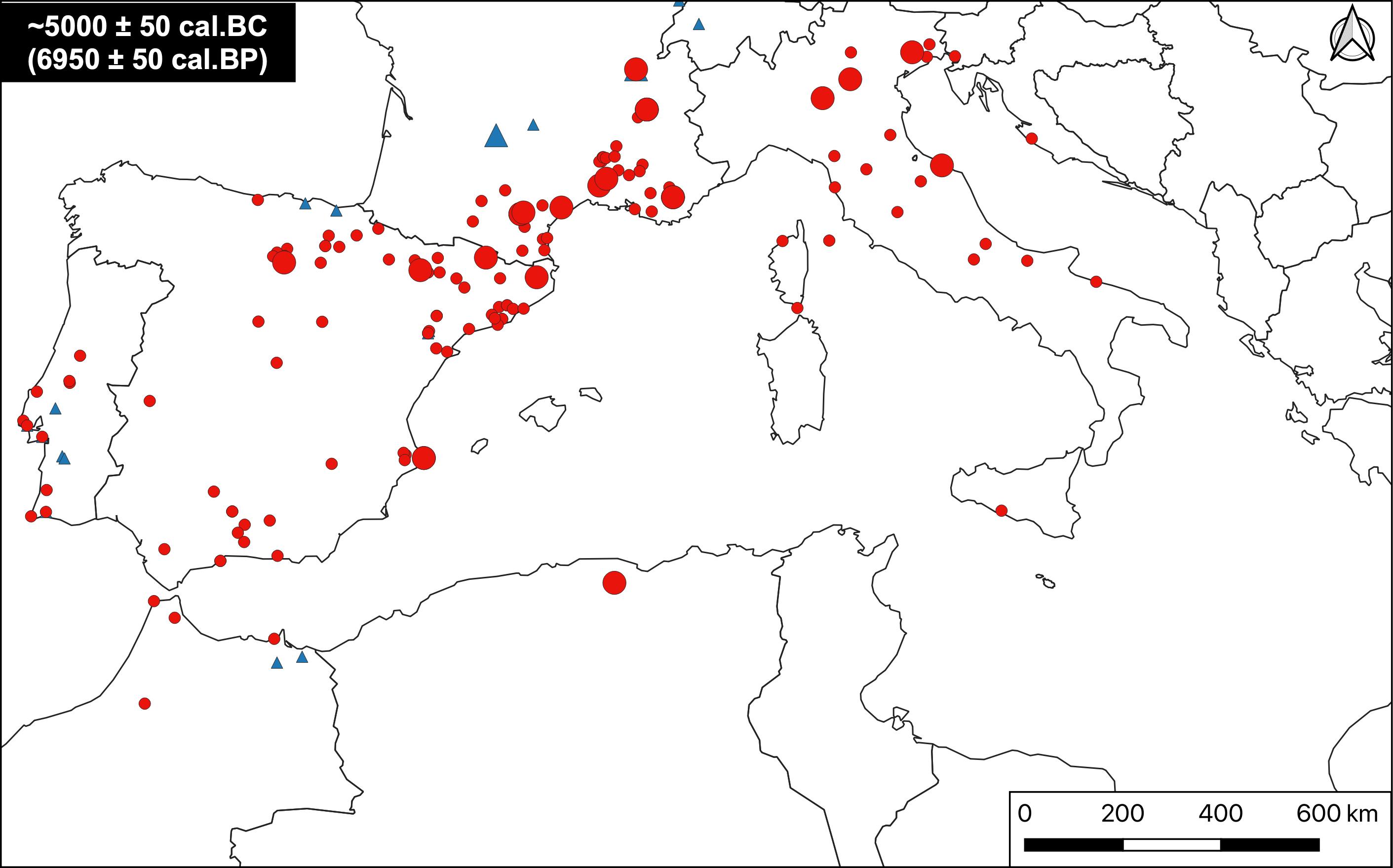

Supplement: S2 File — The small full colored symbols relate to occupations with a reliability value of 2, the large ones are reliability 1. Countries boundaries are from Natural Earth (free vector and raster map data @ naturalearthdata.com). (ZIP) [file pone.0246964.s004.zip › 5000-rel1_2.jpg]

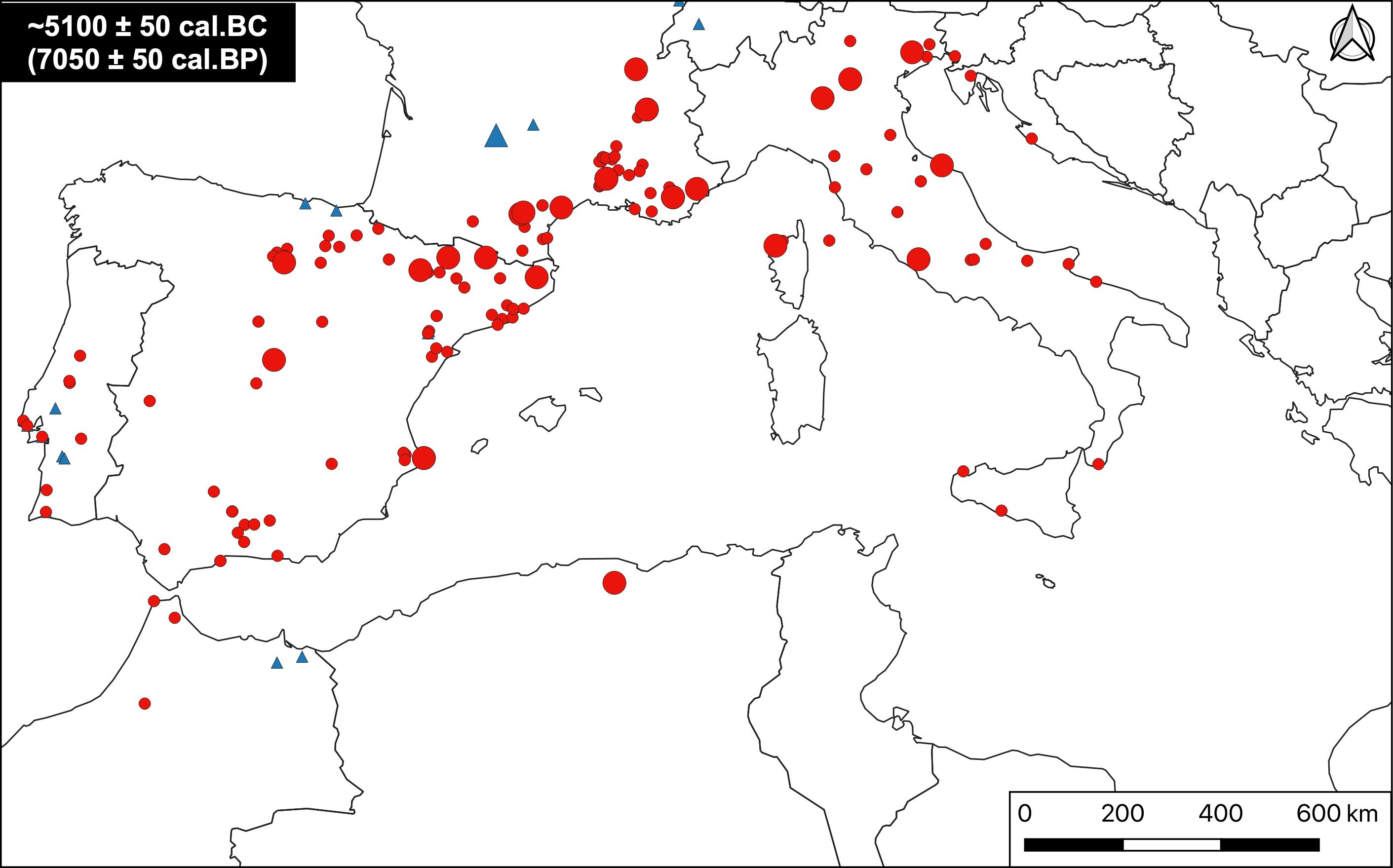

Supplement: S2 File — The small full colored symbols relate to occupations with a reliability value of 2, the large ones are reliability 1. Countries boundaries are from Natural Earth (free vector and raster map data @ naturalearthdata.com). (ZIP) [file pone.0246964.s004.zip › 5100-rel1_2.jpg]

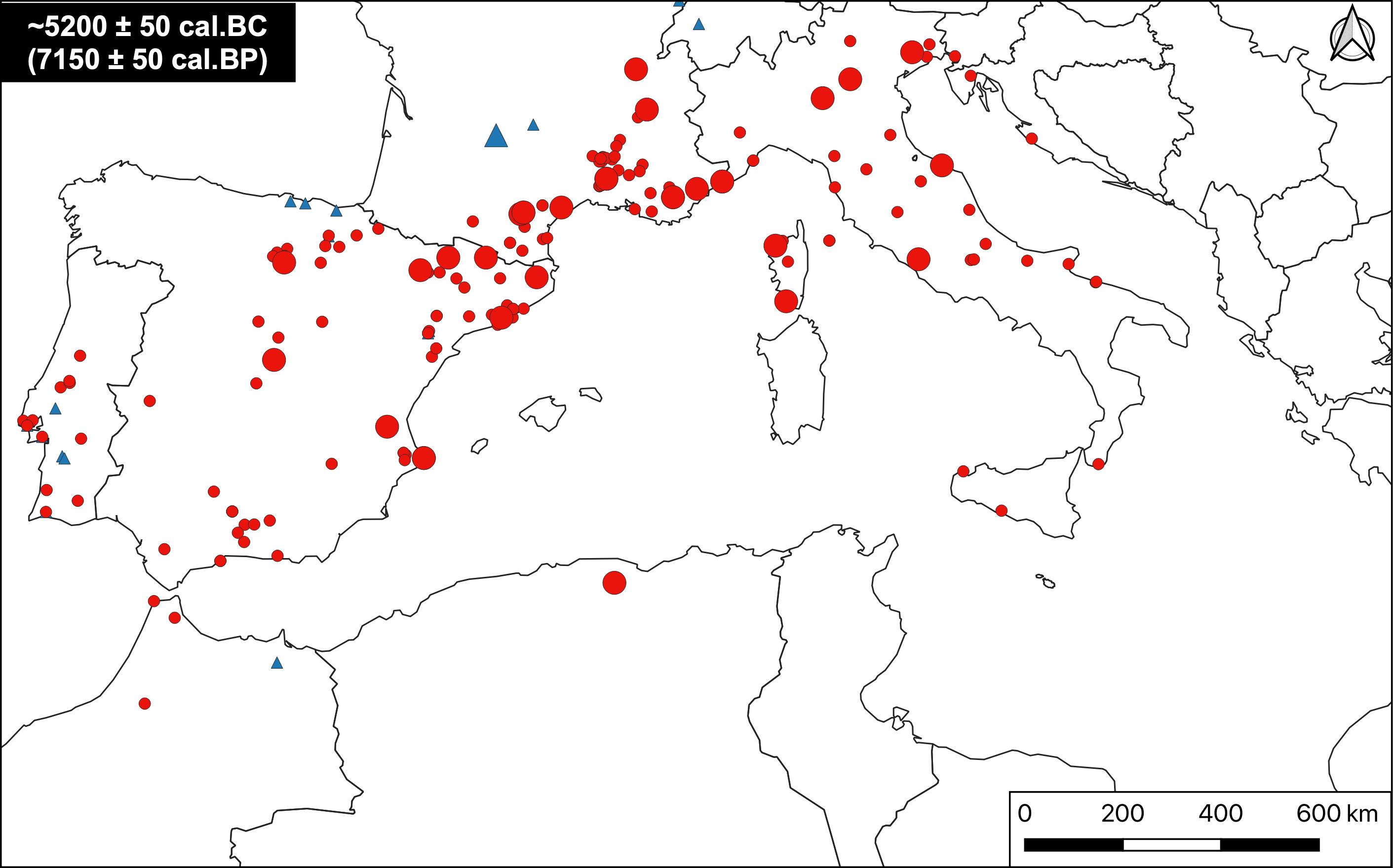

Supplement: S2 File — The small full colored symbols relate to occupations with a reliability value of 2, the large ones are reliability 1. Countries boundaries are from Natural Earth (free vector and raster map data @ naturalearthdata.com). (ZIP) [file pone.0246964.s004.zip › 5200-rel1_2.jpg]

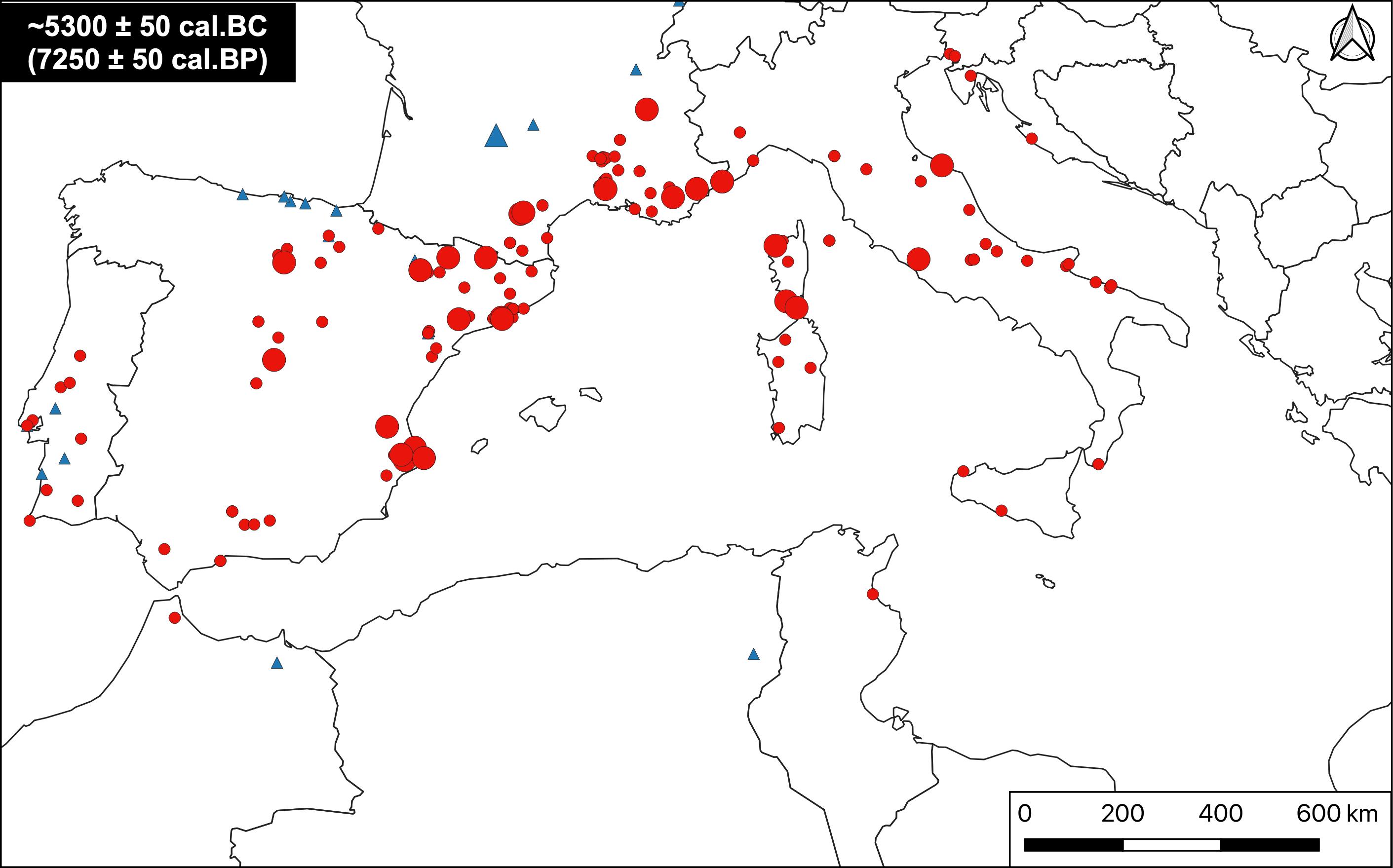

Supplement: S2 File — The small full colored symbols relate to occupations with a reliability value of 2, the large ones are reliability 1. Countries boundaries are from Natural Earth (free vector and raster map data @ naturalearthdata.com). (ZIP) [file pone.0246964.s004.zip › 5300-rel1_2.jpg]

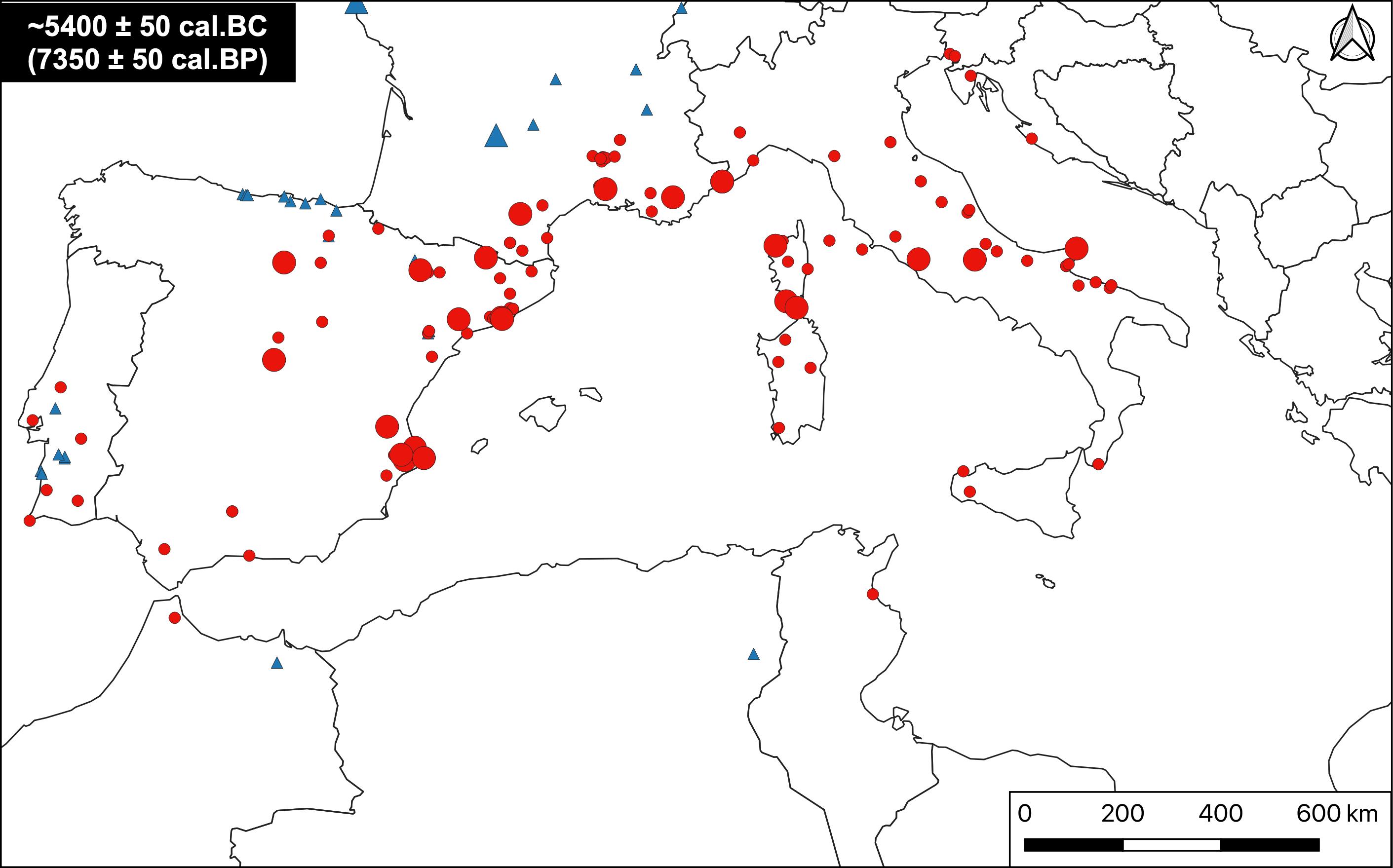

Supplement: S2 File — The small full colored symbols relate to occupations with a reliability value of 2, the large ones are reliability 1. Countries boundaries are from Natural Earth (free vector and raster map data @ naturalearthdata.com). (ZIP) [file pone.0246964.s004.zip › 5400-rel1_2.jpg]

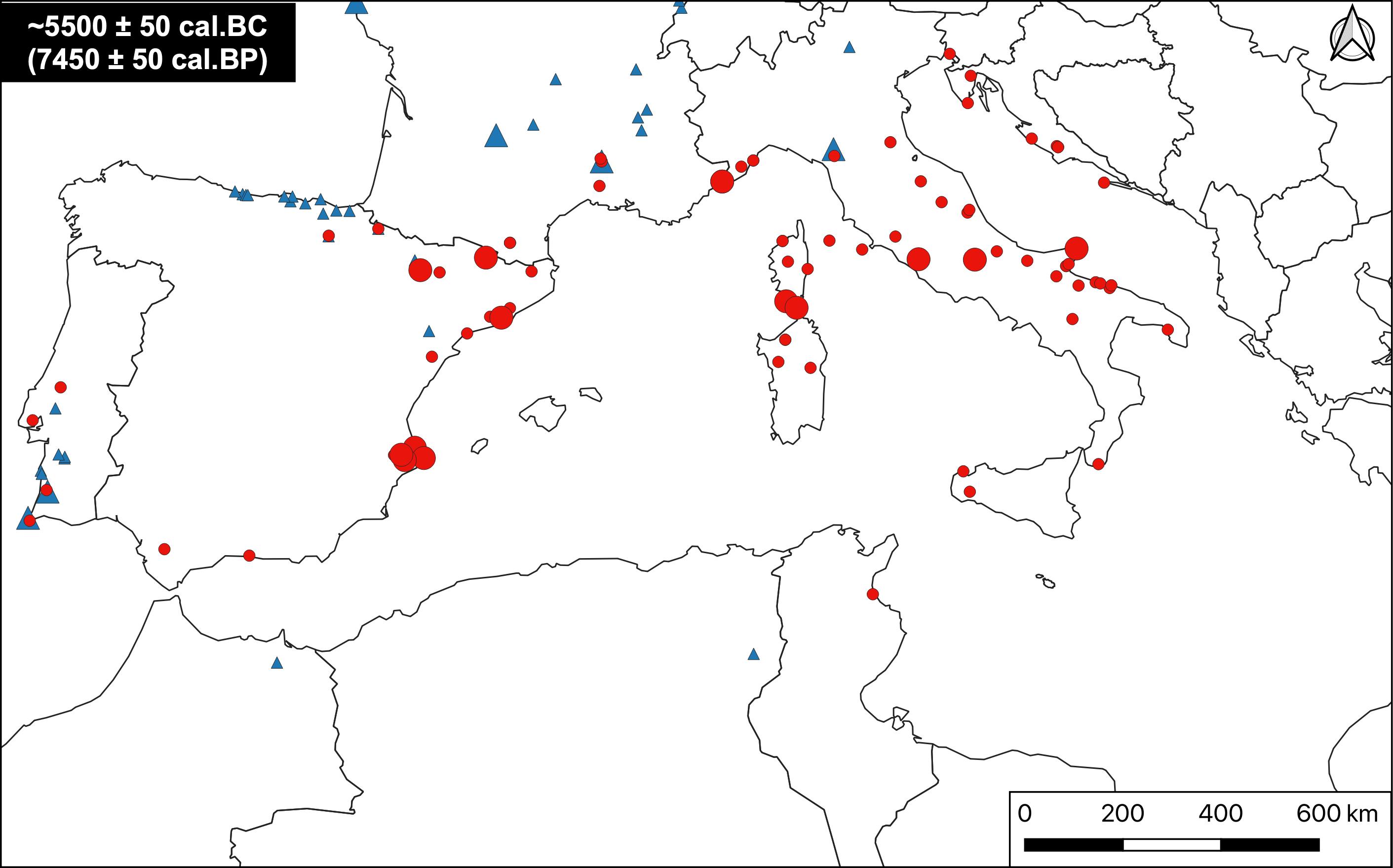

Supplement: S2 File — The small full colored symbols relate to occupations with a reliability value of 2, the large ones are reliability 1. Countries boundaries are from Natural Earth (free vector and raster map data @ naturalearthdata.com). (ZIP) [file pone.0246964.s004.zip › 5500-rel1_2.jpg]

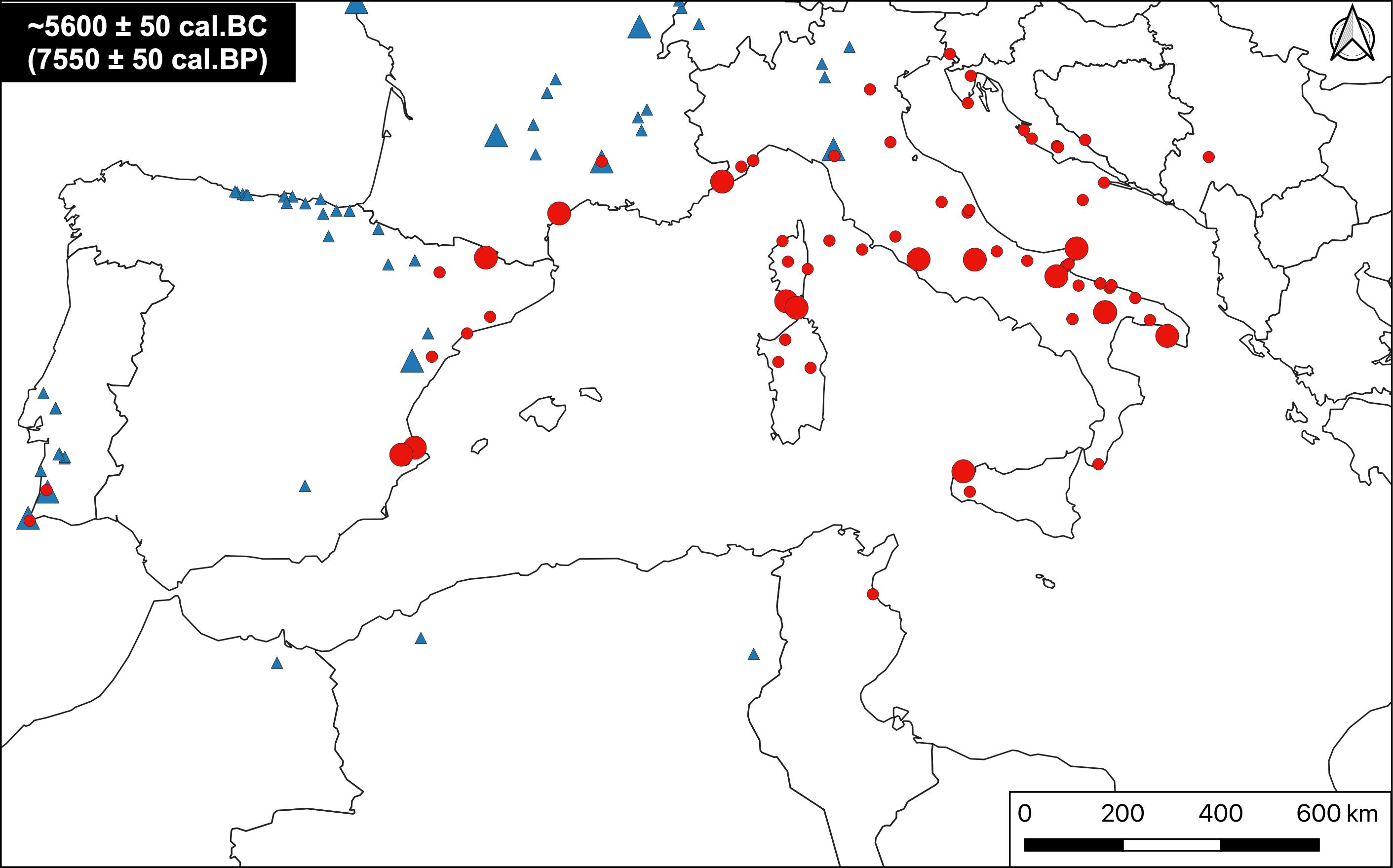

Supplement: S2 File — The small full colored symbols relate to occupations with a reliability value of 2, the large ones are reliability 1. Countries boundaries are from Natural Earth (free vector and raster map data @ naturalearthdata.com). (ZIP) [file pone.0246964.s004.zip › 5600-rel1_2.jpg]

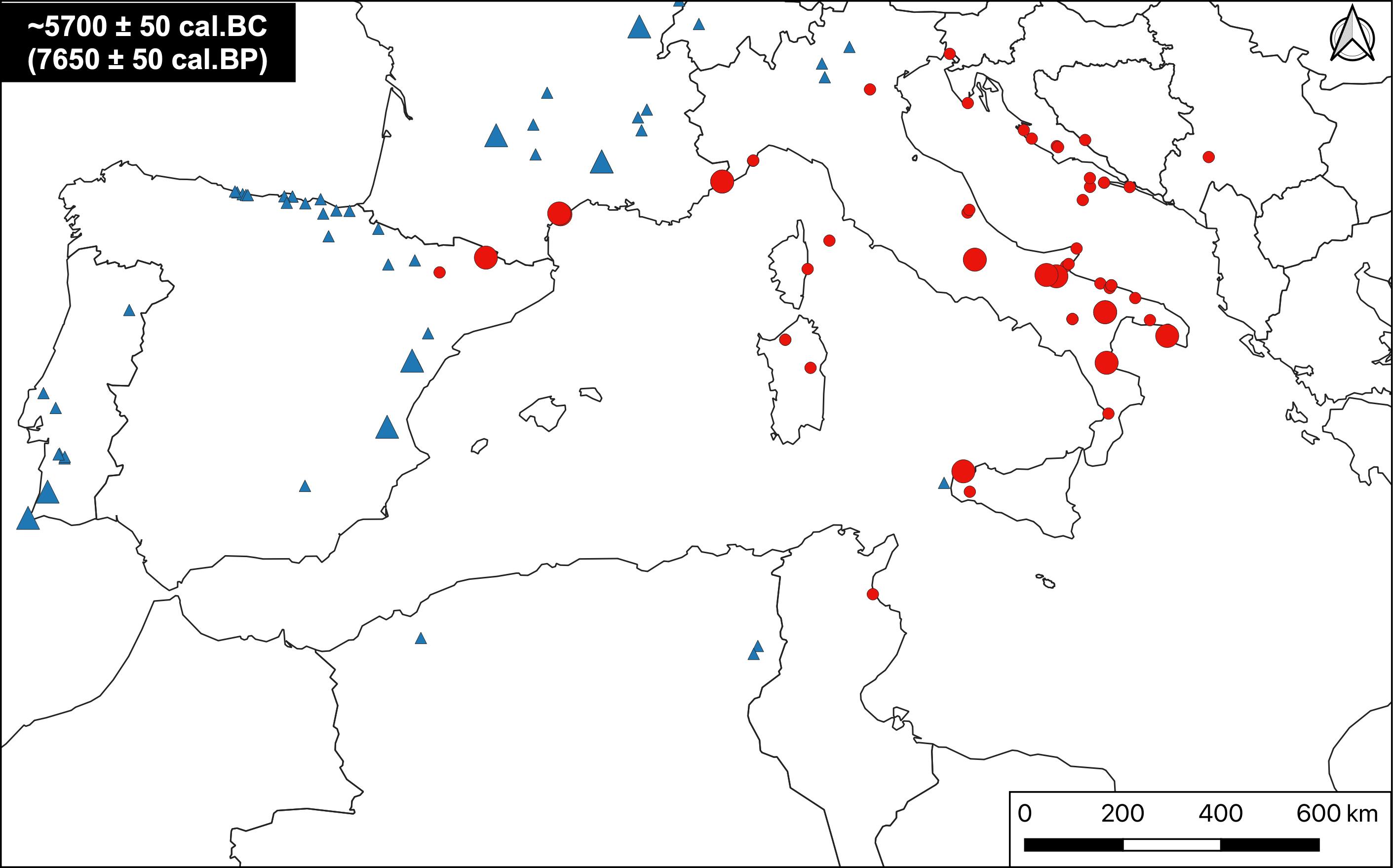

Supplement: S2 File — The small full colored symbols relate to occupations with a reliability value of 2, the large ones are reliability 1. Countries boundaries are from Natural Earth (free vector and raster map data @ naturalearthdata.com). (ZIP) [file pone.0246964.s004.zip › 5700-rel1_2.jpg]

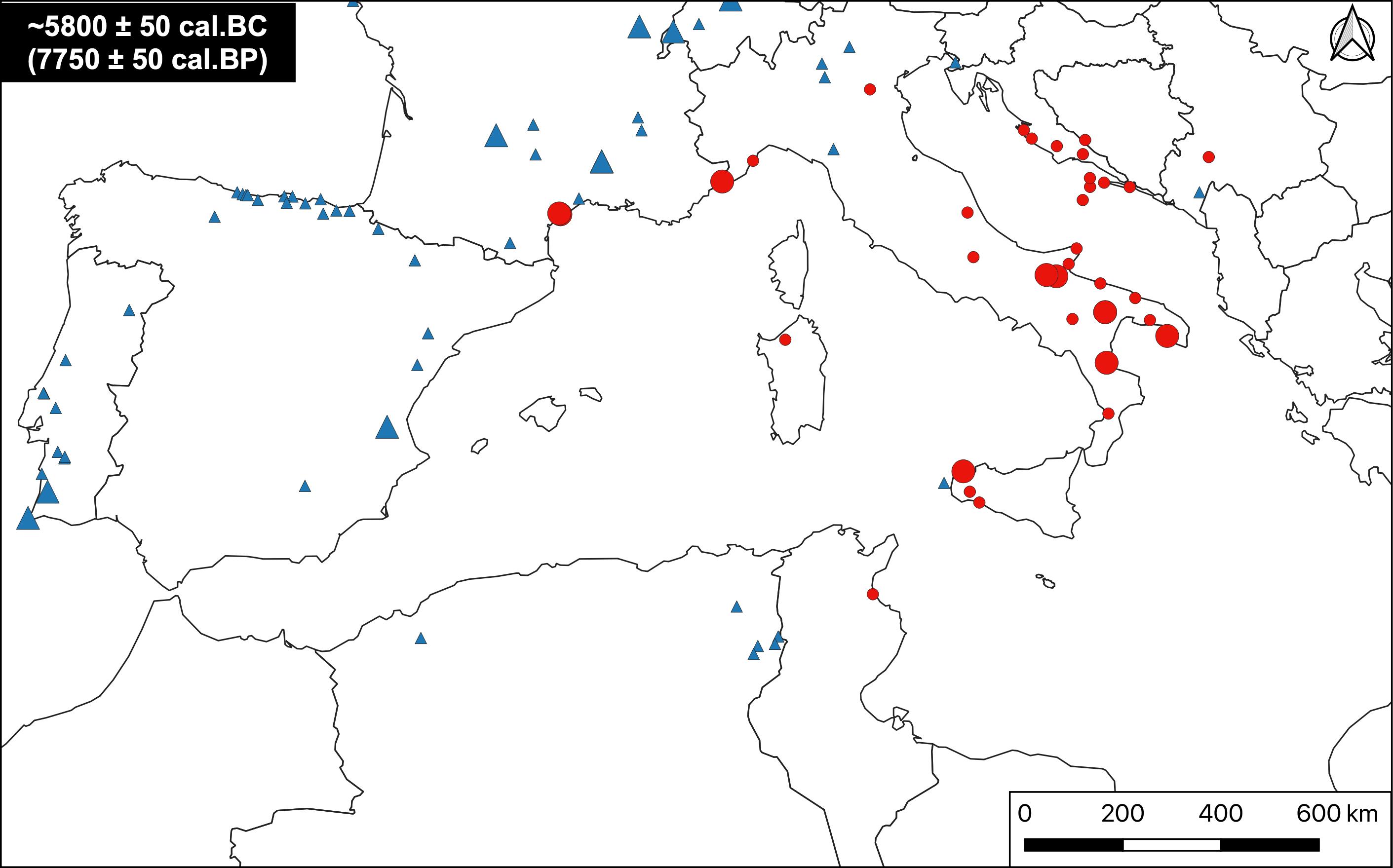

Supplement: S2 File — The small full colored symbols relate to occupations with a reliability value of 2, the large ones are reliability 1. Countries boundaries are from Natural Earth (free vector and raster map data @ naturalearthdata.com). (ZIP) [file pone.0246964.s004.zip › 5800-rel1_2.jpg]

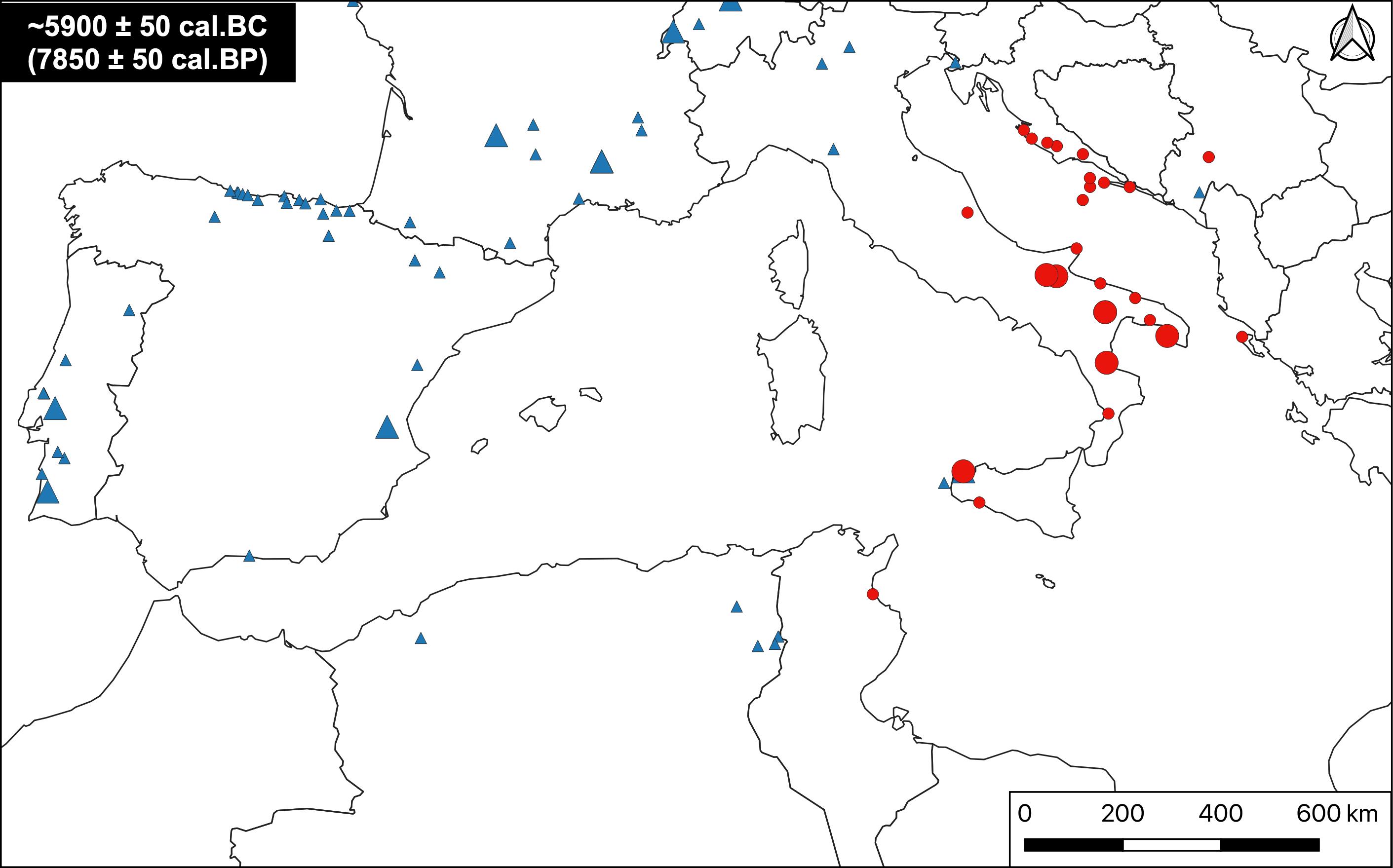

Supplement: S2 File — The small full colored symbols relate to occupations with a reliability value of 2, the large ones are reliability 1. Countries boundaries are from Natural Earth (free vector and raster map data @ naturalearthdata.com). (ZIP) [file pone.0246964.s004.zip › 5900-rel1_2.jpg]

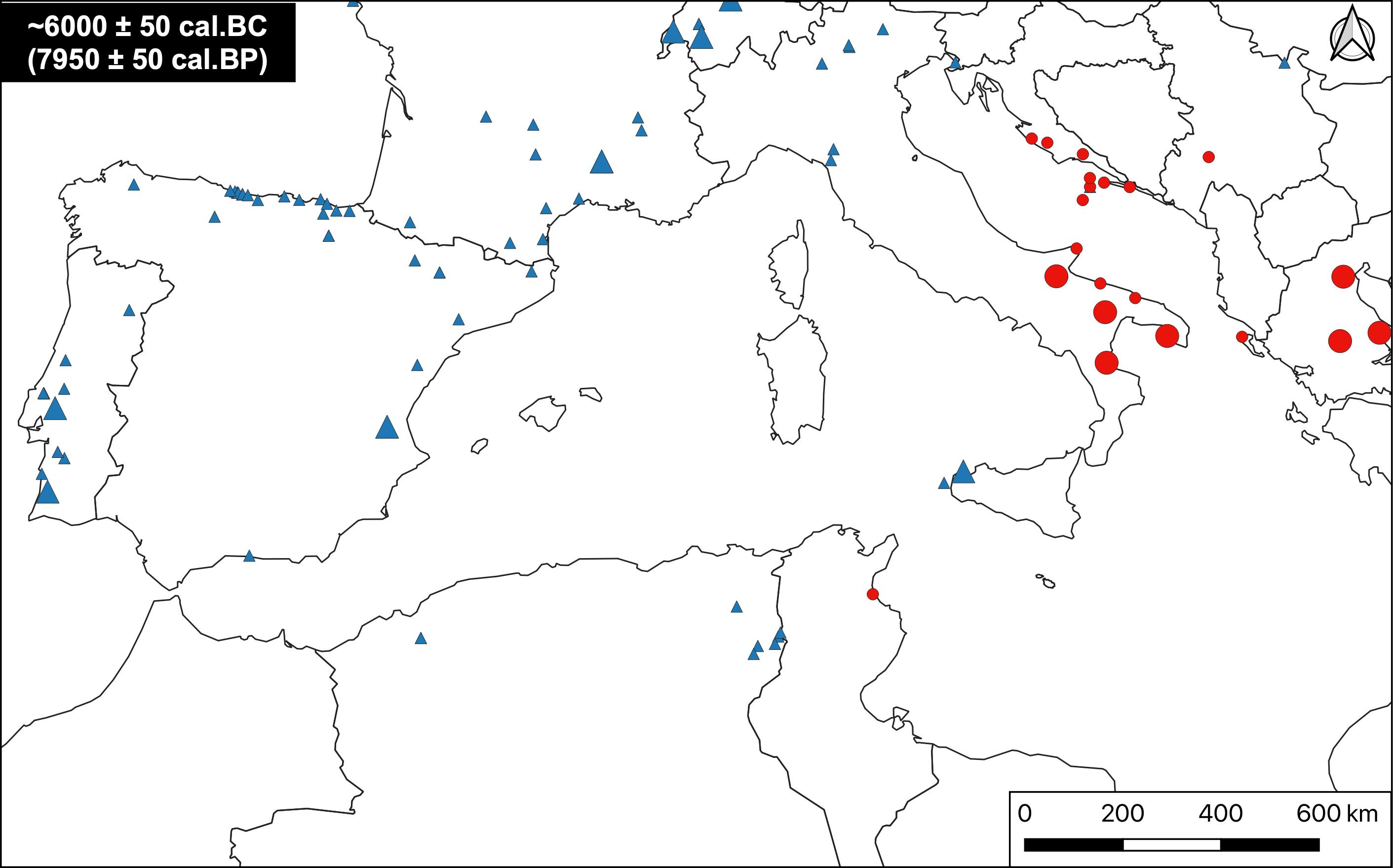

Supplement: S2 File — The small full colored symbols relate to occupations with a reliability value of 2, the large ones are reliability 1. Countries boundaries are from Natural Earth (free vector and raster map data @ naturalearthdata.com). (ZIP) [file pone.0246964.s004.zip › 6000-rel1_2.jpg]

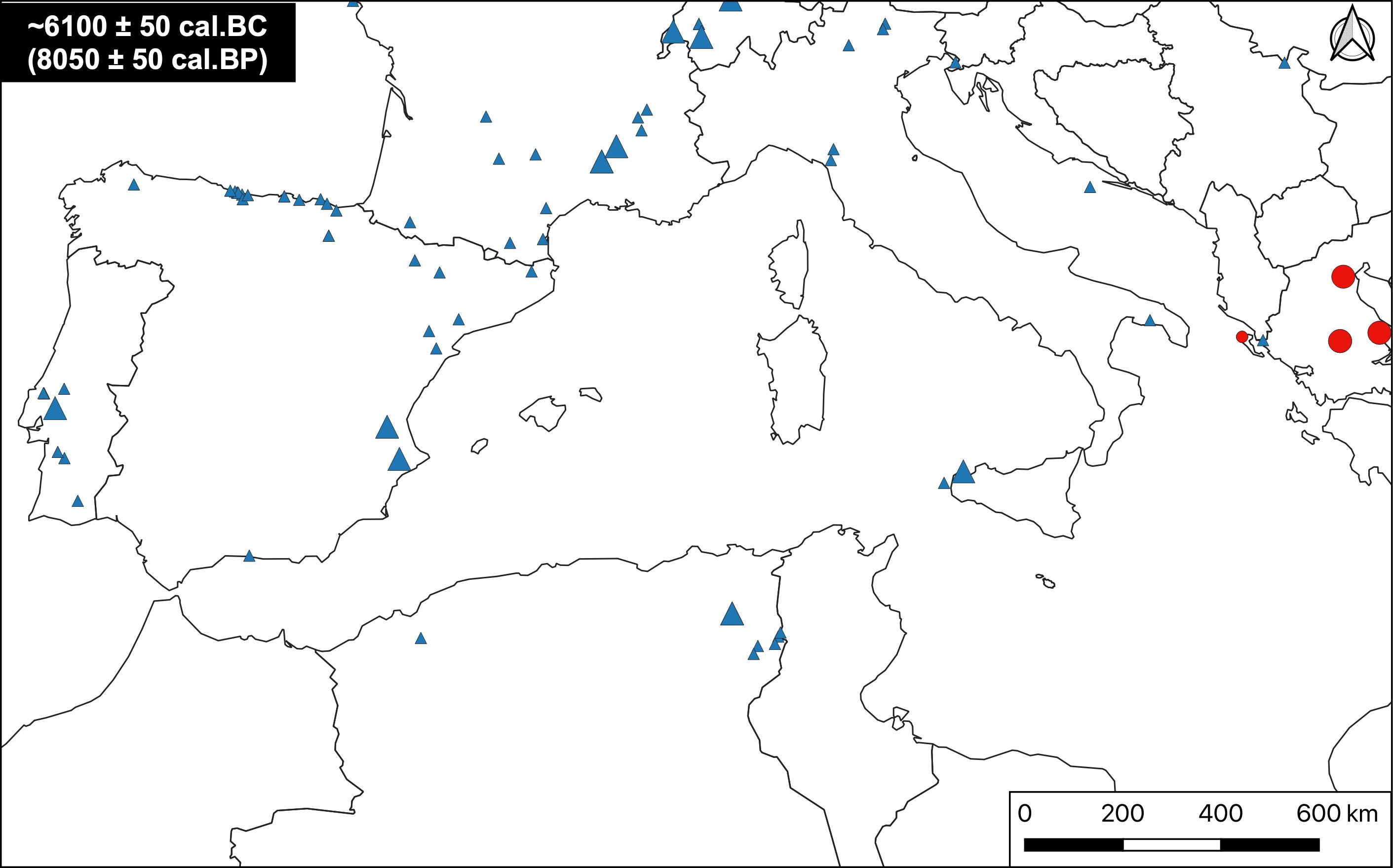

Supplement: S2 File — The small full colored symbols relate to occupations with a reliability value of 2, the large ones are reliability 1. Countries boundaries are from Natural Earth (free vector and raster map data @ naturalearthdata.com). (ZIP) [file pone.0246964.s004.zip › 6100-rel1_2.jpg]

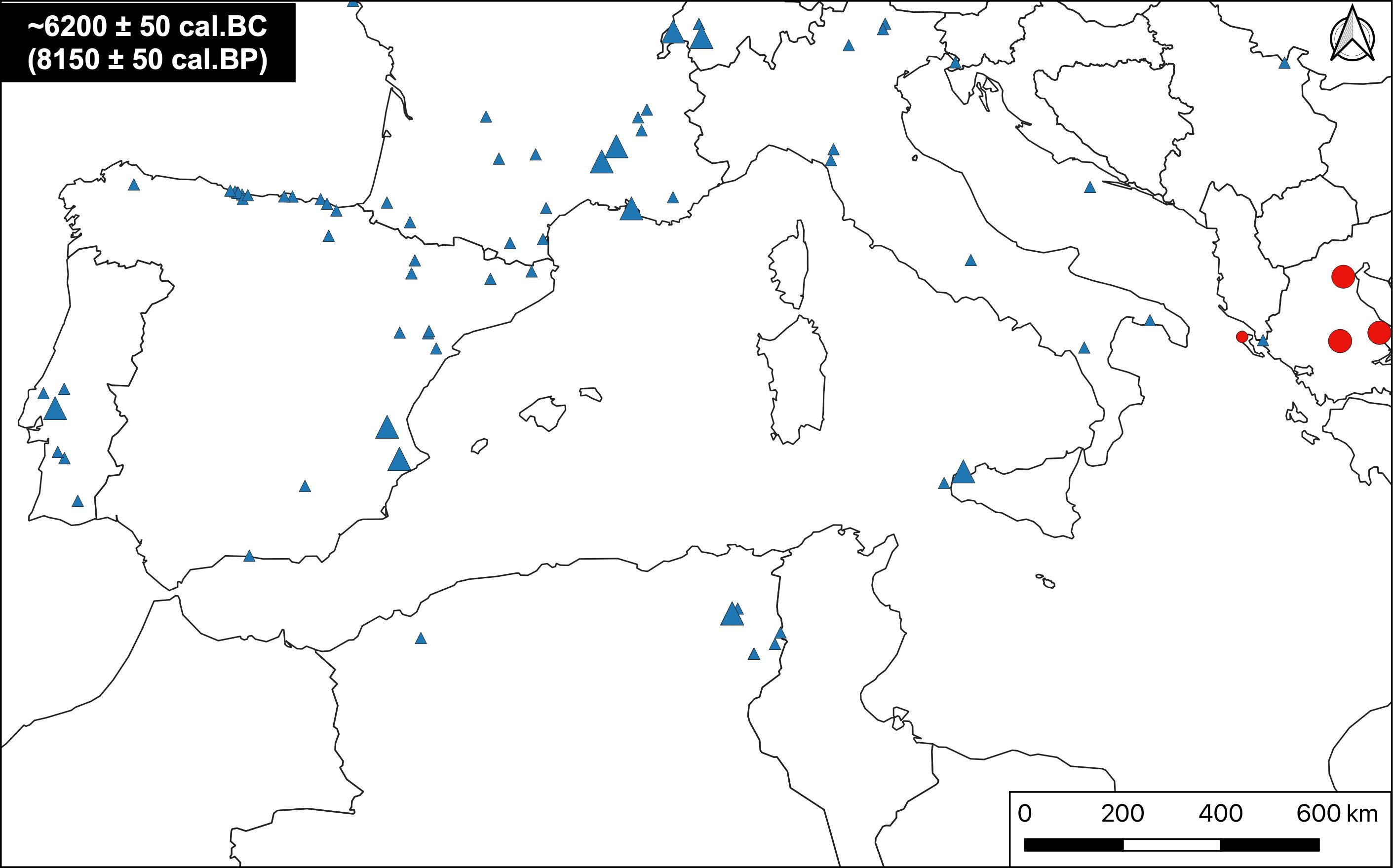

Supplement: S2 File — The small full colored symbols relate to occupations with a reliability value of 2, the large ones are reliability 1. Countries boundaries are from Natural Earth (free vector and raster map data @ naturalearthdata.com). (ZIP) [file pone.0246964.s004.zip › 6200-rel1_2.jpg]

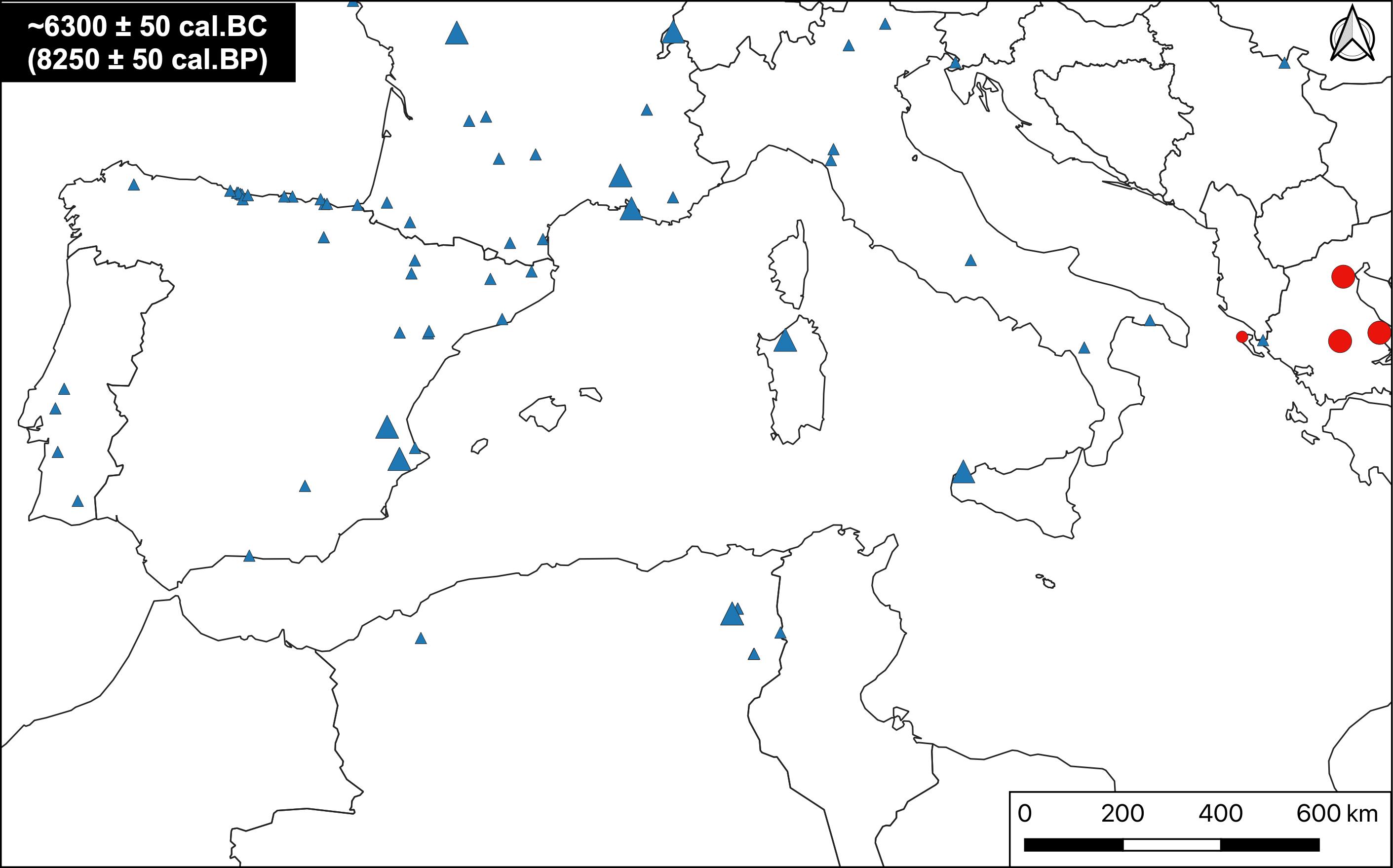

Supplement: S2 File — The small full colored symbols relate to occupations with a reliability value of 2, the large ones are reliability 1. Countries boundaries are from Natural Earth (free vector and raster map data @ naturalearthdata.com). (ZIP) [file pone.0246964.s004.zip › 6300-rel1_2.jpg]

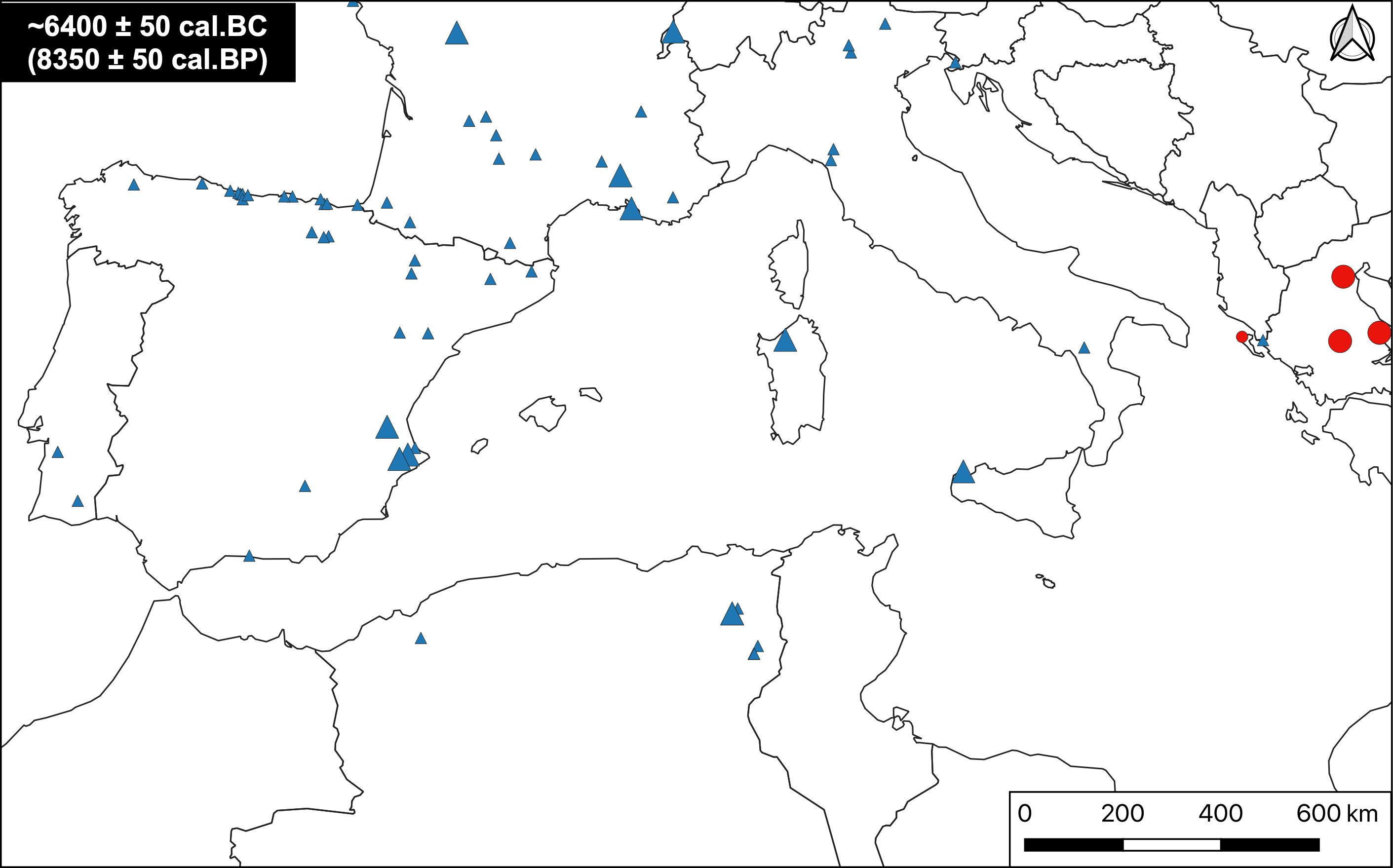

Supplement: S2 File — The small full colored symbols relate to occupations with a reliability value of 2, the large ones are reliability 1. Countries boundaries are from Natural Earth (free vector and raster map data @ naturalearthdata.com). (ZIP) [file pone.0246964.s004.zip › 6400-rel1_2.jpg]

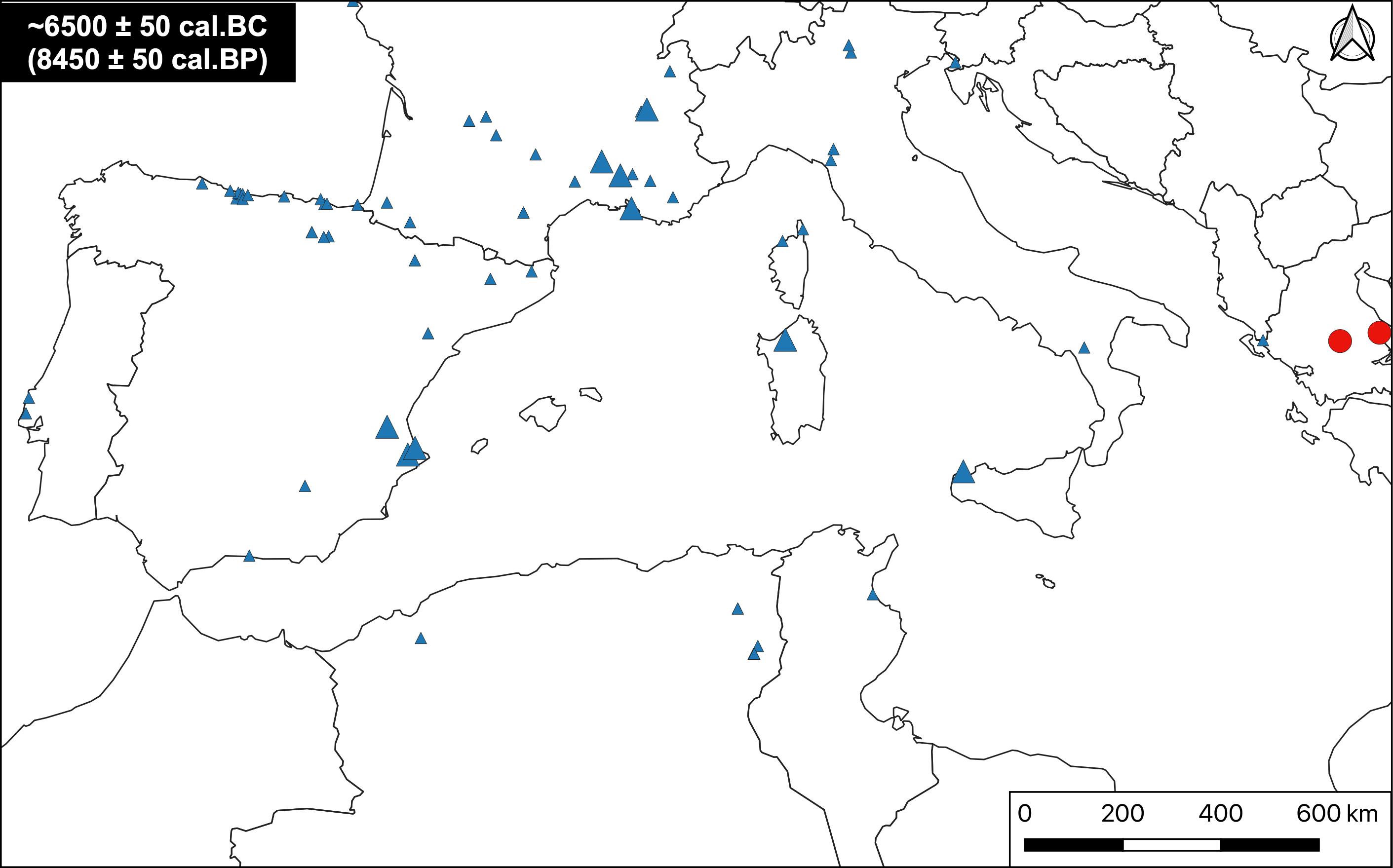

Supplement: S2 File — The small full colored symbols relate to occupations with a reliability value of 2, the large ones are reliability 1. Countries boundaries are from Natural Earth (free vector and raster map data @ naturalearthdata.com). (ZIP) [file pone.0246964.s004.zip › 6500-rel1_2.jpg]

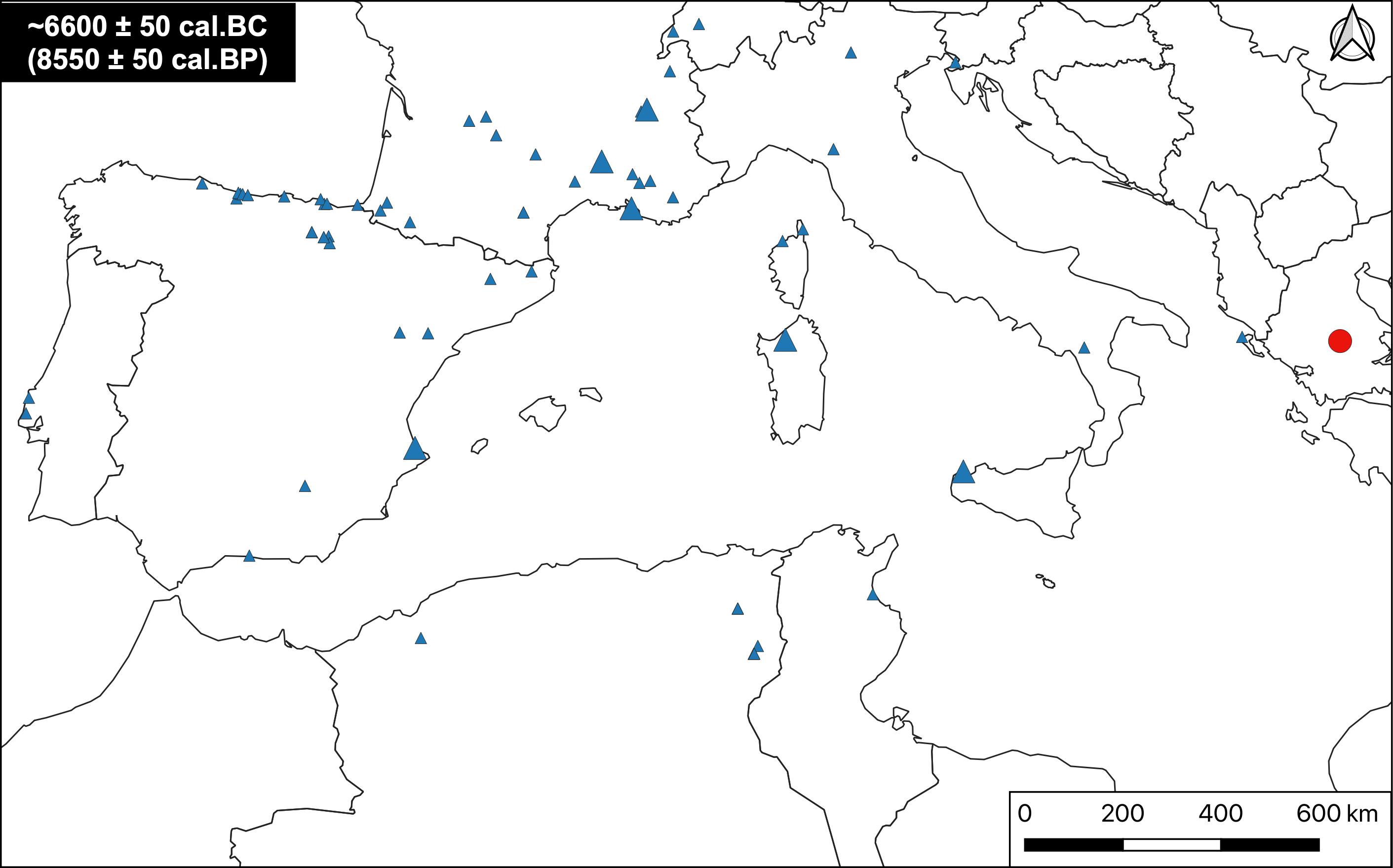

Supplement: S2 File — The small full colored symbols relate to occupations with a reliability value of 2, the large ones are reliability 1. Countries boundaries are from Natural Earth (free vector and raster map data @ naturalearthdata.com). (ZIP) [file pone.0246964.s004.zip › 6600-rel1_2.jpg]

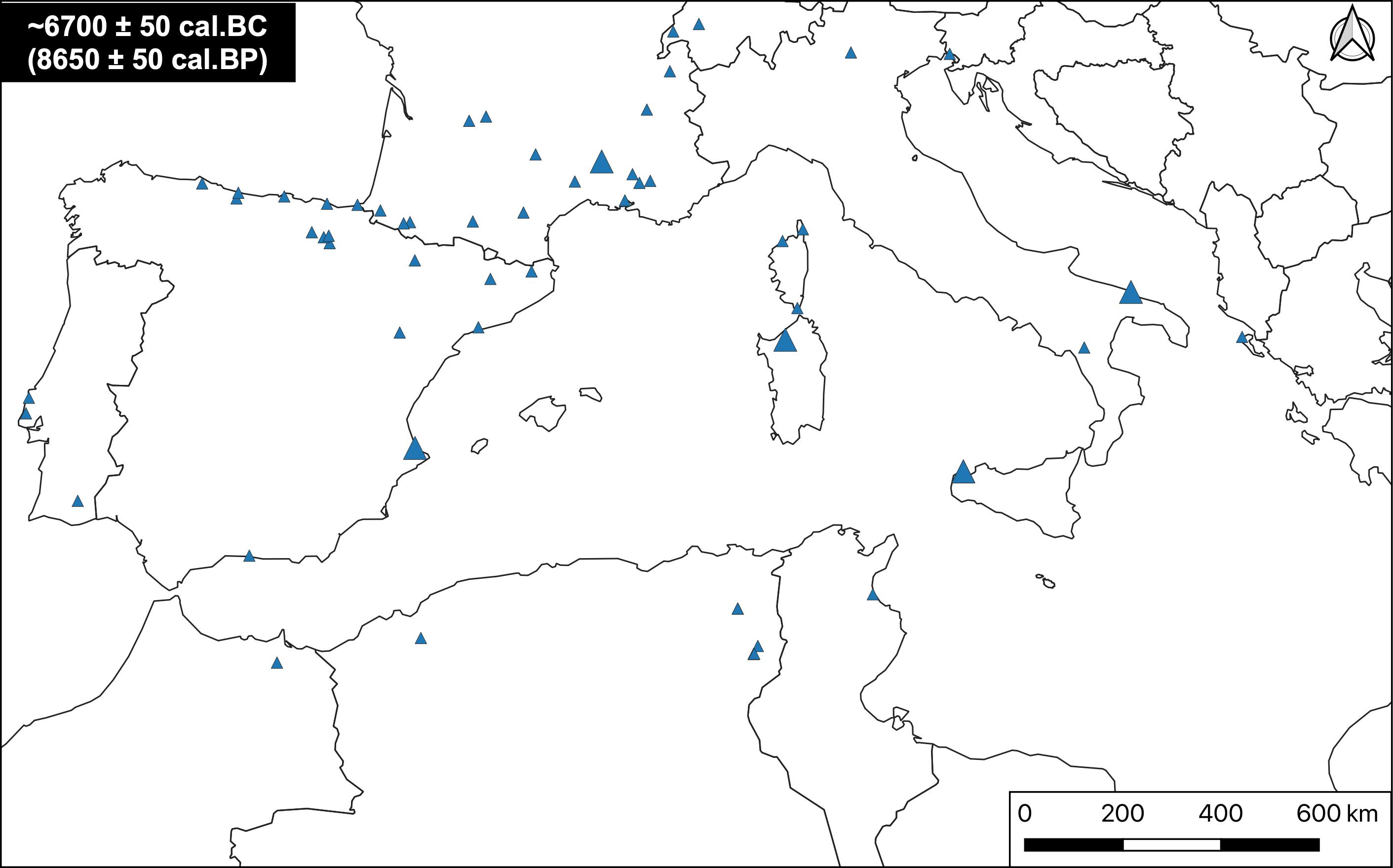

Supplement: S2 File — The small full colored symbols relate to occupations with a reliability value of 2, the large ones are reliability 1. Countries boundaries are from Natural Earth (free vector and raster map data @ naturalearthdata.com). (ZIP) [file pone.0246964.s004.zip › 6700-rel1_2.jpg]

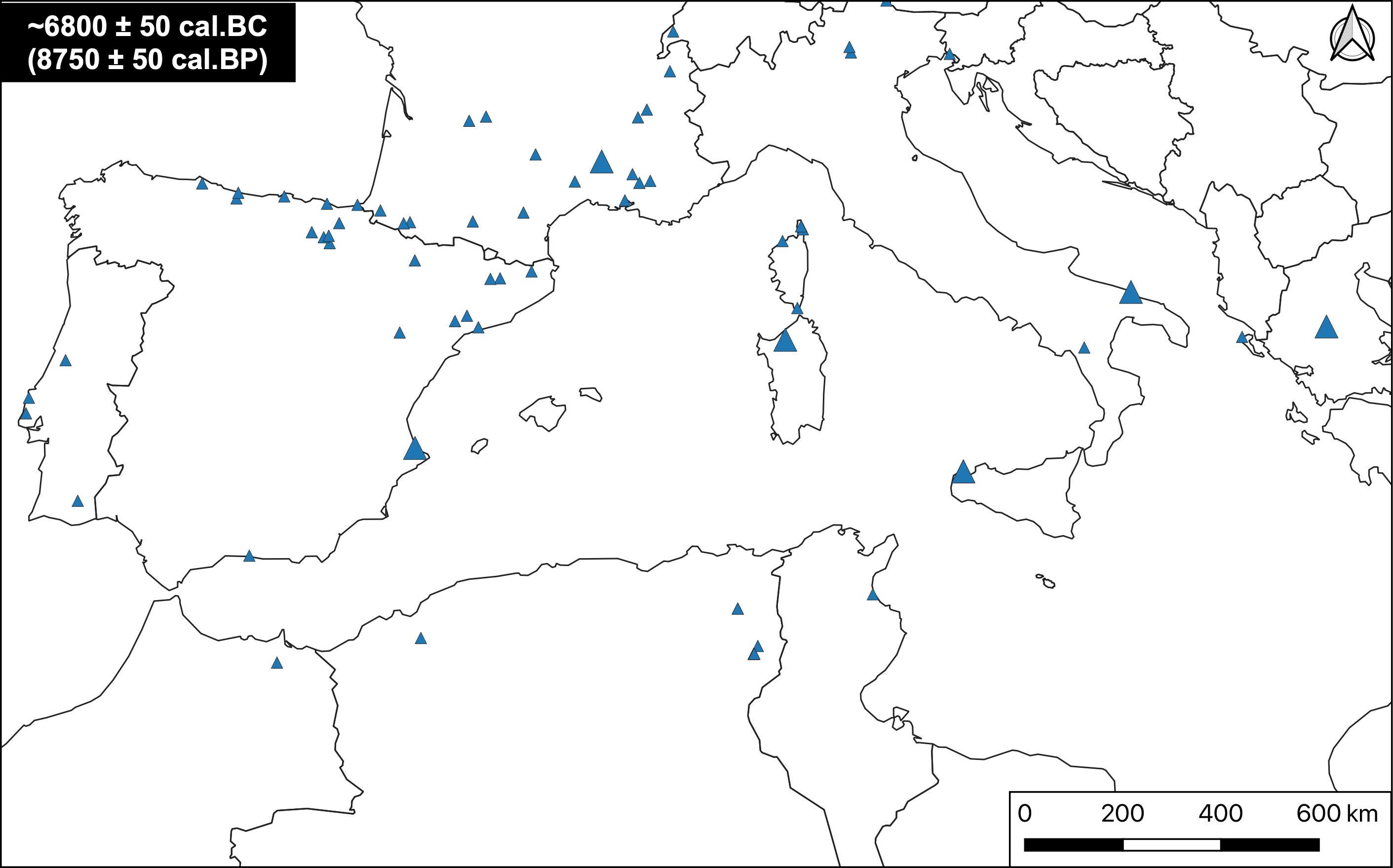

Supplement: S2 File — The small full colored symbols relate to occupations with a reliability value of 2, the large ones are reliability 1. Countries boundaries are from Natural Earth (free vector and raster map data @ naturalearthdata.com). (ZIP) [file pone.0246964.s004.zip › 6800-rel1_2.jpg]
